# A Review on Modeling Cure Kinetics and Mechanisms of Photopolymerization

**DOI:** 10.3390/polym14102074

**Published:** 2022-05-19

**Authors:** Margit Lang, Stefan Hirner, Frank Wiesbrock, Peter Fuchs

**Affiliations:** 1 Polymer Competence Center Leoben, 8700 Leoben, Austria; peter.fuchs@pccl.at; 2 Institute for Chemistry and Technology of Materials, University of Technology Graz, NAWI Graz, 8010 Graz, Austria; stefan.hirner@pccl.at (S.H.); f.wiesbrock@tugraz.at (F.W.)

**Keywords:** free-radical photopolymerization, cationic photopolymerization, mechanistic model, phenomenological model, reaction kinetics

## Abstract

Photopolymerizations, in which the initiation of a chemical-physical reaction occurs by the exposure of photosensitive monomers to a high-intensity light source, have become a well-accepted technology for manufacturing polymers. Providing significant advantages over thermal-initiated polymerizations, including fast and controllable reaction rates, as well as spatial and temporal control over the formation of material, this technology has found a large variety of industrial applications. The reaction mechanisms and kinetics are quite complex as the system moves quickly from a liquid monomer mixture to a solid polymer. Therefore, the study of curing kinetics is of utmost importance for industrial applications, providing both the understanding of the process development and the improvement of the quality of parts manufactured via photopolymerization. Consequently, this review aims at presenting the materials and curing chemistry of such ultrafast crosslinking polymerization reactions as well as the research efforts on theoretical models to reproduce cure kinetics and mechanisms for free-radical and cationic photopolymerizations including diffusion-controlled phenomena and oxygen inhibition reactions in free-radical systems.

## 1. Introduction

Photopolymerization is a method for manufacturing highly-crosslinked polymer networks, in which the initiation of a chemical-physical reaction occurs by exposing photosensitive, monofunctional or multifunctional monomers to a high-intensity, generally ultraviolet (UV), light source. According to Decker [1], photopolymerization is one of the most efficient methods for achieving quasi-instantaneous polymerization. Its huge potential in the simple and fast production of materials with special properties leads to a wide range of potential applications [2]. Practical applications include, for instance, coatings [1,3], tissue engineering [4,5], photolithography [6,7,8,9], microfluidic device fabrication [10,11], 3D prototyping [12,13,14,15], and 4D bioprinting [16,17]. Significant characteristics of photopolymerization include the solvent-free formulation, ability to cure at ambient temperature conditions (which is especially important for heat-sensitive materials), and low energy consumption, as well as spatial and temporal control over the polymerization [1]. Furthermore, the temperature rise resulting from the exothermic nature of the reaction can be controlled by changing the irradiation intensity and wavelength [18]. However, there are also a number of problems and complexities associated with photopolymerization including volume shrinkage and stress, the presence of unreacted, potentially extractable polymer, and the susceptibility to oxygen inhibition in the case of free-radical photopolymerizations [19,20].

One decisive factor in photopolymerizations is the curing degree achieved, as it significantly affects the mechanical properties of the polymers. The curing degree α is zero at the beginning of the chain-growth or crosslinking reaction. It increases with time in the course of the photopolymerization process due to the growth of the polymer chains.

**Figure 1:**
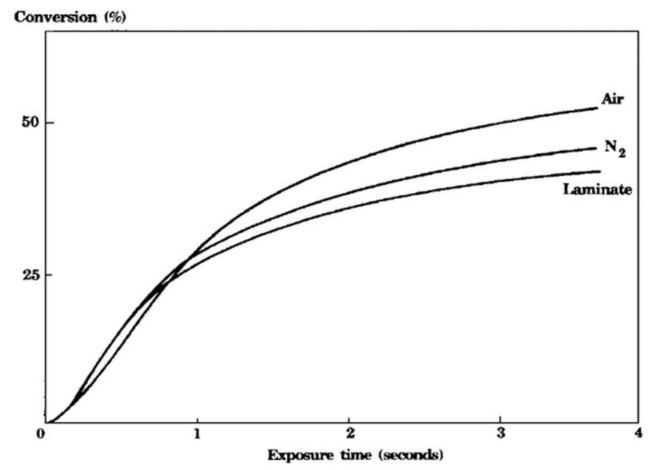
Conversion versus exposure time for the cationic photopolymerization of a cycloaliphatic diepoxy compound in the presence of air, in a $N_{2}$-saturated atmosphere, and in the presence of air after covering with a transparent polyethylene film (laminate). Reprinted with permission of John Wiley and Sons from reference [25].

Photocurable resins can be divided into two major classes, differing basically by their polymerization mechanism: photoinitiated free-radical polymerizations (such as the polymerizations of acrylates) and photoinitiated cationic polymerizations (such as the polymerizations of epoxides, lactones and vinyl ethers, which are inactive towards radicals) [21]. The latter have the distinct advantage that they lack sensitivity towards atmospheric oxygen whereas the loss of radicals to oxygen, known as oxygen inhibition, is a problem that is pervasive in free-radical photopolymerization [22,23,24]. Figure 1 shows the conversion versus the exposure time for a cycloaliphatic diepoxy compound recorded in the presence of air, in a $N_{2}$-saturated atmosphere and in the presence of air after covering the monomer with a transparent polyethylene film (laminate). The kinetic curves clearly show the lack of sensitivity towards oxygen for cationic photopolymerizations, as the epoxy monomer polymerized at essentially the same rate independent of the experimental conditions [25]. Operating in a $N_{2}$-saturated atmosphere and as a laminate, the polymer even contains a larger amount of residual monomer, i.e., the extent of conversion is lower compared to operating in the presence of $O_{2}$.

The lack of sensitivity towards oxygen further implies that, in contrast to free-radical polymerization, the chain reactions continue to develop in the dark even in the presence of air [25] and even in shadow regions (regions that had no illumination) [26]. This “dark reaction” or “dark curing” offers the possibility of curing residual monomers in order to achieve complete conversion in cationic polymerization [27]. According to Corcione et al. [28], the “dark reaction” in free-radical polymerization resins is negligible. The “dark reaction”, which takes place after the termination of UV exposure, is due to the fact that, unlike radicals in free-radical photopolymerization, two cations cannot interact to undergo combination or disproportionation and, hence, such types of termination are generally suppressed. The living polymer chain will continue to grow in the dark until termination occurs by transfer reaction or bimolecular interaction with another species present in the polymerization mixture (e.g., water or bases added/present in the reaction mixture) [29].

According to Lin et al. [30], oxygen inhibition plays a critical role, especially for optically thin polymers. However, various strategies to reduce oxygen inhibition in photopolymerizations are proposed in the literature [31,32]: (1) using a higher light dose or intensity, (2) using a higher photoinitiator concentration, (3) using co-initiators, (4) addition of radical scavengers, (5) working in an inert environment, and (5) chemical mechanisms such as the thiol-ene and thiol-acrylate-Michel systems which are insensitive to oxygen.

According to Kim et al. [29], cationic photopolymerization is becoming increasingly important due to its applicability to rapid prototyping, i.e., the technique of stereolithography. Although there is increased interest in cationic photopolymerizations, free-radical photopolymerization is still the most popular and most widely used type [33]. According to Decker et al. [25], one of the main limitations of photoinitiated cationic polymerization is the relatively low cure speed, compared to the very reactive acrylic systems polymerized by free radical photoinitiated polymerization. Increasing the intensity of the UV radiation, by using lasers, is a possible way to overcome this issue [34,35].

Besides free-radical and cationic photopolymerizations, so-called hybrid systems have been reported as well, which implies mixing monomers that polymerize by different mechanisms, i.e., in the presence of both, radical and cationic photoinitiators [36]. Using such hybrid systems offers the possibility of producing interpenetrating polymer networks within a few seconds of UV irradiation. Additionally, by a proper selection of the two components, the final properties of the cured polymer can be controlled precisely [1].

**Figure 2:**
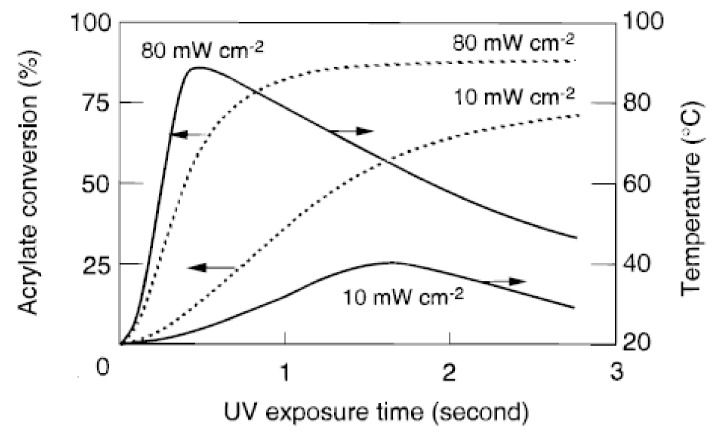
Temperature profile vs. exposure time (solid line) and conversion profile vs. exposure time (dashed line) for different UV light intensities of photopolymerization of polyurethane-acrylates. Reprinted with permission of John Wiley and Sons from reference [1].

Compared to thermal curing, photopolymerizations reach higher conversions as volume shrinkage occurs over a much longer timescale than the chemical reaction, i.e., a temporary excess of free volume is generated. The heat instantly evolved by the exothermic reaction results in an increase in the sample temperature and therefore contributes to a higher final degree of conversion [1]. Furthermore, one of the distinct advantages of photoinduced polymerizations is the precise control over the initiation step with respect to onset and end of the period of initiation as well as its magnitude (light intensity). To evaluate the contribution of increasing light intensity, Decker et al. [1] recorded temperature profiles of samples undergoing photopolymerization by real-time Fourier-transformed infrared (RT-FTIR) spectroscopy. The temperature profiles along with the conversion profiles for polyurethane-acrylates at two different UV radiation light intensities ($10\;{\mathrm{mW}\;\mathrm{cm}}^{-2},80\;{\mathrm{mW}\;\mathrm{cm}}^{-2}$) are shown in Figure 2. An increase in light intensity obviously leads to a faster polymerization and a more extensive cure; the final product contains a lower amount of unreacted functional groups. Decker and co-authors reported that a higher initial light intensity $I_{0}$ led to an increase in sample temperature which in turn provided more molecular mobility and, consequently, led to higher ultimate conversion. As can be seen in Figure 2, the temperature starts to rise as soon as the polymerization reaction begins and reaches its maximum value once the polymerization reaction starts to slow down due to gelation. The temperature decreases slowly as air cooling becomes predominant over the exothermic polymerization reaction at that stage.

The properties and structure of polymeric materials are governed to large extent by the kinetics of their synthesis, in general highly non-equilibrium polymerization processes [37]. To determine the kinetics of photoinitiated polymerizations, two techniques are widely used, namely RT-FTIR spectroscopy and photodifferential scanning calorimetry (photo-DSC). Photo-DSC, which monitors the photopolymerization reaction’s heat flow rate over time, is by far the most widely used technique in photocuring kinetic studies [20,33,38]. However, its main limitation lies in the relatively long response time, requiring operation with low-intensity UV radiation. Due to its time resolution in the range of milliseconds, real-time FTIR is well suited to evaluate important kinetic parameters of ultrafast (intense UV or laser irradiation) crosslinking polymerization reactions [1]. FTIR directly records conversion versus time curves.

In order to study material property changes during the photopolymerization process, it is necessary to model the kinetics of the photopolymerization’s chemical reactions and describe the evolution of the curing solution’s species composition. Existing models to analyze photopolymerizations can be broadly classified as energetic approaches, mechanistic approaches and phenomenological approaches [39]. Mechanistic kinetic models offer a number of distinct advantages over phenomenological kinetic models. For instance, mechanistic models offer the possibility to treat the effect of the type, concentration or number of initiators on the overall curing rate separately, once the values of the various rate constants (e.g., initiation, propagation, termination) have been determined. Subsequently, there is no need to conduct curing experiments each time the type, concentration or number of initiators is changed, as is the case for phenomenological models. Approaches to obtain reasonably accurate and realistic kinetic expressions are discussed in detail in Section 3. Furthermore, approaches regarding the implementation of kinetic models through numerical simulations are shown in Section 4.

Due to the increasing interest in using nanocomposites to improve and tailor-fabricate material properties, there is a demand for synthesizing nanocomposites with a uniform distribution of nanoparticles in the polymer matrix. However, due to effects such as high surface energy, unusual chemical activity, and immiscibility of the nanoparticles and the monomer formulation/polymer matrix, nanoparticles tend to form large aggregates (already) in the monomer solution, resulting in non-uniform materials with deteriorated physicochemical characteristics [40]. Thus, one key point in the preparation of polymer nanocomposites is the selection of a polymerization technique that ensures the fixation of the initial uniform distribution of nanoparticles in the final nanocomposite, preventing the agglomeration of nanoparticles during the polymerization process. Frontal polymerization proved to be a positive technique, contributing to the uniform distribution of nanoparticles in the resulting polymer composite [41,42]. 

The main shortcoming of photopolymerization lies in the limited penetration depth, caused by decreasing light intensity along the height of the formulation to be cured, which greatly limits the application potential of photopolymerization to thin films and adhesive applications [27,43]. Layers with a typical thickness between 5 and 200 μm or, at the very most, a few millimeters thickness can be polymerized [1,44]. Light absorption causes the intensity of radiation to decay within the material according to the Beer–Lambert law [45]. The incident light is mainly absorbed by the photoinitiator in the top layer exposed to light, leading to a top-to-bottom gradient for the photogenerated initiating active species and, consequently, leading to a sharp depth of the cure profile within the sample undergoing polymerization. The presence of fibers as a reinforcing phase may even contribute to this shortcoming by further reducing the transmission of light [45,46]. Therefore, photopolymerization can be effectively used for curing thin samples, but it is not suitable for curing thick samples, especially those containing carbon fibers or other opaque materials [43]. According to Decker [1], photoinitiation has proven to be well suited to induce frontal polymerization, which is highly beneficial to curing thick specimens. The basic idea behind frontal polymerization will be explained in Section 6.

## 2. Materials and Curing Chemistry

### 2.1. Radical Polymerizations

**Figure 3:**
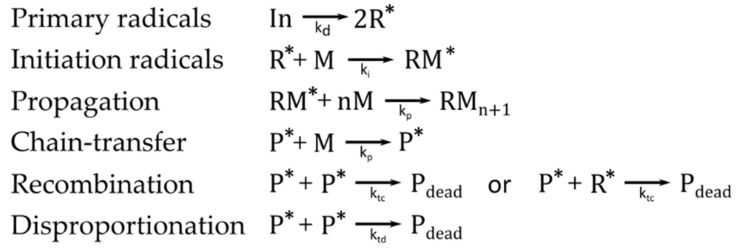
Mechanism of free-radical polymerizations, comprising the decomposition of the initiator, the formation of the initiation radicals, and the propagation, chain-transfer and terminating reactions.

Free-radical polymerizations are initiated by free radicals. The polymerization begins with the generation of primary radicals, which arise from the decomposition of the initiator, and subsequent addition, for example to a carbon–carbon double bond of a monomer, which yields the so-called initiation radicals [47]. The radicals are only released from the monomers themselves in very rare cases. Usually, initiator molecules are added that form the reacting species through thermochemical, electrochemical, and/or photochemical treatment. Basically, four different types of reactions can be associated with free radical polymerizations: generation of primary radicals from non-reactive species (initiation), radical addition to a suitable monomer (propagation), atom-transfer and atom abstraction reactions (chain-transfer reactions and disproportionation) and radical–radical recombination [48,49] (Figure 3). Disproportionation and recombination of macro-radicals or initiator radicals represent two types of termination reactions. These termination reactions are responsible for a low concentration of the active centers, on the order of about 10^−8^ ${\mathrm{mol}\;L}^{-1}$ [50]. Further restrictions on the reaction kinetics are caused by side reactions of the active species with the solvent, impurities, monomers, initiators and polymers, yielding radicals as well. These different radicals can influence the reaction and form polymers with different constitutions, configurations and molar mass distribution.

**Figure 4:**
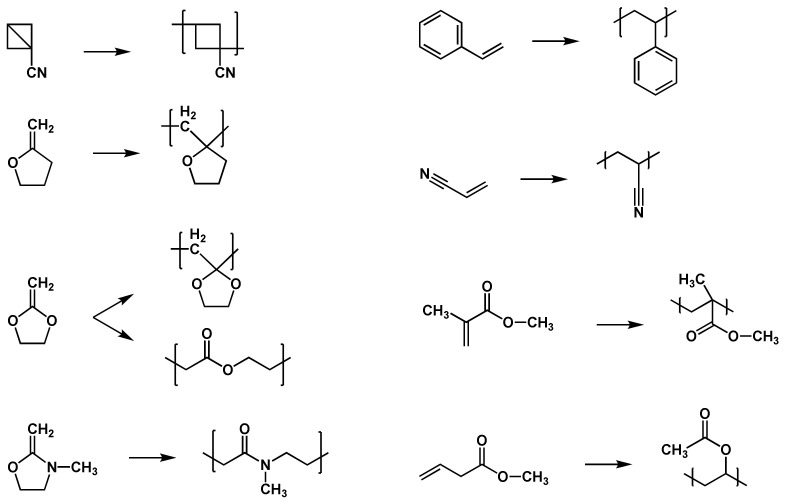
Representation of the chemical structures of monomers for free-radical polymerizations and of the corresponding repetition units.

Polar, steric, stabilization, and thermodynamic effects have further influence on the reaction and reactivity of the radicals or monomers [47]. In polar effects, for example, the nucleophilicity of the radical and the electrophilicity of the monomer play an important role. Due to the fact that head-to-head additions and head-to-tail additions are the most common propagation reactions, steric effects of the monomers and radicals have great influence. Stabilization effects occur if delocalization of the unpaired radical electron is possible. The higher the delocalization of the electron, the lower the reactivity of the radical. In principle, two types of monomers are able to perform free-radical polymerizations: monomers bearing high-tension saturated rings or unstressed unsaturated rings, as well as monomers bearing unsaturated bonds, i.e., double bonds [50]. Typical monomers for free-radical polymerizations are alkenes, which are shown with other examples in the list below (Figure 4).

#### 2.1.1. Thermally-Initiated Radical Polymerizations

In so-called real thermal polymerizations, the polymerization reactions of monomers take place with the complete exclusion of external initiators such as atmospheric oxygen, light sources and other impurities. Therefore, it is a spontaneous or self-initiated polymerization without the targeted addition of initiators. A real thermal polymerization is likely to occur in styrene, some of its derivatives, 2-vinylpyridine, 2-vinylfuran, acenaphthylene, methyl acrylate and others [50].

**Figure 5:**
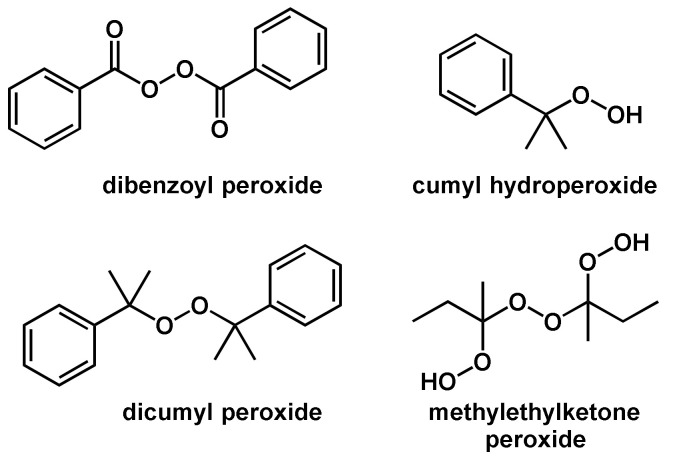
Chemical structures of most commonly used organic peroxide initiators.

**Figure 6:**
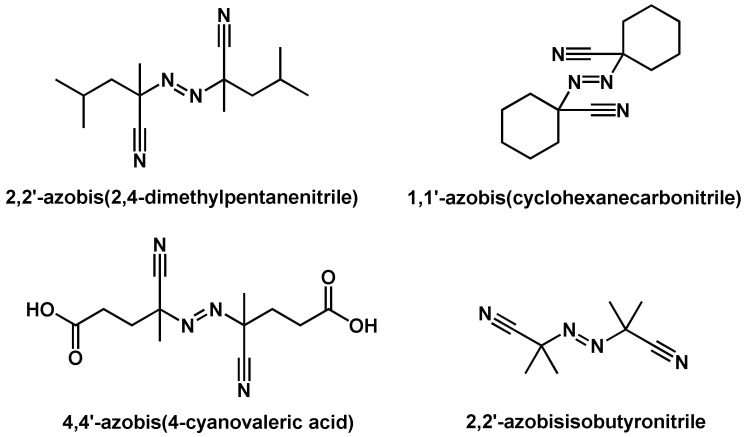
Chemical structures of most commonly used azo initiators.

In general, the thermal self-initiated polymerization is very rare and, therefore, thermal homolytic dissociation of an initiator is the most common way to create reactive radical species. The energy required to homolytically split a covalent bond can be brought in by means of thermal, chemical and photochemical energy. In the case of thermal energy, a so-called thermally initiated or thermally catalyzed polymerization reaction takes place. Compounds used as thermal initiators commonly have bond dissociation energies in the range of 100–170 ${\mathrm{kJ}\;\mathrm{mol}}^{-1}$ [51]. Compounds that are not included in this dissociation energy range either have too slow or too high dissociation speeds and are commonly not practicable in polymerization reactions. Only a few compounds have bonds that are homolytically cleavable in this energy range; these mainly have the O–O, S–S, or N–O bonds [51]. The most commonly used initiators in thermal initiated radical polymerization reactions are classified as organic peroxides including dicumyl peroxide, dibenzoyl peroxide, cumyl hydroperoxide and methylethylketone peroxide (Figure 5). In addition to the abundantly used organic peroxides, also so-called azo compounds are also often used as initiators. Examples of such azo compounds are 2,2′-azobisisobutyronitrile (AIBN), 2,2′-azobis (2,4-dimethylpentanenitrile), 4,4′-azobis (4-cyanovaleric acid), and 1,1′-azobis(cyclohexanecarbonitrile) (Figure 6). It should be noted that the driving force for the homolytic dissociation of azo compounds is the formation of the very stable nitrogen molecule due to the fact that the C–N bond dissociation energy is relatively high [51].

#### 2.1.2. Photoinitiated Radical Polymerizations

In recent years, photoinitiated polymerizations have become an important and efficient polymerization type that has several advantages including the possibility of solvent-free formulations, low-temperature conditions, and the spatial control of the light penetration inside the photosensitive resin [52,53]. In principle, radical photoinitiators are compounds that decompose into radicals during exposure to shortwave or visible light. By absorbing the energy of the radiation, the π-electrons of the photoinitiator are raised to a higher level. The π* excited state has a short lifespan only; it nonetheless offers the time range for the molecule to decompose into radicals. These radical PIs can be distinguished into two different systems, type I (decomposition) and type II (decomposition and subsequent abstraction of protons). In type I, also related to as Norrish I cleavage, a light-induced photolytic α-cleavage yields two radicals [54].

**Figure 7:**
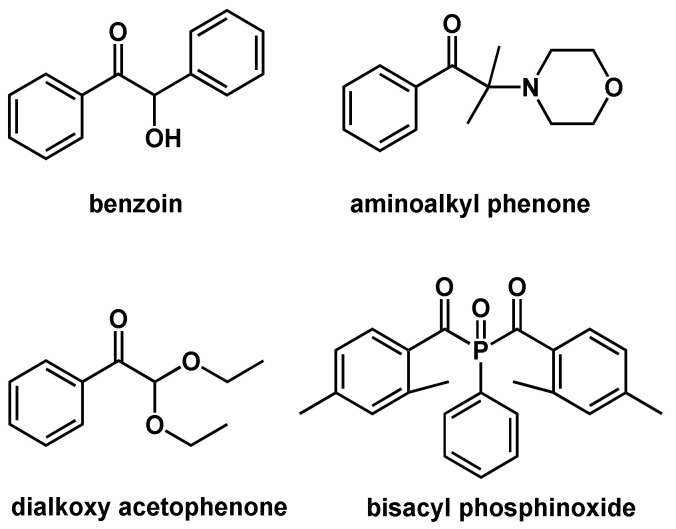
Chemical structures of common type I photoinitiators.

Common type I photoinitiators are benzoin, its derivatives, dialkoxy acetophenones, aminoalkyl phenones, and bisacyl phosphinoxides (Figure 7). The main drawback of type I photoinitiators is their irreversible consumption during photopolymerization [53]. In type II photoinitiators, also referred to as H-abstraction type initiators, the photoinitiator is able to abstract a hydrogen atom from adjacent molecules, forming two radicals.

**Figure 8:**
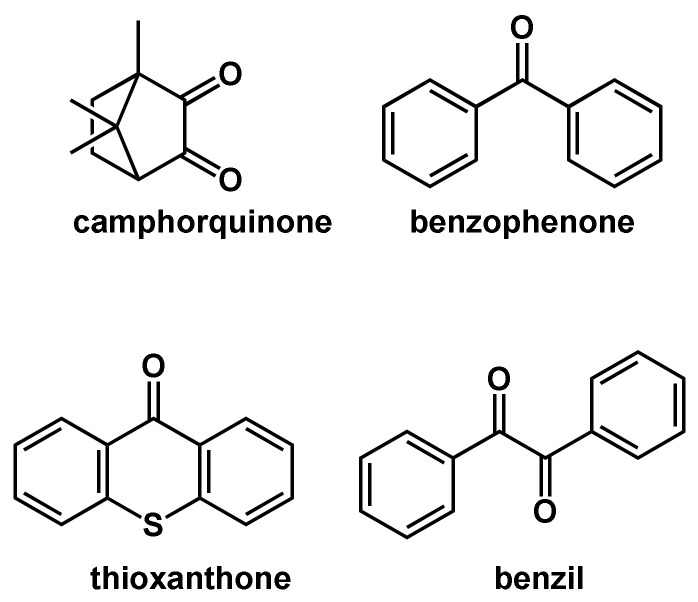
Chemical structures of common type II photoinitiators (the proton donors are not represented here).

Common type II photoinitiators are benzophenone, benzil, camphorquinone and thioxantone in combination with proton donors (Figure 8). Photolytic cleavage of such compounds in the presence of a hydrogen donor yields a ketyl radical and an additional radical deduced from the hydrogen donor [55]. Due to the stabilization effect, ketyl radicals are very stable radicals and normally do not react in the polymerization reaction [56].

### 2.2. Cationic Polymerizations

Cationic polymerizations are chain-growth polymerizations composed of addition reactions of electrophilic and nucleophilic species. Initiators are the electrophilic part E^+^, whereas the monomer is the electron-donating part. These reactions are started by the addition of the positively charged electrophile E^+^ to the electron-donating monomer, resulting in a monomer cation EM^+^, often a carbocation, which is an activated monomer that performs the propagation reaction [50]. Polymerizable monomers can be distinguished into two different classes: ethylenic monomers, in which the reactive species is the carbocation, and heterocyclic monomers, in which the reactive species is the onium ion. The viable part of the monomer must be the most nucleophilic part. As an example, acryl nitrile consists of a vinyl group and a nitrile group; the nitrile group has the more pronounced nucleophilic character. However, due to the resonance stabilization, no cationic polymerization is possible [50].

In general, three different classes of initiators, BrØnsted acids, Lewis acids, and carbonium salts, are often used as initiators. The counter anions must not be nucleophilic, as they would otherwise induce chain termination. However, since the monomer cations are highly reactive, and therefore also unstable, an increased number of termination reactions and chain-transfer reactions occur. In addition, a solvent is required for almost all cationic polymerizations. Therefore, cationic polymerization is used in only a few industrial applications.

#### 2.2.1. Cationic Polymerizations Initiated by Carbonium Ions, BrØnsted Acids, or Lewis Acids

**Figure 9:**
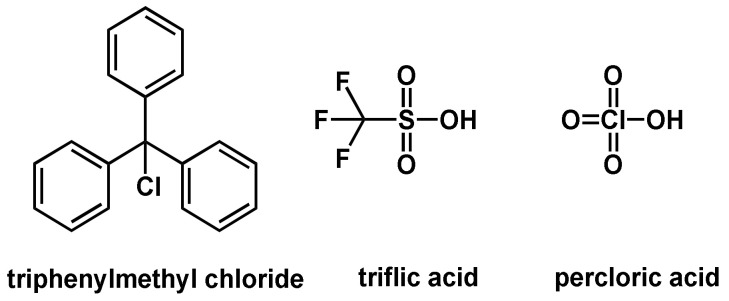
Chemical structures of common types of cationic initiators.

Three different initiation classes, BrØnsted acids, Lewis acids, and carbonium salts are usually used in cationic polymerization reactions. BrØnsted acids and protonic acids are able to start the polymerization reaction by protonation (of, e.g., the olefin). Acids with very low pk_a_ values, which are associated with a low basicity of the counterions, are required to protonate enough monomers to subsequently start the chain-growth reaction. Another important requirement is the weak nucleophilicity of the counterions, which otherwise would lead to chain termination. Commonly used BrØnsted acids for the initiation of a cationic polymerization are perchloric acid, trifluoromethane sulfonic acid (triflic acid), methane sulfonic acid and fluorosulfonic acid (Figure 9).

Lewis acids are another type of cationic initiator. Some of the Lewis acids, mostly metal halides, are able to carry out self-ionization (2 AlCl_3_ ↔ [AlCl_2_]^+^[AlCl_4_]^−^), which initiates the reaction. Examples of such metal halides are AlCl_3_, TiCl_4_, PF_5_, SnCl_4_, and I_2_ [50]. However, the efficiency of self-ionization is extremely low, and the polymerization, therefore, suffers from incomplete and slow initiation. To overcome this low efficiency, a so-called co-catalysis initiation mechanism is preferred. A Lewis acid, representing an activator, is mixed with a protonogen (H_2_O) yielding a complex that initiates the polymerization reaction. The polymerization rate is depending on the amount of the protonogen, due to the possibility of chain termination by the protonogen [57].

Another way of initiation is the use of stable carbonium salts. An example of this type of initiator is trityl chloride, which dissociates into a tripenhyl carbenium cation and a chloride anion. The reactivity of the carbonium ion can be increased by complexing the counterion, e.g., the chloride anion can be trapped in a [SbCl_6_]^−^ complex by adding SbCl_5_; thus, the termination of the chain growth can be reduced [50].

#### 2.2.2. Photoinitiated Cationic Polymerizations

**Figure 10:**
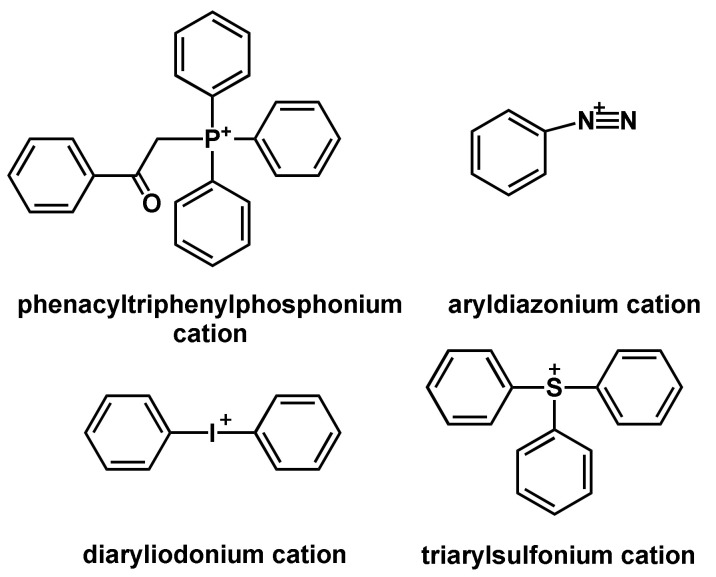
Chemical structures of common types of photoinitiators.

The photoinitiated cationic polymerization is a widely used technique today and was advanced in the 1970s by the discovery of photoacid generators (PAGs) by Crivello [58]. In principle, photolysis of the photoinitiator is achieved by irradiation with UV light, due to which superacids are formed that can initiate the polymerization [59]. Common cationic photoinitiators are diaryliodonium salts, triarylsulfonium salts, diazonium salts and their derivatives (Figure 10) [60,61].

As mentioned before, the counterion plays an important role in the activity and stability of the formed carbonium cation and the polymerization efficiency. Large anions such as SbF_6_^−^, AsF_6_^−^ and PF_6_^−^ are very weak nucleophiles due to the charge distribution. Therefore, superacids that are formed from PAGs are very stable initiators and have little tendency to chain termination reactions. The main drawbacks are the low solubility of the photoinitiators in non-polar monomers and the absorption band in the deep UV region, which does not overlap with the emission band of visible light [62].

### 2.3. Radical and Cationic Polymerizations Highlighted in This Review

This review is focused on the research efforts on analytical models to accurately describe the cure kinetics and mechanisms for curing behavior simulations of frontal polymerization reactions. Hence, the types of polymerizations that have been subjected to numerical analyses will be presented in this subchapter in brevity.

#### 2.3.1. Radical Polymerization of Acrylates

Acrylates and methacrylates are the esters of acrylic acid and methacrylic acid, respectively. Acrylates or methacrylates are a class of compounds that is most commonly polymerized using radical polymerization technology, constituting one of the most abundantly produced classes of polymers. (Meth)acrylates are one of the most reactive monomers in free-radical polymerization reactions. This high reactivity and diversity of chemical structures provide a variety of mechanical, physical, chemical and optical properties; the corresponding polymers and copolymers are used in a wide range of applications such as coatings and adhesives [63]. The main difference between acrylates and methacrylates is the lower toxicity and the lower reactivity of methacrylates. Commonly used monomers are methyl (meth)acrylate, ethyl (meth)acrylate, 2-ethylhexyl acrylate, hydroxyethyl methacrylate, and butyl (meth)acrylate, as well as acrylate-functionalized oligomers such as polyurethanes, polyesters, polyethers, and polysiloxanes [64].

**Figure 11:**
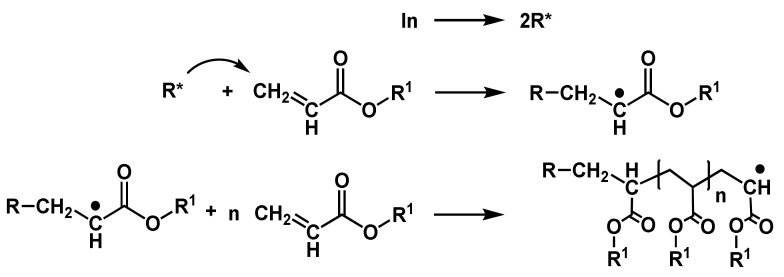
Schematic representation of the radical polymerization reaction of acrylates. In representing the initiator and R* the radical.

In principle, the radical polymerization of acrylates starts with the decomposition of the initiator yielding radicals. These radicals are able to attach to the vinyl group of the (meth)acrylate leading to the initiation radical and starting the propagation (Figure 11).

#### 2.3.2. Cationic Polymerization of Epoxides and Vinyl Ethers

**Figure 12:**
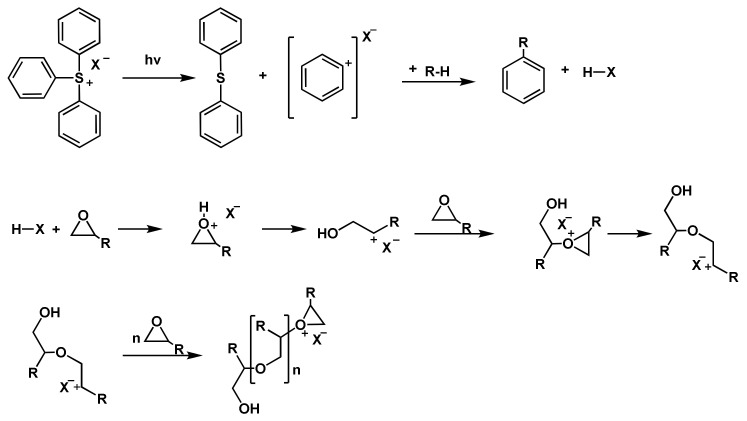
Reaction mechanism of the cationic ring-opening polymerization of epoxides.

Epoxy resins are one of the most commonly used thermosetting polymers and an important matrix for polymer composites [65]. The epoxy ring is highly strained and therefore exhibits high reactivity towards many substances [66]. The most important and commercially available epoxy resins are the diglycidyl ethers of bisphenol A (DGEBA) and bisphenol F (DGEBF). In Figure 12, the cationic polymerization of epoxide is shown. The reaction starts with the decomposition of the photoinitiator, yielding the superacid HX. The superacid protonates the epoxy ring, which subsequently ring-opens and reacts with a non-protonated epoxy monomer. This reaction is also known as ring-opening polymerization [67,68].

**Figure 13:**
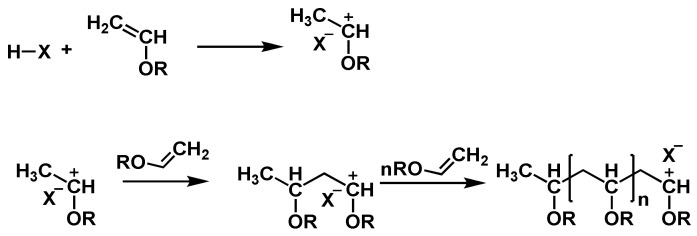
Schematic representation of the cationic polymerization of vinyl ethers.

Another important monomer class that polymerizes after cationic initiation is vinyl ethers. The decisive factor for this behavior is the strong electron-donating alkoxy substituent, which also renders anionic or radical polymerizations impossible [69]. Common vinyl ether monomers are ethyl vinyl ether (EVE), iso-butyl vinyl ether (IBVE), cyclohexyl vinyl ether, and hydroxy butyl vinyl ether (HBVE). An overview of the reaction mechanism is depicted in Figure 13. In principle, an acid is dissociated and protonates the vinyl ether yielding a carbocation. This carbocation starts to propagate. Notably, cyclic addition reactions, using a “cyclic initiator” are possible with vinyl ethers [70].

## 3. Models of Cure Kinetics

Curing behavior simulations require analytical models to accurately describe the complex reaction mechanism and kinetics as the system moves quickly from a monomer formulation to a polymer mixture. Models to describe the curing behavior can be broadly classified as energetic or kinetic models [39].

Energetic cure models, as the name already implies, describe photopolymerizations from an energetic point of view and are based on a direct relationship between the radiation intensity, radiation profile and energy. These models assume that the curing process starts when a critical value of energy, i.e., a material-dependent parameter, is reached. Consequently, using energetic models, the photopolymer is assumed to solidify, i.e., the resin is assumed to be cured, when, for instance, the irradiated energy into the resin reaches or surpasses a certain threshold value [71]. In 2010, Tomeckova et al. [72] presented a model for the photopolymerization of suspensions of ceramic powders in monomer solutions, based on a critical energy dose in terms of the relative number of photo-generated radicals and the concentration of inhibitors. The principal disadvantage of energetic models is that they provide information on whether curing is achieved or not, but they do not provide information about the curing degree, which, however, is crucial to correctly predict the mechanical performance of the polymer [39].

In contrast, kinetic models are capable of distinguishing between different degrees of cure, which in turn affect the mechanical properties of the polymer. Kinetic models, which are usually mechanistic (non-empirical) or phenomenological (empirical or semi-empirical) aim at predicting the evolution of the curing degree as a function of time.

Describing the photopolymerization from a mechanistic point of view implies that the complete scheme of consecutive and competitive reactions which take place during curing reaction (e.g., initiation, propagation, termination) is considered [73]. The curing degree is evaluated by solving several differential equations. The set of ordinary and/or partial differential equations is based on traditional mass action kinetics and can be used to define the dynamic concentration, i.e., the evolution of one or more reactant variables in the curing process as a function of time and space (depending on the model). To simplify the complexity of the curing reaction, usually, several assumptions are introduced for mechanistic models.

So-called phenomenological models are an alternative to mechanistic models. Phenomenological models are formulated in terms of the curing degree and are much easier to apply compared to mechanistic models [29]. The aim of phenomenological approaches is to describe the curing process with one reaction. Although several simultaneous reactions occur during the curing process a single simple empirical rate equation is used to describe the curing kinetics. The rate equation has the following general form [29] (Equation (1)),

(1) $$\frac{d\alpha }{dt}=K_{c}\left(T\right)f\left(\alpha \right)\;.$$

dαdt denotes the reaction rate, α denotes the curing degree, $K_{c}$ denotes a chemical-controlled rate constant as a function of temperature T, and $f\left(\alpha \right)$ is a function of the degree of cure. Therefore, phenomenological models ignore the chemical details and fit the data to a mathematical functional form where the constants of the model are determined based on experimental procedures.

Comparing the advantages and disadvantages of mechanical and phenomenological models, Matias et al. [74] suggest that the mechanistic approach is not practicable for engineering purposes, as the model equations typically require a large number of parameters that must be determined from experimental data fitting or through numerical optimization schemes in order to be solved. In contrast, phenomenological approaches usually require a limited number of parameters and are therefore considered as being simple and suitable for engineering applications. However, one major drawback associated with phenomenological models is the fact that these models capture the main features of reaction kinetics but ignore how individual species react with each other. For instance, phenomenological models are not capable of including the effect of the initiator (i.e., type, concentration or number of initiators) on the rate of cure. Consequently, the kinetic parameter, the rate constant $K_{c}\left(T\right)$ shown in Equation (1), needs to be recalculated by conducting curing experiments for each change of resin formulation [75]. In contrast, once the values of the various constants (e.g., for initiation, propagation and termination) are determined for a mechanistic model, the mechanistic model is capable of treating separately the effect of type, concentration or number of initiators on the overall curing rate [76,77].

Another limitation of phenomenological models is their inability to predict post-curing operations due to diffusion-controlled effects after vitrification [75]. Another alternative is stochastic models which are based on kinetic Monte Carlo simulations, for instance, proposed by Gillespie [78] or Ciftcioglu et al. [79]. These models determine the reaction sequence based on the probability of each possible event and can be used to predict the double bond conversion, molecular weight distribution and network connectivity [80].

The next sections briefly describe free-radical as well as cationic photopolymerization and present a comprehensive review of the principal theoretical models developed for predicting the kinetics of free-radical and cationic photopolymerization.

### 3.1. Free-Radical Photopolymerization

The traditional radical-initiated photopolymerization reaction can be divided into the following steps: photodecomposition, photoinitiation, propagation and chain transfer reactions and termination [81]. In a free-radical photopolymerization reaction, the following typical reactants can be found: initiator molecules In, free radicals generated by the photoinitiator $R^{*}$, monomers M, polymer chains P, oxygen $O_{2}$ and solvent. As light interacts with the photoinitiator molecules, the initiator molecule is decomposed yielding radicals. The asterisk * represents the active site of the radical species. Usually, one photoinitiator molecule decomposes into two radicals (Equation (2)):

(2) $$In\underset{k_{d}}{\to }2R^{*}.$$

When a free radical reacts with a monomer, it transfers its active center to the monomer and initiates a “macroradical”, to which monomers are added successively. An active site does not vanish, until it is terminated; therefore, the polymer chain is also in an active state (labeled by the asterisk *; Equation (3)):

(3) $$R^{*}+M\underset{k_{i}}{\to }\;RM^{*}.$$

The polymer chains propagate via reactions with other monomers or crosslinking with other polymer chains (in the case of multifunctional acrylates; Equation (4)):

(4) $$R-M_{}^{*}+n\;M\underset{k_{p}}{\to }RM_{n+1}^{*}\;\;\;\mathrm{or}\;\;\;\;P^{*}+M\underset{k_{p}}{\to }P^{*}.\;$$

**Figure 14:**
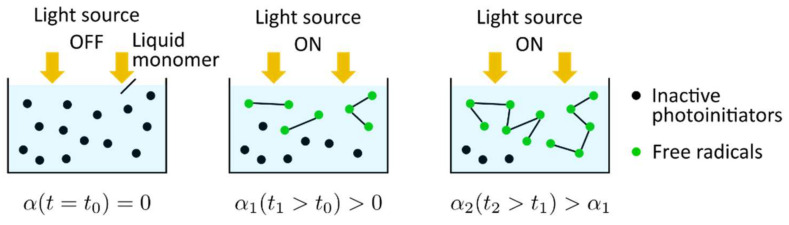
General photopolymerization scheme.

The general photopolymerization scheme is shown in Figure 14. At the initial state ($t=t_{0}$) the light source is off; the monomer is in a liquid phase ($\alpha \left(t=t_{0}\right)=0$), and the photoinitiators are in an inactive stage. When the monomer resin is irradiated ($t_{1}>t_{0}$), the photoinitiators are decomposed yielding free radicals that are capable of initiating the growth of a polymer chain. The polymer chain growth is quantified by the curing degree ($\alpha_{1}\left(t_{1}>t_{0}\right)>0$). Subsequently, the polymer chains propagate or crosslink with other polymer chains, leading to an increase in the curing degree ($\alpha_{2}\left(t_{2}>t_{1}\right)>\alpha_{1}$)).

Termination refers to the processes in which the reactive radical centers on polymer molecules, as well as radicals, are terminated either by reacting with a free radical or with a radical that is on a chain. Each termination reaction results in a “dead polymer chain” $P_{dead}$ or a “dead radical” $R_{dead}$, respectively. Termination occurs according to three mechanisms: radical combination, radical disproportionation or radical trapping [82]. Radical combination refers to the combination of the ends of two growing polymer chains (Equation (5)),

(5) $$P^{*}+P^{*}\underset{k_{tc}}{\to }P_{dead}\;,\;\;$$

or the combination of a growing end of a polymer chain with a free radical (Equation (6)):

(6) $$P^{*}+R^{*}\underset{k_{tc}}{\to }P_{dead\;}$$

Radical disproportionation refers to the removal of a hydrogen atom, forming an unsaturated group and leading to the formation of two dead polymer chains (Equation (7)):

(7) $$P^{*}+P^{*}\underset{k_{td}}{\to }P_{dead\;}.$$

Radical combination and radical disproportionation are also referred to as bimolecular termination. Termination by radical trapping, referred to as unimolecular termination, occurs when active radicals become trapped between immobile polymer chains [83].

The termination rate coefficient $k_{t}$ in the Equations (5)–(7) does not only depend on temperature and pressure, but on parameters such as polymer weight fraction, solvent viscosity, polymer-monomer-solvent interactions, chain length of the macro-radicals involved in the termination reaction, chain flexibility, chain entanglements and molecular weight distribution as well [84].

In the presence of oxygen, propagation and termination reactions are inhibited as oxygen in the reaction volume acts as a radical scavenger, i.e., it can react with radicals, and reduces the quantum yields of the initiating radicals [80]. Oxygen reduces the efficiency of initiation and generally leads to significant retardation or even inhibition of the polymerization [33]. For instance, Decker et al. [22] stated that very little consumption of the monomer occurred until most of the oxygen in the reaction volume was consumed by reaction with radicals. Furthermore, it has been shown experimentally that the concentration of dissolved oxygen in the reaction volume must be lowered by at least two orders of magnitude before polymerization begins, due to the high reactivity of oxygen with radicals [82]. The inhibition mechanism can be described as follows (Equation (8)):

(8) $$R^{*}+O_{2}\underset{k_{t,oxy}}{\to }R_{dead}\;\;\;\mathrm{or}\;\;\;P^{*}+O_{2}\underset{k_{t,oxy}}{\to }P_{dead}.$$

In order to avoid oxygen inhibition, Lovestead et al. [23] proposed to use a light source with different wavelengths. A lower wavelength, which can only penetrate a few microns deep into the sample, can be used to cure the top layer and limit/prevent any additional oxygen diffusion into the sample. Subsequently, a higher intensity wavelength can be used to cure the rest of the sample once the pre-dissolved oxygen is consumed. According to Andrzejewska et al. [33], acrylates are generally more susceptible to oxygen inhibition than methacrylates.

In the equations listed hereinabove, $k_{d}$, $k_{i}$*,* $k_{p}$*,* $k_{t}$, and $k_{t,oxy}$ are denoted as the respective rate constants for decomposition, initiation, propagation, termination and termination by oxygen inhibition. The propagation rate $k_{p}$ and the termination rate $k_{t}$ are not constant. Both rates contribute to auto-acceleration and auto-deceleration, the two main regimes of kinetic behavior during propagation [23,74]. In the course of the polymerization reaction, the physical state of the medium changes from a viscous liquid to a viscoelastic rubber (in some cases finally to glassy materials) causing drastic variations of the reactive species mobility [1]. Both behaviors, auto-acceleration and auto-deceleration, are governed by the mobility changes of radicals and unreacted double bonds as a result of the continuing polymerization and crosslinking [19].

**Figure 15:**
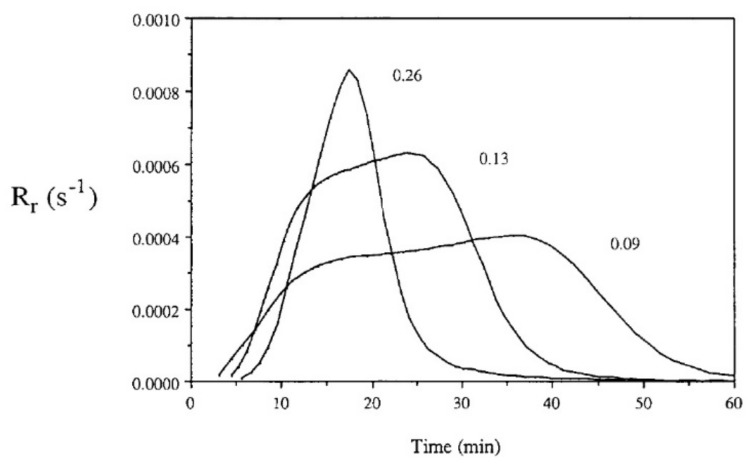
Polymerization rate $R_{r}$ versus time for various mixtures of vinyl ester resin and styrene with labels corresponding to the crosslinker concentration. Reprinted from reference [85] with permission of John Wiley and Sons.

Auto-acceleration (gel effect or Trommsdorff–Norrish effect) denotes a reduction in the termination kinetic constant $k_{t}$ and a significant increase in the polymerization rates due to increasing viscosity. A few seconds after irradiation, this effect, in which the segmental movement of radicals is restricted due to localized increases in the viscosity of the polymerizing system, can be observed [37]. Prior to that, chain termination by a combination of two free-radical chains occurs at a high frequency. However, when the concentration of “dead polymers” increases, i.e., the growing polymer molecules with active free-radical ends are surrounded by an increasingly viscous medium, the reduction in mobility and therefore hindered termination can be observed. The changes in viscosity affect the macromolecules but do not prevent smaller molecules, such as radicals and monomers, to move freely. Consequently, as termination collisions are restricted, the concentration of active polymerizing chains and the consumption of monomer rises rapidly, leading to a significant polymerization rate. According to Batch et al. [85], bimolecular termination is even more hindered in crosslinking polymerizations compared to linear polymerization. Consequently, for crosslinking systems, diffusion-limited termination occurs at even lower conversions compared to linear polymerization systems, and termination may be insignificant at this stage. Batch et al. [85] studied the influence of crosslinker concentrations on the polymerization rates using a vinyl ester resin mixed with styrene cured isothermally in DSC experiments at 60 °C. The experiments indicate that increasing the concentrations of crosslinkers increases both the initial slope of the polymerization rate $R_{r}$ and its maximum value $R_{r,\max}$ (Figure 15).

When the reaction continues, the system becomes even more viscous and is governed by a strong reduction in molecular mobility. The transition to a glassy state strongly affects the polymerization kinetics, reducing the mobility of the monomers and radicals. At this stage, the propagation reaction becomes diffusion-controlled leading to a decrease in the rate constant for propagation $k_{p}$, consequently leading to a decrease in the polymerization rate $R_{p}$. This decline in the polymerization rate is referred to as auto-deceleration or the glass effect [19].

**Figure 16:**
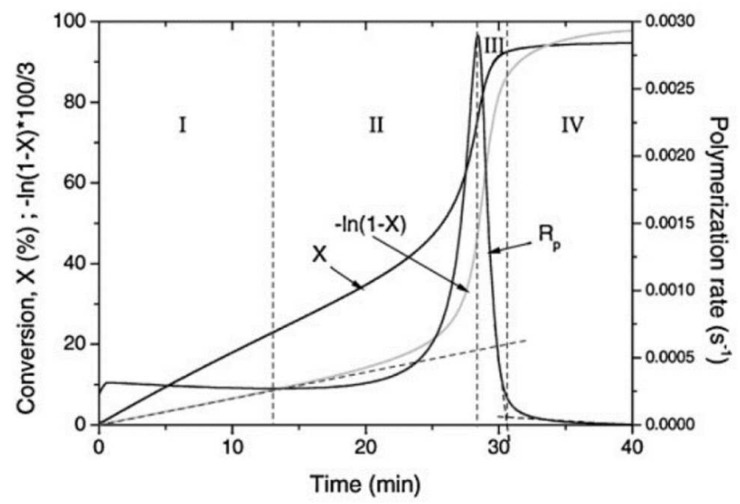
Indicative time evolution of the free-radical photopolymerization reaction (polymerization rate $R_{p}$, conversion X and $-ln\left(1-X\right)$ versus time) presenting the classification of the reaction into four regimes from polymerization of methyl methacrylate (MMA) at 80 °C with AIBN (azobisisbutyronitrile) 0.03 ${\mathrm{mol}\;L}^{-1}$. Reprinted from reference [84] with the permission of John Wiley and Sons.

The photopolymerization profile, also referred to as the conversion-time curve, has a characteristic S-shape and can be divided into four different regimes as for instance shown for the polymerization of methyl methacrylate (MMA) by Achilias [84] (Figure 16). At the very early stage after UV irradiation, the reactive species react with the inhibitors (e.g., oxygen in the case of free-radical photopolymerizations), leading to an induction period. When all the inhibitors are consumed, the reactive species react with the monomers yielding macroradicals that propagate. At this stage (Stage-I), the polymerization rate $R_{p}$ remains almost constant. The crossover of Stage-I and Stage-II denotes the onset of the gel effect. Therefore, Stage-II shows a sharp increase in the polymerization rate $R_{p}$, followed by an increase in the conversion X. The maximum polymerization rate occurs at the crossover of Stage-II and Stage-III. Stage-III is characterized by a significant decrease in the reaction rate $R_{p}$ (auto-deceleration). At very high conversions, $R_{p}$ tends asymptotically to zero. In this regime of polymerization, the polymerization reaction slows down and finally stops despite the continuing presence of both, radicals and monomer reactants.

#### 3.1.1. Mechanistic Models for Free-Radical Photopolymerizations

All mechanistic models have in common that they use first-order reaction equations to describe the concentration variations of the individual species, including photoinitiators, free radicals and monomers. However, they differ in how diffusion control is treated, i.e., by using propagation rate coefficients that decrease empirically with increasing conversion (e.g., Wu et al. [82]), or by applying the free volume concept (e.g., Bowman et al. [19], Anastasio et al. [86]).

In 2018, Wu et al. [82] proposed a spatial mechanistic kinetic model that employs first-order chemical reaction differential equations to calculate the variation of the species concentrations. Additionally, it includes the description of the spatial distribution of reactants inside the continuum body during the process. The monomer used in their work is poly(ethylene glycol) diacrylate (PEGDA) which is widely used in biomedical applications and 3D printing. In the model, the generation of free radicals due to photoinitiator reactions is considered according to Equation (9),

(9) $$\frac{\partial C_{I}\left(x,t\right)}{\partial t}=-\beta I\left(x,t\right)C_{I}\left(x,t\right)\;,$$

in which β denotes the decomposition rate.

The light intensity is the driving factor for the formation of free radicals. The Beer–Lambert law describes the light propagation through a homogeneous medium without internal sources or scattering and, therefore, the light intensity variation with changes in the spatial position of the sample. Wu et al. [82] used the three-dimensional version of the Beer–Lambert law, also referred to as the radiative-transfer equation [87] (Equation (10)):

(10) $$\Omega \left(x,t\right)\cdot \nabla I\left(x,t\right)=-A\left(x,t\right)I\left(x,t\right),$$

in which $\Omega \left(x,t\right)$ represents the direction of light propagation, $I\left(x,t\right)$ the light intensity at position x and time t, and $A\left(x,t\right)$ the local depletion rate of light intensity due to the absorbance of the species.

According to Wu et al. [82], the variation of the light intensity depends on the concentration of the light-absorbing species and the respective molar absorptivity. Therefore, the absorptivity is not simply that of the photoinitiators (as proposed for instance in a publication from Anastasio et al. [86]), but instead is a combination of photoinitiator, free radicals and polymer matrix which all can absorb photons and, hence, attenuate light as it propagates through the material. Consequently, Wu et al. [82] calculated the local depletion rate according to Equation (11),

(11) $$\;A\left(x,t\right)=\varepsilon C_{I}\left(x,t\right)+A_{absorber}\left(x,t\right)+\left[A_{polymer}p\left(x,t\right)+A_{monomer}\left(x,t\right)\left(1-\alpha \left(x,t\right)\right)\right]\;,$$

in which ε denotes the molar absorptivity of the initiator, $C_{I}\left(x,t\right)$ the concentration of the light-absorbing species, $A_{absorber}$ the absorption by photoabsorbers, $A_{monomer}$ the absorption by the unconverted monomer, $A_{polymer}$ the absorption by the converted polymer, and $\alpha \left(x,t\right)$ the degree of cure.

Light refraction is not considered in the model, as for photocuring systems light is usually irradiated in (or close to) the perpendicular direction [82]. Therefore, the light intensity in the thickness direction can be calculated according to Equation (12):

(12) $$\frac{\partial I\left(z,t\right)}{\partial z}=-A\left(z,t\right)C_{I}\left(z,t\right)I\left(z,t\right).$$

The evolution of radical concentration $C_{R}$ is modeled according to Equation (13),

(13) $$\frac{\partial C_{R}\left(x,t\right)}{\partial t}=m\beta I\left(x,t\right)C_{I}\left(x,t\right)-2k_{t}{\left(C_{R}\left(x,t\right)\right)}^{2}-k_{O}C_{R}\left(x,t\right)C_{O}\left(x,t\right)\;,$$

with the termination rate $k_{t}$, the concentration of radicals $C_{R}\left(x,t\right)$ (regardless of the chain length), the reaction rate $k_{O}$ between oxygen and radicals, and the concentration of oxygen $C_{O}\left(x,t\right)$. The parameter m denotes the number of radicals generated during photodecomposition, depending on the type of photoinitiator (e.g., two in the publication from Anastasio et al. [86]). The second term of Equation (12) is a termination term that accounts for the inactivation of radical species: if two active radicals react, they can recombine yielding a dead polymer, reducing the concentration of active radicals. Wu et al. [82] only considered a termination by radical combination but neglected chain length dependence, effects of polymer heterogeneity, and radical trapping as, for instance, proposed by Bowman et al. [19]. However, the authors stated that further termination mechanisms, e.g., monomolecular termination, could also be included in this model. The factor 2 that appears in the second term of the equation accounts for two radical chains being “inactivated” by the termination event. A reaction that further influences free-radical polymerizations is the inhibition by oxygen [88]. Due to the high reactivity of oxygen towards radicals, oxygen reacts very rapidly with the propagating radical, and the resulting peroxy radical is very unreactive towards propagation. This by-reaction, hence, represents one of the most relevant limitations of free-radical polymerization [19]. Therefore, the third term in the above equation is added to describe the evolution of oxygen in the solution $\frac{\partial C_{O}\left(x,t\right)}{\partial t}\;$.

The monomers in the solution are gradually consumed by combining with radicals, reducing the reactive functional groups. Therefore, the concentration of the unconverted functional groups can be modeled as shown in Equation (14),

(14) $$\frac{\partial C_{M}\left(x,t\right)}{\partial t}=-k_{p}C_{M}C_{R}\;,$$

in which $k_{p}$ denotes the propagation rate.

The curing degree does not explicitly appear in the system of differential equations, since it can only be evaluated once the degree of monomer conversion has been solved. The degree of cure can be calculated according to Equation (15),

(15) $$\alpha \left(x,t\right)=1-\frac{C_{M}\left(x,t\right)}{C_{M}\left(x,t=0\right)}\;,$$

in which $C_{M}\left(x,t\right)$ is the concentration of the monomer molecules (unconverted double bonds) at time t.

Although the volume shrinkage during the chemical reaction can increase the concentrations of different species, Wu et al. [82] observed that the effect of the volume concentrations was largely canceled in the equations for $C_{I}\left(x,t\right)$, $C_{R}\left(x,t\right)$, $C_{O}\left(x,t\right)$ and $C_{M}\left(x,t\right)$ and does not affect the curing degree noticeably. Consequently, the equations hereinabove use the concentration in the initial configuration. The presented approach by Wu et al. [82] was also taken up by Brighenti et al. [89] who developed a multiphysics modeling approach to predict the mechanical properties of polymers obtained via photo-induced polymerization.

**Figure 17:**
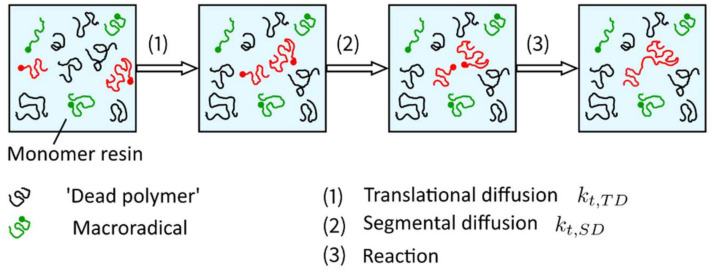
Scheme of bimolecular termination reactions between two macroradicals (colored in red). Two polymer coils must come into contact by center-of-mass translational diffusion (1), and segmental reorientation (segmental diffusion) (2) has to occur in order to bring both reactive chains ends in proximity and to form a radical-radical encounter pair (3).

One challenge associated with the model presented by Wu and his co-authors is the implementation of non-constant propagation and termination rates $k_{p}$ and $k_{t}$, as both parameters are a function of conversion (the molecular mobility in the reaction medium decreases as the polymerization proceeds) [19,37,86]. Effects such as auto-acceleration and auto-deceleration can only be accounted for when these coefficients are modeled as functions of conversion. In particular, $k_{t}$ is highly dependent on conversion [19]. According to Buback et al. [90], changes in the termination rate $k_{t}$ are caused by the following two dominant mechanisms for termination that occur in parallel: termination due to species translational diffusion $k_{t,D}$ and termination due to reaction-diffusion $k_{t,RD}$. Diffusion-controlled termination generally occurs when the mobility of the growing polymer chains is hindered and can be summarized in Equation (16),

(16) $$k_{t}=k_{t,D}+k_{t,RD},\;$$

in which the species translational diffusion $k_{t,D}$ is subdivided into the center of mass translational diffusion $k_{t,TD}$ and segmental diffusion $k_{t,SD}$. These two mechanisms of species translational diffusion occur consecutively, i.e., in order to react with each other, radicals must be close enough to meet (referred to as center-of-mass translational diffusion $k_{t,TD}$), and the reactive groups must be reoriented into proper position in order to react with each other (referred to as segmental diffusion $k_{t,SD}$) (Figure 17). According to Wu et al. [82], segmental diffusion $k_{t,SD}$ is often treated as a constant. The diffusion of the radical’s center of mass, described by the center of mass translational diffusion $k_{t,TD}$, depends on the viscosity of the solution [91]. Increasing viscosity leads to a decrease in the center of mass translational diffusion defined by Equation (17),

(17) $$k_{t,TD}=\frac{1}{\exp\left(c\alpha \right)},$$

with the relative viscosity coefficient c and the curing degree α [82].

**Figure 18:**
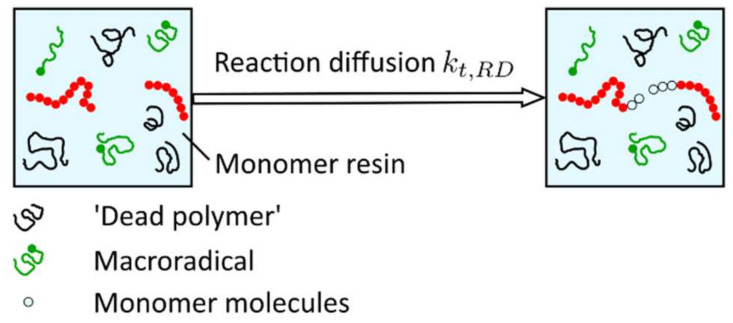
Scheme of reaction-diffusion where the movement of the growing radical site is attributed to the addition of monomer molecules at the chain end (propagation).

The idea behind reaction-diffusion, described with the parameter $k_{t,RD}$, is that the radical site at the end of a growing chain does not only move as a result of diffusive motion but also because chain growth occurs at this side, i.e., the radical end also moves when the polymer chain grows due to the addition of monomer molecules at the chain end (propagation) (Figure 18).

According to Bowman et al. [19], reaction-diffusion termination is a unique mobility mechanism for radicals in crosslinked systems. As a result of the classical diffusion mechanisms, the radical mobility drops to such an extent that their centers of mass are essentially immobile, and the only possible diffusive motion of radical chain ends becomes the propagation reaction [33]. The reaction-diffusion rate can be modeled to be proportional to the rate coefficient for propagation $k_{p}$ and the monomer concentration. If any effect of volume shrinkage is ignored, the monomer concentration is proportional to $\left(1-\alpha \right)$ [90], and the reaction-diffusion rate is defined according to Equation (18),

(18) $$k_{t,RD}=C_{RD}k_{p}\left(1-\alpha \right),$$

in which $C_{RD}$ represents the reaction-diffusion proportion parameter and $k_{p}$ the propagation rate modeled according to the publications of Buback et al. [90,92], as well as Dickey and Willson [93].

**Figure 19:**
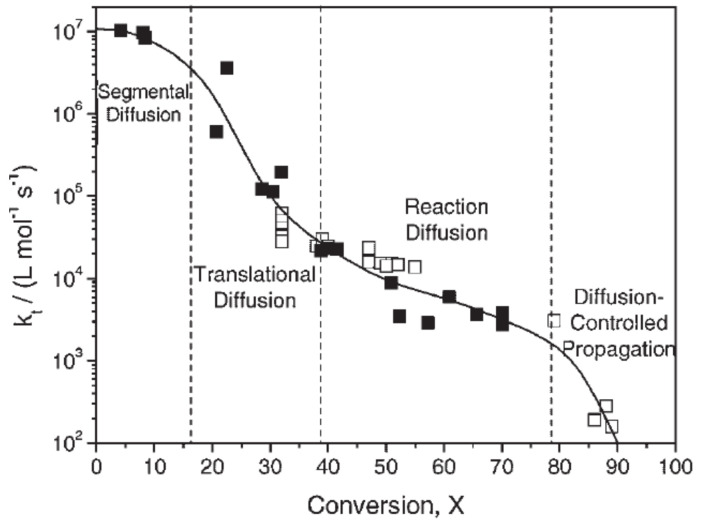
Termination rate coefficient $k_{t}$ vs. conversion for methyl methacrylate at 0 °C (▪) and 50 °C (▫). Reprinted from reference [84] with the permission of John Wiley and Sons.

Figure 19 exemplarily shows the variation of the termination rate coefficient as a function of conversion for the polymerization of methyl methacrylate published by Achillias et al. [84]. As long as the increase in segmental diffusion is counterbalanced by the decrease in translational diffusion, the termination rate coefficient $k_{t}$ remains constant or decreases moderately only with increasing conversion. The initial conversion range, in which the termination rate coefficient remains approximately constant, is considerably dependent on the monomer type [84]. At the point at which the center-of-mass (translational) diffusion becomes rate-determining, the termination rate constant decreases, leading to an increase in the total macroradical concentration and the polymerization rate (auto-acceleration). At higher conversions, the effect of auto-acceleration stops, and the termination-controlling mechanism changes to reaction-diffusion. The observed decrease in $k_{t}$ is gradual only. At even higher conversions, diffusion-controlled propagation is observed. Combining the equations hereinabove, the total termination rate used for the kinetic model proposed by Wu et al. [82] is defined according to Equation (19),

(19) $$k_{t}=\frac{1}{\frac{1}{k_{t,SD}}+\frac{\exp\left(c\alpha \right)}{k_{t,TD0}}}+\frac{C_{RD}\left(1-\alpha \right)k_{p0}}{1+\frac{k_{p0}}{k_{p,D0}}\exp\left(c\alpha \right)}\;,$$

where $k_{p0}$ denotes the polymerization rate at the beginning of the reaction $\left(\alpha =0\right)$, $k_{t,TD0}$ is the rate of mass translational diffusion at zero conversion, and $k_{p,D0}$ corresponds to a parameter used to characterize the diffusion-controlled propagation reaction.

**Figure 20:**
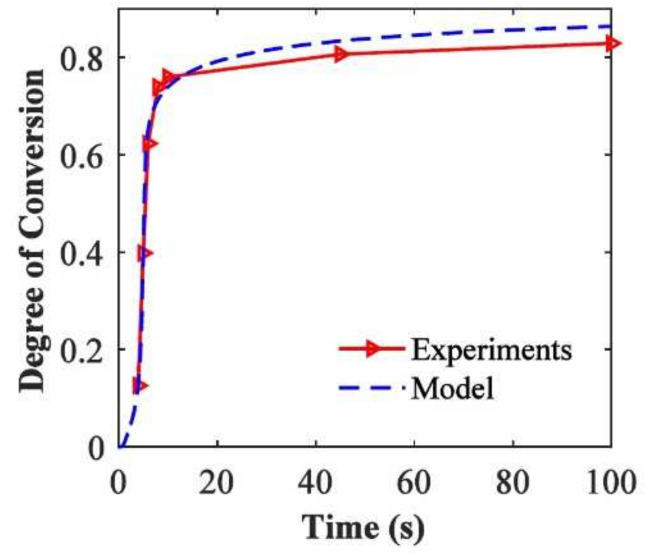
Degree of conversion versus reaction time for free-radical photopolymerization of PEGDA. Reprinted from reference [82] with permission of Elsevier.

The kinetic model by Wu et al. [82] was validated for PEGDA ($M_{n}$ = $250{\;g\;\mathrm{mol}}^{-1}$) with 0.3 wt.% of 2,2-dimethoxy-2-phenylacetophenone as photoinitiator. The samples were cured with a UV curing lamp with a 365 nm wavelength bandpass filter and a light intensity of $5{\;\mathrm{mW}\;\mathrm{cm}}^{-2}$ on the top surface of the solution. FTIR measurements were used to obtain the parameters used for the reaction kinetics model. Figure 20 shows the degree of conversion versus the reaction time for the experimental results and the kinetic model [82]. It reveals that the model results match the experimental conversion rate well and that the model accurately captures the auto-acceleration effect where the degree of cure increases rapidly after a conversion of about 12.0%.

Apart from modeling the photopolymerization kinetics in terms of the photopolymerization reaction mechanism, Wu et al. [82] also modeled the material property evolution, i.e., the evolution of the glass-transition temperature $T_{g}$ as well as the volume shrinkage during curing. The glass-transition temperature is an important indication of the curing extent, as it increases with fractional conversion. In order to describe the relationship between $T_{g}$ and the curing degree, Wu et al. [82] used the model proposed by Gan et al. [94], which considers the crosslinking effects on the mobility of the curing system and, consequently, also the significant changes above the glass-transition temperature at high curing degrees. Furthermore, it captures the evolution of $T_{g}$ of a wide variety of curing systems [82]. Consequently, the $T_{g}$ change during the curing process is modeled according to Equation (20),

(20) $$T_{g}=\frac{E_{r}}{Rln\left[g_{1}{\left(1-\alpha \right)}^{\xi }+g_{2}\right]}\;,$$

in which $E_{r}$ represents the activation energy for the transition from the glassy to the rubbery state, R is the gas constant, $g_{1}$ and $g_{2}$ are material constants, α is the curing degree, and ξ is a parameter accounting for the effects of chain entanglement.

Another spatially dependent polymerization model, which is also used for a broad range of industrial applications [88,95,96,97], was developed by Bowman et al. [19]. The model of Bowman and co-authors further includes chain-length dependent termination (CLDT), assuming that radicals diffuse and terminate according to their chain length [98]. According to Lovestead et al. [98], the kinetic chain length is affected by the initiation rate, i.e., with an increasing initiation rate, the kinetic chains become shorter. Shorter chains more readily diffuse and terminate easier according to their length. Consequently, the termination kinetic constant must incorporate all the different possible mechanisms that control termination: translational diffusion, segmental diffusion, reaction-diffusion and chain-length dependent termination. The model for incorporating chain-length dependent termination into the termination kinetic equations, proposed by Bowman et al. [19], builds on models that incorporate free volume theory and diffusion-controlled kinetics [38,95,96,99]. When the fractional free volume $v_{f}$ of the system is greater than the critical free volume $v_{cf}$, the polymerization is reaction limited. In the case that the fractional free volume $v_{f}$ of the system is less than the critical free volume $v_{cf}$, the polymerization is diffusion-controlled [95].

The termination kinetic constant incorporating chain-length dependent termination, described as a function of conversion, can be summarized in Equation (21),

(21) $$k_{t\;i,j}=k_{t0}^{1,1}{\left\{1+{\left[\frac{k_{t,\mathrm{RD}}k_{p}\left[M\right]}{k_{t0}^{1,1}}+\frac{1}{2}\left\{\frac{1}{i^{\gamma }}+\frac{1}{j^{\gamma }}\right\}e^{-A_{t}\left(\frac{1}{v_{f}}-\frac{1}{v_{f,cf}}\right)}\right]}^{-1}\right\}}^{-1}\;,$$

in which $k_{t0}^{1,1}$ is the termination kinetic constant between two radicals of length 1, $\left[M\right]$ the monomer concentration, γ an exponent that describes the relationship between mobility and termination, $A_{t}$ a constant that controls the onset and rate of auto-acceleration, $v_{f}$ the fractional free volume, and $v_{f,cf}$ the critical free volume for the regime in which termination becomes controlled by the active species’ segmental motion, i.e., the termination transitions to diffusion control.

The fractional free volume, considering only the case without excess free volume, is assumed to be a function of conversion as proposed by Bowman et al. [99]. According to Bowman et al. [19], the chain-length dependent termination kinetic constant $k_{t\;i,j}$ accounts for radicals of length i terminating with radicals of length j and is able to predict a region in which termination is dependent on the radical chain lengths. However, the model also does not consider a limiting radical chain length to determine if the radical is incorporated (“trapped”) in the gel and no longer capable of diffusion-limited termination [100].

A pointwise mechanistic model was proposed by Anastasio et al. [86] to describe the monomer conversion by using the kinetics of the photopolymerization reaction for a methacrylate resin. Again, the reaction scheme of free-radical photopolymerization, described earlier in this section, is presented as a set of differential equations, describing the evolution of species concentrations over time.

However, as opposed to the model suggested by Wu et al. [82], this model does not include the description of the spatial distribution of reactants inside the continuum body during the reaction process. In the set of differential Equations (22)–(26), $\left[In\right],\;\left[R^{*}\right],\;\left[M\right],\;\left[P^{*}\right]$ and $\left[P_{dead}\right]$ correspond to the concentrations of initiator, free radicals, monomer, growing polymer chains and dead polymer chains, respectively.

(22) $$\frac{d\left[In\right]}{dt}=-k_{d}\left[In\right]$$

(23) $$\frac{d\left[R^{*}\right]}{dt}=2fk_{d}\left[In\right]-k_{p}\left[M\right]\left[R^{*}\right]-k_{t}\left[P^{*}\right]\left[R^{*}\right]\;$$

(24) $$\frac{d\left[M\right]}{dt}=k_{p}\left[M\right]\left[R^{*}\right]-k_{p}\left[M\right]\left[P^{*}\right]\;$$

(25) $$\frac{d\left[P^{*}\right]}{dt}=k_{p}\left[M\right]\left[R^{*}\right]-k_{t}\left[P^{*}\right]\left[R^{*}\right]-2k_{t}{\left[P^{*}\right]}^{2}\;$$

(26) $$\frac{d\left[P_{dead}\right]}{dt}=k_{t}{\left[P^{*}\right]}^{2}+k_{t}\left[P^{*}\right]\left[R^{*}\right]\;$$

Again, like with the model proposed by Wu et al. [82], the curing degree does not explicitly appear in the system of differential equations as it can only be evaluated after the problem related to the monomer conversion has been solved using the relationship summarized in Equation (27),

(27) $$\alpha \left(t\right)=1-\frac{\left[M\left(t\right)\right]}{\left[M\left(t=0\right)\right]},$$

in which $\left[M\left(t\right)\right]$ denotes the concentration of the monomer molecules at the time t. In the equations hereinabove, f corresponds to the initiator efficiency, namely the fraction of radicals that initiate the growth of a polymer chain, while $k_{d},\;\;k_{p}$ and $k_{t}$ correspond to the reaction rate constants for decomposition, propagation and termination. In order to be able to solve the set of equations, these parameters must be determined.

The initiator efficiency decreases as a function of conversion due to the “caging effect” leading to the recombination of free radicals [84]. The caging effect depends on the amount of the initiator radicals that are entrapped in the system during the curing reaction. Entrapped initiator radicals are not likely to be available for participation in the curing reaction. Han et al. [76] pointed out that the caging effect might be significant in the curing reaction of unsaturated polyester resins due to the formation of a three-dimensional network structure as opposed to the polymerization of methyl methacrylate or styrene that yields linear (uncrosslinked) macromolecules.

In the model proposed by Anastasio et al. [86], the recombination (“trapping”) process ($R^{*}+R^{*}\underset{k_{t}}{\to }2R_{dead}$) is considered by reducing the initiator efficiency accordingly (Equation (28)):

(28) $$f=\frac{1}{1+\exp\left[C\left(\frac{1}{v_{f}}-\frac{1}{v_{f,cf}}\right)\right]}.$$

At the beginning of the reaction, the initiator efficiency is assumed to be 1. C is an adjustable parameter and represents the rate at which the initiator efficiency decreases with increasing conversion. In their implementation of the model, Anastasio et al. [86] used a value of C=1  for the adjustable parameter to study the predictive capabilities with respect to changing process conditions. However, Anseth and Bowman [38], for instance, use C as a fitting parameter. $v_{f}$ is the fractional free volume of the system and is modeled as a function of conversion as proposed by Bowman et al. [99]. $v_{f,cf}$ is the critical free volume at which propagation becomes diffusion controlled.

According to Anastasio et al. [86], a critical aspect of the proposed model for polymerization kinetics concerns the determination of the initiator decomposition rate $k_{d}$. The authors determine the initiator decomposition rate $k_{d}$ using a modified Beer–Lambert law for penetration of light into a medium, as suggested by Boddapati [80]. The initiator decomposition rate depends on the concentration of the initiator $\left[In\right]$, the incident intensity of the light source $I_{0}$ and depth into the absorbing medium  z according to Equation (29),

(29) $$k_{d}=2.3\phi \varepsilon I_{0}\cdot \exp\left(-2.3\varepsilon \left[In\right]z\right)\cdot \frac{\lambda }{N_{A}hc}\;,$$

in which ϕ represents the quantum yield of the initiator, ε the molar absorptivity of the initiator, λ the wavelength of light, h Planck’s constant, and c the speed of light.

Anastasio et al. [86] used termination reaction rates including the diffusion effects by using a limited number of adjustable parameters as proposed by Anseth and Bowman [38]. The model of Anseth and Bowman includes reaction-diffusion, the transition from reaction-controlled to diffusion-controlled reaction and volume relaxation and is in good agreement with experimental results [101].

The model described hereinabove neglects the translational diffusion because, according to Achillias [84], the translational diffusion of the polymer chains in a crosslinking system is negligible (already) from the start of the reaction. Using this approach, the termination rate constant is described by Equation (30),

(30) $$k_{t}=k_{t0}\left[1+\left(\frac{1}{R\left(\frac{k_{p}}{k_{p0}}\right)+\exp\left[-A\left(\frac{1}{v_{f}}-\frac{1}{v_{f,ct}}\right)\right]}\right)\right]\;,$$

with $k_{t0}$ as the initial termination rate constant, R a constant, $k_{p}$ the propagation rate constant, $k_{p0}$ the initial propagation rate constant, $v_{f}$ the fractional free volume of the system, and $v_{f,cf}$ the critical free volume at which propagation becomes diffusion controlled. Similar to C in Equation (8), A is an adjustable parameter and is used as a fitting parameter in the model of Anseth and Bowman [38]. In the implementation of the model proposed by Anastasio et al. [86], the parameter A is set to 1.

**Figure 21:**
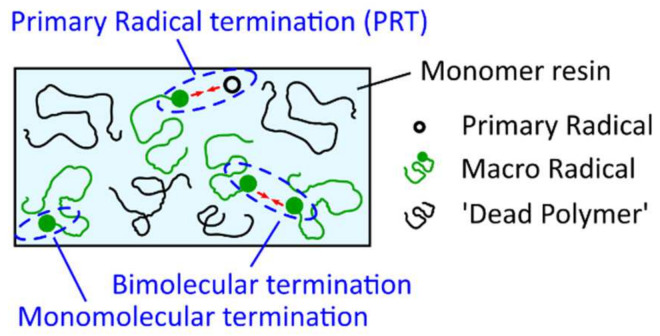
Termination mechanisms considered in the kinetic model of Christmann et al. [102].

A similar model, where the main kinetic rate constants are defined as functions of the fractional volume $v_{f}$ in order to consider their progressive diffusional control throughout the photopolymerization reaction was proposed by Christmann et al. [102]. Christmann states that most of the kinetic models to study the complex free radical photopolymerization mechanism and its related effects do not consider simultaneously all the termination pathways and/or neglect the evolution of terminations along the polymerization reaction. However, according to Ibrahim et al. [103], the proportion of the termination mechanisms that occur during free radical photopolymerization is expected to evolve during the polymerization because of the progressive increase in the medium viscosity which limits the species motion as the tridimensional network evolves. The termination mechanisms, considered in the model of Christmann et al. [102], namely biomolecular termination, primary radical termination, and monomolecular termination are shown in Figure 21. Bimolecular termination can either occur by a combination (formation of a chemical bond between two macroradicals) or disproportion (hydrogen abstraction from a macroradical to a second one with the formation of a double bond on the former). As combination and disproportion both involve a reaction between two macroradicals, they are lumped into a single termination mechanism [102]. Primary radical termination refers to the reaction between a primary radical and a macroradical. At final conversion, the polymer network is vitrified, and it can be assumed that all remaining macroradicals are trapped by occlusion in the polymer network (monomolecular termination).

The above-mentioned termination reactions are modeled by a set of differential equations (Equations (31)–(33)):

(31) $$\frac{d\left[bimol\right]}{dt}=2k_{t,b}{\left[R{\left(C=C\right)}_{n}^{*}\right]}^{2}.$$

(32) $$\frac{d\left[R{\left(C=C\right)}_{n}R_{A}\right]}{dt}=2k_{t,PRT}^{R_{A}^{*}}\;\left[R{\left(C=C\right)}_{n}^{*}\right]\left[R_{A}^{*}\right].$$

(33) $$\frac{d\left[R{\left(C=C\right)}_{n}R_{B}\right]}{dt}=2k_{t,PRT}^{R_{B}^{*}}\;\left[R{\left(C=C\right)}_{n}^{*}\right]\left[R_{B}^{*}\right].$$

In Equation (31), $\left[bimol\right]$ represents the concentration of macroradicals terminated by bimolecular termination, either diffusional or through reaction-diffusion. In Equations (32) and (33),$\left[R{\left(C=C\right)}_{n}R_{A}\right]$ and $\left[R{\left(C=C\right)}_{n}R_{B}\right]$ are the concentrations of macroradicals terminated by primary radical termination by $R_{A}^{*}$ and $R_{B}^{*}$, respectively. $R_{A}^{*}$ and $R_{B}^{*}$ denote the phosphonyl and benzoyl radicals which are yielded by dissociation of TPO under light exposure. In the publication of Christmann et al. [102], the main kinetic rate constants are defined as functions of the fractional free volume $v_{f}$ in order to consider their progressive diffusional control throughout the photopolymerization process. Christmann and his co-authors model the decreasing fraction of unoccupied volume in the reaction medium, the free volume $v_{f}$, according to Equation (34), as

(34) $$v_{f}=0.025+\alpha_{M}\left(T-T_{g,M}\right)\Phi_{M}+\alpha_{P}\left(T-T_{g,P}\right)\left(1-\Phi_{M}\right).$$

α is the thermal expansion coefficient, and $T_{g}$ is the glass transition temperature with the respective subscripts M (monomer) and P (polymer). $\Phi_{M}$ denotes the volume fraction of the monomer and is defined in Equation (35),

(35) $$\Phi_{M}=\frac{1-Conversion}{1-Conversion+Conversion\;\cdot \frac{\rho_{M}}{\rho_{P}}}\;,$$

where ρ corresponds to the volumetric mass density of the monomer (M) and the polymer (P). According to the authors, the propagation rate constant $k_{p}$ can be modeled using the following relationship (Equation (36))

(36) $$k_{p}=\frac{k_{p0}}{\left(1+\exp\left(A_{P}\left(\frac{1}{v_{f}}-\frac{1}{v_{f,cP}}\right)\right)\right)}\;.$$

**Figure 22:**
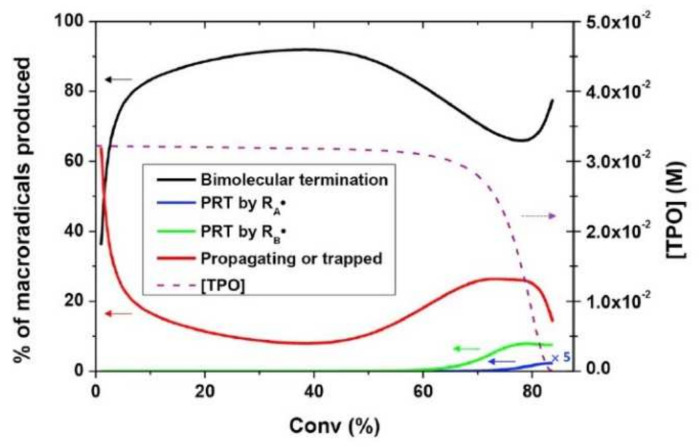
Evolution of the fractions of terminated species, propagating or trapped macroradicals (left scale) and TPO concentration (right scale) as a function of the acrylate conversion.

The above expression incorporates the propagation intrinsic rate constant $k_{p0}$ (i.e., without any diffusional control), a parameter $A_{p}$ which governs the rate at which $k_{p}$ decreases with viscosity, the free volume $v_{f}$, and the critical fractional free volume $v_{f,cp}$ at which propagation becomes diffusion-limited. The initiation rate constant $k_{i}$, as well as the rate constant for primary radical termination $k_{t,PRT}$ are modeled similarly to the propagation rate constant, see Equation (36). Christmann et al. [102] assume that the initiation rate constant $k_{i}$ and the rate constant for primary radical termination start decreasing at the same time and at the same rate as the propagation rate constant $k_{p}$. This implies that the respective exponential factors $A_{i}$ and $A_{t,PRT}$ and the critical fractional free volume $v_{f,ci}$ and $v_{f,ct,PRT}$ are assumed to be equal to $A_{p}$ and $v_{f,cp}$. The values of the intrinsic rate constants $k_{i,0}$ and $k_{t,PRT0}$ depend on the nature of the primary initiating radical. Diffusional bimolecular termination and subsequent reaction-diffusion processes are modeled using Equation (37),

(37) $$k_{t,b}=k_{t,b0}{\left(1+\frac{1}{\frac{R_{rd}k_{p}}{k_{t,b0}}+\exp\left(-A_{t,b}\left(\frac{1}{v_{f}}-\frac{1}{v_{f,ct,b}}\right)\right)}\right)}^{-1}\;,$$

where $k_{t,b0}$ corresponds to the intrinsic bimolecular termination rate constant (i.e., without any diffusional control), $R_{rd}$ is a constant, $A_{t,b}$ is an exponential factor that governs the rate at which $k_{t,b}$ decreases with viscosity, and $v_{f,ct,b}$ represents the critical free volume at which bimolecular termination becomes diffusion-limited. Christmann et al. [102] successfully applied the aforementioned kinetic model considering simultaneously all possible termination pathways (bimolecular termination, primary radical termination, and radical trapping by occlusion) to the photopolymerization initiated by a type-I photoinitiator (cleavage type, i.e., photoinitiators that dissociate into two radicals following photon absorption), showing a good agreement with experimental results. Furthermore, the authors were capable of identifying the relative contribution of the different termination pathways throughout the photopolymerization process. Christmann et al. [102] showed that bimolecular termination is the major termination reaction during the whole photopolymerization process. However, due to the progressive diffusion control of the polymerization reactions as the polymer network grows and due to the cessation of initiation when the photoinitiator is totally consumed, the ratio of bimolecular termination as well as of primary radical termination and macroradicals evolves. Figure 22 provides a deeper insight into the evolution of the termination modes during the photopolymerization reaction. The figure reveals that bimolecular termination is the main termination process. According to the authors, the strong growth of the bimolecular termination at the early stages of photopolymerization can be explained by the continuous production of macroradicals, as initiation occurs. Only in the last stages of the photopolymerization process does primary radical termination (PRT) become efficient. Christmann et al. [102] relate this to the competition between primary radical termination and initiation reactions for the primary radicals until the last stage of the photopolymerization reaction.

The model based on the free volume principle, presented by Christmann et al. [102], was also used by Gao et al. [104] to combine polymerization kinetics with reaction conditions to realize a 3D printing preview for stereolithography.

In 2017, Wang et al. [105] proposed a point-wise mechanistic model for modeling the photopolymerization reaction kinetics of Exposure Controlled Projection Lithography (ECPL). Oxygen diffusion effects, which were found to have a significant influence on the size, shape and properties of parts fabricated with stereolithography, are also incorporated in the model. The authors consider oxygen diffusivity in two dimensions as described in Equation (38),

(38) $$\frac{\partial \left[O_{2}\right]}{\partial t}=-k_{t,O_{2}}\left[R^{*}\right]\left[O_{2}\right]+D_{O_{2}}\frac{\partial^{2}\left[O_{2}\right]}{\partial x^{2}}+D_{O_{2}}\frac{\partial^{2}\left[O_{2}\right]}{\partial z^{2}}\;\;.$$

$k_{t,O_{2}}$ denotes the rate constant for termination of a radical with oxygen, $\left[R^{*}\right]$ denotes the concentration of free radicals, $\left[O_{2}\right]$ denotes the concentration of oxygen, and $D_{O_{2}}$ denotes the oxygen diffusion coefficient.

The models presented so far, and also models published, for instance, by Goodner et al. [96], Lee et al. [106], Buback et al. [90], Dickey et al. [93,107] and Long et al. [87] assume that the termination is due to radical recombination, which is the most common assumption for termination [108]. However, Wen et al. [109] state that a kinetic model that ignores radical trapping fails to predict two important aspects of experimental observations: Firstly, the concentration of trapped radicals increases monotonically with conversion, whereas the concentration of active radicals increases initially and then drops at high conversions [110]. Secondly, a higher light intensity leads to a lower fraction of trapped radicals at a given conversion but to a higher trapped radical concentration at the end of the reaction [110]. In order to include radical trapping, Wen et al. [109] extended the functional-group reaction scheme shown at the beginning of this section with the formation of trapped radicals like in Equation (39),

(39) $$R^{*}\underset{k_{b}}{\to }R_{b}^{*},\;$$

in which $R_{b}^{*}$ are trapped (buried) radicals and $k_{b}$ is the rate constant for radical trapping (burying). Radical trapping is assumed to take place according to a unimolecular first-order reaction. Consequently, the material balance equations proposed by Wen et al. [109] include the trapped radical concentration $\left[R_{b}^{*}\right]$ and the active radical concentration $\left[R^{*}\right]$, according to Equation (40),

(40) $$\frac{d\left[R_{b}^{*}\right]}{dt}=k_{b}\left[R^{*}\right].$$

**Figure 23:**
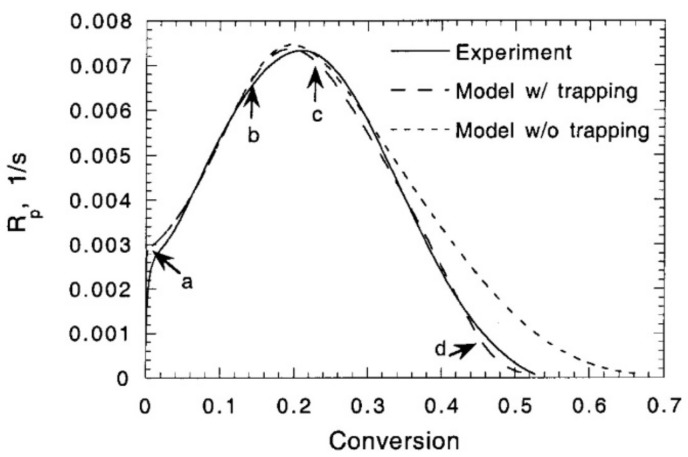
Predicted reaction rate (with and without radical trapping) and actual reaction rate measured by photo-DSC versus conversion during the polymerization of DEGDMA with 0.42 $\mathrm{mW}\;{\mathrm{cm}}^{-2}$ light intensity and 0.1 wt.% DMPA. Reprinted from reference [109] with permission from the American Chemical Society.

In the proposed model, the propagation rate constant $k_{p}$, the termination rate constant $k_{t}$, as well as the rate constant for radical trapping $k_{b}$, are simple functions of free volume following the model developed by Anseth and Bowman [38,101]. The model proposed by Wen et al. [109] presumes that the rate constant for radical trapping $k_{b}$ increases with conversion as radical trapping occurs more and more often as the chain growth in the course of the polymerization proceeds. Therefore, the rate constant $k_{b}$ is modeled to increase exponentially with the inverse of the fractional free volume $v_{f}$ according to Equation (41),

(41) $$k_{b}=k_{b0}\;\exp\left(\frac{A_{b}}{v_{f}}\right),$$

in which $k_{b0}$ is the pre-exponential factor and $A_{b}$ the dimensionless activation volume that governs the rate at which radical trapping increases as a function of fractional free volume. Wen et al. [109] compared their proposed model for predicting the reaction rate $R_{p}$ (with and without radical trapping) to photo-DSC experimental results during the polymerization of DEGDMA with 0.42 $\mathrm{mW}\;{\mathrm{cm}}^{-2}$ light intensity and 0.1 wt.% DMPA (Figure 23). The markers on the dashed curve for the model considering radical trapping show (a) the onset of auto-acceleration, (b) reaction-diffusion becoming dominant for termination, (c) radical trapping becoming dominant for termination, and (d) the propagation reaction becoming reaction-diffusion controlled. The figure shows that the model, including radical trapping, is consistent with experimental measurements of the polymerization rate $R_{p}$. The reaction rate before ~25% conversion is not severely affected by radical trapping. However, for conversions higher than 25%, the reaction rate appears to be higher without trapping, finally resulting in the prediction of a higher final conversion.

Another model including the termination by radical trapping was proposed by Batch and Macosko [85]. However, this model is rather limited as it requires the a priori knowledge of the final monomer concentration and the monomer concentration when trapping begins if it does not begin immediately. Perry et al. [108] suggested a model, building on the model introduced by Batch and Macosko [85] that circumvents the limitations mentioned above and that also includes dark reactions that occur in the course of the photopolymerization, i.e., the polymerization does not stop once the light source is extinguished, but reactions continue in the dark period. 

The models discussed hereinabove give accurate and useful predictions for isothermal systems. However, O’Brien and Bowman [97] stated that some polymerization systems were more complex and the inclusion of additional factors such as heat generation, heat transfer and mass transfer was necessary. The authors pointed out that in particular heat effects were important, as the kinetic constants, as well as the diffusion coefficients, are a function of temperature. Therefore, the one-dimensional kinetic photopolymerization model proposed by O’Brien and Bowman, which is based on the work of Goodner and Bowman [81,95,96], incorporates not only the temporal and spatial variation of species concentration, temperature and light intensity through the sample depth but additionally heat and mass transfer effects. As pointed out in the publication, additionally to the model proposed by Goodner and Bowman [95], this model is capable of simulating both photobleaching and non-photobleaching initiators.

In the kinetic model proposed by O’Brien and Bowman [97], mass transfer effects are considered by adding a diffusive flux term to the species balance as shown in Equation (42),

(42) $$\frac{dC_{i}}{dt}=R_{i}+\frac{d}{dz}\left(\hat{C}D_{i}\frac{d{\hat{x}}_{i}}{dz}\right),$$

in which the parameter $C_{i}$ denotes the concentration of species (while the index i corresponds to the respective component). The species balance is set up for initiator, primary radical, monomer, polymer radical, and dead polymer where the mobile species are the initiator, monomer and primary radicals. $R_{i}$ is the reaction term, i.e., the term for species generation or consumption by the reaction. The second term corresponds to the diffusive flux, including the effective total concentration of mobile species $\hat{C}$, the diffusion coefficient $D_{i}$, and the effective mole fraction of mobile species ${\hat{x}}_{i}$. The diffusion coefficient for the mobile species is calculated using the equation provided by Bueche [111]. The mathematical form of $\hat{C}$ and ${\hat{x}}_{i}$ can be found in the publication of Goodner and Bowman [95].

In addition to the species balance, the energy transport is incorporated in the model using an energy balance that includes heat transfer, as well as heat generation by radiation absorption and heat generation by reaction according to Equation (43):

(43) $$\rho c_{p}\frac{dT}{dt}=k\frac{d^{2}T}{dz^{2}}+\varepsilon_{I}I_{0}c_{I}^{*}-\Delta H\frac{d\left[C=C\right]}{dt},$$

in which $\rho c_{p}\frac{dT}{dt}$ represents the heat accumulation with the density ρ and the heat capacity $c_{p}$. The first term of Equation (36) represents the heat transfer, in which k is the thermal conductivity and z is the spatial coordinate for the sample depth. The second term of Equation (36) represents the heat generation by radiation and assumes that all energy absorbed by the system is converted into heat; $\varepsilon_{I}$ is the molar absorption coefficient of the initiator, $I_{0}$ the incident light intensity, and $c_{I}^{*}$ the concentration of all light-absorbing species. By adjusting the initiator molar absorption coefficient, varying optical densities can be simulated. The last term of Equation (36) corresponds to the heat generation by a reaction due to the exothermic nature of the polymerization reaction; ΔH denotes the heat of polymerization and $d\left[C=C\right]/dt$ the consumption of double bonds (monomer consumption).

Phenomena like diffusion-controlled kinetics and termination by reaction-diffusion are described in terms of the fractional free volume theory of the polymerizing mixture. Chain-length independent propagation, termination and inhibition are assumed. Furthermore, bimolecular termination is realized by considering a lumped termination rate constant $k_{t}$ that accounts for both combination and disproportionation. Physical and thermal properties such as density, specific heat and conductivity are assumed to remain constant in the course of the reaction. The attenuation of the curing light caused by the absorption of light by the initiator is modeled according to Beer–Lambert law (Equation (44)),

(44) $$I=I_{0}\;\exp\left(-\varepsilon_{I}c_{I}z\right),\;\;$$

where I denotes the light intensity at a depth z, and $c_{I}$ the unreacted initiator concentration. O’Brien and Bowman [97] adjusted the overall light absorbance, which determines the degree of attenuation, by varying one of the following parameters: initiator concentration, molar absorption coefficient and sample thickness. The authors pointed out that different combinations of these three parameters led to the same absorbance and therefore to the same initial light attenuation. However, in the course of the reaction, differences would be apparent as each of the three variables has distinct influences on other aspects of polymerization.

In order to solve the differential equations of the species and energy balances, O’Brien and Bowman [97] considered different thermal boundary conditions (insulating, conducting and constant temperature boundary conditions) affecting the heat transfer in a polymerizing sample. The inhibitory effect of oxygen on free-radical photopolymerization was incorporated into the model presented above by O’Brien and Bowman in 2006 [88].

Intensive research with regard to modeling the curing kinetics and deriving analytical relationships between curing depth and crosslink time, as well as considering the effects of oxygen inhibition and viscosity was conducted by Lin and his co-authors [30,112,113,114,115,116,117]. For instance, in 2016 Lin et al. [112] proposed a comprehensive mechanistic model for the cure kinetics of photopolymerization in optically thick polymers, providing useful guidance for the parameters selection and optimization for predicting the curing time for various polymer thicknesses in photoinitiated polymerization systems. In their publication, the authors state that most kinetic models presented in the literature are based on the oversimplified assumption that the photolysis product becomes completely transparent after polymerization. However, the distribution of the photoinitiator is non-uniform (depletion of the photoinitiator concentration) and the UV-light may still be absorbed by the photolysis product besides the absorption of the monomer [112]. Consequently, the authors derived kinetic equations for the concentration of the unreacted photoinitiator $C\left(z,t\right)$, see Equation (45), and the UV light intensity, see Equation (46).

(45) $$\frac{\partial C\left(z,t\right)}{\partial t}=-a\cdot I\left(z,t\right)\cdot C\left(z,t\right),\;$$

with $a=8.36\lambda \phi \varepsilon_{1}$, where λ is the light wavelength, ϕ is the quantum yield, and $\varepsilon_{1}$ is the molar extinction coefficient of the initiator.

(46) $$\frac{\partial I\left(z,t\right)}{\partial t}=-2.303\;\left[\left(\varepsilon_{1}-\varepsilon_{2}\right)\cdot C\left(z,t\right)+\varepsilon_{2}C_{0}F\left(z\right)+Q\right]\cdot I\left(z,t\right),\;$$

where $\varepsilon_{2}$ is the molar extinction coefficient of the photolysis product, $C_{0}$ is the initial concentration of the photoinitiator on the surface $C_{0}=C\left(z=0,t=0\right)$, $F\left(z\right)$ is a distribution function for the initial photoinitiator concentration in the polymer system, and Q is the absorption coefficient of the monomer and the polymer repeat unit. For the simplified case reported, for instance, by Ivanov et al. [118], $F\left(z\right)=1$, Q=0, and $\varepsilon_{2}=0$ applies. However, Lin et al. [112] report analytical equations for the general case of a non-uniform photoinitiator concentration $F\left(z\right)$ without the assumption of Q=0, and $\varepsilon_{2}=0$. The initial non-uniform photoinitiator concentration is given by the distribution function, see Equation (47),

(47) $$F\left(z\right)=1-\frac{0.5z}{D},\;$$

where D is the half-width at half-maximum [112]. When D is much larger than the polymer thickness $F\left(z\right)=1$ applies, corresponding to the flat distribution or uniform case. The equations of Lin and his co-authors also include a time-dependent generalized Beer–Lambert law, denoted as “Lin law”. According to the authors, the oversimplified assumption that the light intensity in the polymer follows a conventional Beer–Lambert law (with neglected depletion of photoinitiator concentration $C\left(z,t\right)$) is only valid for optically thin materials. The authors state that the depletion of the photoinitiator $C\left(z,t\right)$ will also affect the time-dependent profiles of the intensity $I\left(z,t\right)$ which, in general, will not follow the Beer–Lambert law. Therefore, the so-called “Lin law” has two modifications compared to the Beer–Lambert law: (1) it has a time-dependent term and (2) it has a z-dependent term accounting for the fact that the photoinitiator concentration $C\left(z,t\right)$ is an increasing function of z (for t>0), leading to a more accurate description for both $C\left(z,t\right)$ and $I\left(z,t\right)$.

Lin et al. [112] furthermore define the crosslinking time $T^{*}$ based on the time needed to deplete the photoinitiator concentration, i.e., the time needed for the completion of the gelation procedure. $T^{*}$ was found to be an exponentially increasing function of z and to be inversely proportional to the UV light intensity. Another important key parameter of photopolymerization, namely the local photoinitiation rate of production of free radicals was modeled according to Equation (48):

(48) $$R\left(z,t\right)=167.2\cdot \lambda \cdot \varepsilon_{1}\cdot \phi \cdot I\left(z,t\right)\cdot C\left(z,t\right),\;$$

According to the above equation, the photoinitiation rate is proportional to the product $\varepsilon_{1}\cdot C\left(z,t\right)\;$ and the light intensity $I\left(z,t\right)$, two competing factors. The authors derived an analytic equation for the optimal product $\varepsilon_{1}C_{0}^{*}$, expressed in Equation (49),

(49) $$\varepsilon_{1}C_{0}^{*}=\frac{1}{\left(1-\frac{\varepsilon_{2}}{\varepsilon_{1}}\right)z}\;\exp\left[a\left(I_{0}t\right)A\left(z\right)\right].\;$$

Consequently, the optimal photoinitiation rate is an increasing function of the initial light intensity $I_{0}$, the quantum yield ϕ, and the initiator absorption $\varepsilon_{1}$. Furthermore, the optimal photoinitiation rate is a decreasing function of the depth z, the initial photoinitiator concentration $C_{0}$, and the photolysis product absorption $\varepsilon_{2}$.

In 2019, Lin et al. [114] modeled the optimal conditions for maximum efficacy and crosslink depth, presenting analytic formulas for curing depth and crosslink time of photoinitiated polymerization, without the assumption of thin-film or spatial average. Furthermore, the authors derived kinetic equations for the efficacy and curing depth for systems with type-I radical-mediated and type-II oxygen-mediated pathways under the quasi-steady-state assumption and bimolecular termination [115].

More recently, Lin et al. [116] presented the theoretical modeling and kinetics of the red-light controlled oxygen inhibition for improved UV-light initiated monomer conversion based on a novel strategy presented by Childress et al. [119]. As mentioned earlier, free-radical photopolymerizations are particularly sensitive to oxygen inhibition, i.e., oxygen can react with primary radicals and thereby reduce the efficiency of photopolymerization by quenching the primary initiating and propagating radicals. Conventional strategies to reduce oxygen inhibition in photoinduced polymerizations include physical methods (e.g., working in an inert environment, use of multiple photoinitiators with different rates of initiation, …) and chemical mechanisms (e.g., additives that are insensitive to oxygen, …). In 2019, Childress et al. [119] reported a novel strategy for red-light-controlled oxygen inhibition. The authors used red-light to preirradiate the monomer, followed by the UV-light excitation of the photoinitiator in order to independently achieve photosensitization and photoinitiation via irradiation of the two distinct absorption bands. The technique of Childress et al. [119] allows us to partially or completely eliminate the induction time using red light.

The research of Lin et al. [30] also considers three-component photoinitiating systems (A/B/C) where the co-initiators/additives serve the regeneration of the photoinitiator A and the generation of extra radicals.

#### 3.1.2. Phenomenological Models for Free-Radical Photopolymerizations

Phenomenological models are set up to treat the conversion of monomers as the only variable to characterize the polymerization process and ignore the variations of other species [82]. Therefore, Equation (1) represents the reaction that describes the whole curing process.

The simplest and most common analytical form of the function of the degree of cure is given by Equation (50),

(50) $$f\left(\alpha \right)={\left(1-\alpha \right)}^{n},\;$$

in which n represents the reaction order and α the extent of the reaction [29]. Furthermore, the rate constant $K_{c}\left(T\right)$ in Equation (1) is assumed to follow the Arrhenius equation that can be expressed according to Equation (51),

(51) $$K_{c}\left(T\right)=K_{0}\exp\left(\frac{-E_{a}}{RT_{abs}}\right)\;,$$

with the so-called pre-exponential factor $K_{0}$, the activation energy $E_{a}$, the universal gas constant R, and the absolute temperature $T_{abs}$.

Replacing $K_{c}\left(T\right)$ and $f\left(\alpha \right)$ in Equation (1) with the correlations summarized in Equations (38) and (39), the so-called *n*-th order kinetic model [74] according to Equation (52) is obtained:

(52) $$\frac{d\alpha }{dt}=K_{0}\exp\left(\frac{-E_{a}}{RT_{abs}}\right){\left(1-\alpha \right)}^{n}\;.$$

For an isothermal reaction, the *n*-th order kinetic reaction model predicts a maximum of the reaction rate at the start of the reaction (t=0). Obviously, this model cannot be applied for photopolymerization reactions showing a maximum value of the reaction rate at any point, rather than the reaction starting point [120]. An alternative to the *n*-th order kinetic model is the so-called autocatalytic model proposed by Kamal et al. [120,121]. The general model equation of the autocatalytic model is given by Equation (53),

(53) $$\frac{d\alpha }{dt}=k\alpha^{m}{\left(\alpha_{max}-\alpha \right)}^{n},$$

in which α is the relative conversion, k an Arrhenius-type rate constant, m the autocatalytic exponent, n the reaction order exponent, and $\alpha_{max}$ the maximum conversion. $\alpha_{max}$ is considered to be 1 assuming the completed reaction. The applicability of the autocatalytic model in modeling free-radical photopolymerizations (as well as cationic photopolymerizations) is due to the fact that an auto-catalyzed reaction assumes a propagation reaction that is characterized by an accelerating conversion rate, with its maximum occurring well after conversion initiation [122]. This implies that for systems according to this auto-catalytic model, the reaction rate is initially equal to zero, and a maximum value of the reaction rate occurs at intermediate conversion. According to Achilias [84], the autocatalytic model of Kamal [120] is often used to describe diffusion-controlled polymerization reactions. For instance, the autocatalytic model has been used to describe the photoinitiated polymerization of dental resin-monomers and composite systems as well as multifunctional acrylates [33,46,123,124,125,126]. However, as mentioned earlier, this model is essentially phenomenological and does not provide any mechanistic insight.

A phenomenological model for the isothermal kinetic behavior of an acrylic resin accounting for the effect of auto-acceleration, vitrification and light intensity on the reaction kinetics has been proposed by Maffezzoli et al. [127]. The authors used the autocatalytic relation introduced by Kamal [120] to describe the conversion state with Arrhenius and power-law relationships for temperature and light intensity dependence. The kinetic behavior is modeled using a simple pseudo-autocatalytic expression summarized in Equation (54),

(54) $$\frac{d\alpha }{dt}=K\left(I_{a},T\right)\alpha^{m}{\left(\alpha_{max}-\alpha \right)}^{n}\left(1-\alpha \right),$$

in which K is a rate constant characterized by an Arrhenius-type dependence on temperature T and the absorbed light intensity $I_{a}$, m and n are positive fitting parameters independent of temperature, and $\alpha_{max}$ is the maximum degree of reaction obtained in isothermal DSC cure experiments. $\alpha_{max}$ is calculated as the ratio of the heat developed during the experiments ($Q_{isothermal}$) and the maximum heat of reaction measured in a non-isothermal experiment ($Q_{total}$). In their publication, Maffezzoli et al. [127] assumed that laser exposure (wavelength $\lambda^{*}$) led to an absorbed light intensity according to Equation (55),

(55) $$I_{a}=I_{0}\left(\lambda^{*}\right)\left(1-10^{-\varepsilon \left(\lambda^{*}\right)\left[In\right]z}\right),$$

in which ε denotes the molar absorbance of the photoinitiator depending on the wavelength of the light source, $\left[In\right]$ the initiator concentration, and z the thickness of the sample. The rate constant is consequently modeled according to Equation (56)

(56) $$K=K_{0}\left(T\right)I_{0}^{b},$$

containing the Arrhenius type temperature-dependent kinetic constant $K_{0}\left(T\right)$ and the fitting parameter b.

The considerations of Kamal et al. [120], as well as Maffezzoli and colleagues [127], were advanced by Rehbein et al. [128] for modeling the crosslinking reaction of photopolymers used in additive manufacturing processes such as digital light processing and stereolithography. The model of Rehbein et al. [128] describes the maximum attainable curing degree as a function of absolute temperature and light intensity. The model considers both, isothermal and non-isothermal processes, as well as time-dependent light intensity boundary conditions. Similar to the model proposed by Maffezzoli et al. [127], the model of Rehbein considers not only time-dependent light intensity but also the fact that the light intensity depends on the vertical location $z\left(t\right)$ since it evolves during the printing process. Therefore, the curing degree is modeled as an autocatalytic model of $\left(m+n\right)$-th order according to Equation (57)

(57) $$\frac{d\alpha }{dt}=\left[k_{1}\left(I\left(z\left(t\right),t\right),T\right)+k_{2}\left(I\left(z\left(t\right),t\right),T\right)\alpha^{m}\right]\cdot {\left(\alpha_{max}-\alpha \right)}^{n},$$

involving the location- and time-dependent light intensity $I\left(z\left(t\right),t\right)$.

In 2005, Bartolo [75] proposed a coupled photothermal phenomenological kinetic model to describe photoinitiated curing reactions and to correctly model the physical and chemical changes occurring in the bulk and in the surroundings of the material exposed to UV light, as well as the rates at which these changes occur. One main feature of the model is the modeling of the heat flow within the curing process, assuming that the irradiated material volume absorbs energy and initiates the phase change in the material (transformation from liquid to solid).

The temperature field in the region exposed to UV radiation can be described by the equation of heat conduction in a general three-dimensional framework according to Equation (58),

(58) $$\rho c_{p}\frac{\partial T}{\partial t}=\nabla \cdot \left(\lambda \nabla T\right)+q_{g},$$

containing the density ρ, the specific heat $c_{p}$, the thermal conductivity λ, the temperature gradient ∇T, and the volumetric heat generation $q_{g}$. It is assumed that the internal heat generation $q_{g}$ is influenced only by the heat of polymerization according to Equation (59),

(59) $$q_{g}=\rho H\frac{d\alpha }{dt}\;,$$

in which H represents the total heat release and dα/dt corresponds to the kinetic model. Furthermore, appropriate boundary conditions regarding temperature, heat flux emitted from the laser and convectional heat loss are assumed (for detailed information, see [129]).

For this model, the light intensity values at the resin surface were defined by assuming a Gaussian intensity distribution. The absorption of UV radiation is defined by the Beer–Lambert law and, consequently, the variation of the light intensity along the thickness of the resin layer (decrease in light intensity with depth) can be modeled according to Equation (60),

(60) $$I\left(s,z,t\right)=I_{0}\exp\left[-2{\left(\frac{s\left(t\right)}{w_{0}}\right)}^{2}\right]\exp\left(-\varepsilon \left[In\right]z\right),$$

where $s\left(t\right)$ represents the position in time of a point under irradiation, *z* the penetration depth (z=0 on the resin surface), $I_{0}$ the peak light intensity, $w_{0}$ the laser beam radius, ε the absorptivity of the layer, and $\left[In\right]$ the initiator concentration.

The coupled photothermal phenomenological kinetic model by Bartolo [75] consequently describes the evolution of the degree of cure according to Equation (61),

(61) $$\frac{d\alpha }{dt}=\frac{1}{1+\exp[\xi \left(\alpha -\alpha_{d)}\right]}\varphi I^{p}\exp\left(\frac{-E}{RT_{abs}}\right){\left[In\right]}^{q}\alpha^{m}{\left(1-\alpha \right)}^{n},$$

in which ξ is the diffusion constant, $\alpha_{d}$ the critical value of the curing degree corresponding to the onset of diffusion-controlled effects over the curing reaction, φ the pre-exponential factor of the rate constant, I the light intensity, E the activation energy, R the gas constant, and $T_{abs}$ the absolute temperature. The parameters p and q are constants. The exponents m and n denote the reaction orders; consequently, m+n is the overall reaction order. The kinetic parameters $\xi ,\;\alpha_{d},\;E$ as well as the exponents m,n are assumed to be non-constant but to vary in a non-linear way with temperature, light intensity and initiator concentration [129].

Bartolo et al. [75] also modeled the glass-transition temperature and suggested a non-linear relationship between the glass-transition temperature $T_{g}$ and the curing degree according to Equation (62),

(62) $$T_{g}=T_{g0}-T_{g0}\alpha +T_{g\infty }\alpha^{3},$$

with the glass-transition temperature $T_{g0}$ of the uncured polymer and the glass-transition temperature $T_{g\infty }$ of the fully-cured polymer. As crosslinking increases, the glass transition temperature increases due to the restriction of chain movements, associated with a decrease in the free volume during the curing process.

In 2019, Yang et al. [130] proposed a phenomenological kinetic model where the degree of cure is put in relation to the mechanical properties of laser-based additively manufactured components. The authors presented a mathematical model to quantify the tensile strength and hardness of stereolithography fabricated materials by estimating the solidification level. The authors assume that a specified layer i can be cured by the UV light more than once since printed layers can still be slightly targeted by the light that penetrates through the new fresh layer. Therefore, the printed layer is assumed to be re-cured when it is inside the photosensitive liquid resin implying that all printed layers are continuously cured. The phenomenological expression for the degree of cure for a specific layer i when it is solidified for the jth time is shown in Equation (63):

(63) $$\alpha_{i}^{j}\left(d,\theta \right)=t_{ci}^{j}\left\{S_{i}^{j}{}^{q}\;I_{i}^{j}{}^{p}\;\exp\left(\frac{-E}{RT_{i}^{j}\left(d,\theta \right)}\right)\;\alpha_{i}^{j}{\left(d,\theta \right)}^{m}\;{\left[1-\alpha_{i}^{j}\left(d,\theta \right)\right]}^{n}\right\}.$$

$t_{ci}^{j}$ corresponds to the curing time for the i-th layer when it is being cured for the j-th time, d denotes the thickness of the layer, θ is the stratification angle between surface normal vector and build direction, $S_{i}^{j}$ is the photoinitiator concentration (which is assumed to decrease in reverse proportion with the curing degree), $T_{i}^{j}$ is the temperature of for the i-th layer when it is being cured for the j-th time, and p,q,m,n are model parameters (e.g., related to environmental conditions, type of resin, a.s.o.) which are determined by best fitting of the experimental results. The above expression is similar to the phenomenological model derived by Bartolo et al. [75], see Equation (62), however, diffusion-controlled effects are neglected in the model proposed by Yang et al. [130].

In the literature, several modifications of kinetic models are proposed to express the diffusion limitations of reacting polymer chains with phenomenological models. A very early approach by Kenny et al. [131] incorporated the diffusion-rate control into the reaction-kinetic expression by using the maximum degree of conversion $\alpha_{m}$ achieved by isothermal curing. However, the authors assumed a linear relationship between the maximum degree of conversion and the cure temperature $T_{cure}$, which led to an infinite value of $\alpha_{m}$ with increasing temperature. Park et al. [132] derived a phenomenological *n*-th order kinetic model that incorporates the diffusion-rate control into the reaction-kinetic expression by using the maximum degree of conversion achieved in isothermal curing, expressed in Equation (64),

(64) $$\frac{d\alpha }{dt}=k\left(T\right){\left(1-\frac{\alpha }{\alpha_{m}\left(T\right)}\right)}^{n},$$

where $k\left(T\right)$ is the rate constant, α is the degree of cure, $\alpha_{m}$ is the maximum degree of cure achieved by isothermal curing and n is the reaction order. The maximum degree of cure as a function of isothermal cure temperature is expressed by an empirical equation (Equation (65))

(65) $$\alpha_{m}\left(T\right)=\frac{a}{1+b\;\cdot \;\exp\left(-k_{m}T\right)\;},$$

where the values of the fitting parameters a, b, and $k_{m}$ are obtained by a curve fitting method.

A similar approach, using a simple autocatalytic expression, was presented in 2017 by Kim et al. [133], see Equation (66),

(66) $$\frac{d\alpha }{dt}=k\;{\left(\frac{\alpha }{\alpha_{m}\left(T\right)}\right)}^{m}{\left(1-\frac{\alpha }{\alpha_{m}\left(T\right)}\right)}^{n},$$

expressing the maximum degree of cure by using an empirical equation in the form of Hill functions (Equation (67))

(67) $$\alpha_{m}\left(T\right)=a\frac{a^{c}}{b^{c}+a^{c}\;}.$$

Again, the values of the fitting parameters a,b, and c are obtained by a curve fitting method.

### 3.2. Cationic Photopolymerizations

UV-curable resins that are cured according to cationic photopolymerization mechanisms behave differently than those cured according to free-radical mechanisms. In cationic photopolymerization formulations, different photoinitiators and monomeric materials are used. Cationic polymerization reactions start with the initiation step, during which active centers are produced. The photoinitiation step is the only step in cationic photopolymerization that depends on light. Once the active centers are produced, they propagate by polymerization reactions without any further interaction with light. 

Cationic photoinitiators are referred to as photoacid generators PAGs. Once a cationic photoinitiator absorbs UV irradiation, the initiator molecule is converted into a superacid that initiates the polymerization. In the so-called propagation step, the active center reacts successively with a number of monomers such that they are covalently attached to the growing polymer chain. The polymer chain length is determined by the number of propagation steps that occur before the active center undergoes chain-transfer or termination. Compared to free-radical systems, cationic systems exhibit a relatively long lifetime and relatively small termination rates [134].

#### 3.2.1. Mechanistic Models for Cationic Photopolymerizations

A mechanistic mathematical model to characterize cationic photopolymerization kinetics of epoxy compounds as a function of temperature and exposure time has been proposed by Nelson et al. [134,135]. The authors modeled the rate of change of the active center concentration $\left[M^{+}\right]$ as a combination of the rate of active center generation by photosensitization (first term) and the rate of consumption by termination (second term) according to Equation (68),

(68) $$\frac{d\left[M^{+}\right]}{dt}=k_{i}\left[A\right]\left[I\right]-k_{t}\left[M^{+}\right],$$

in which $k_{i}$ represents the initiation rate constant accounting for a number of photophysical steps including excitation, intersystem crossing, exciplex formation and electron transfer [136], $\left[A\right]$ the concentration of photosensitizer, $\left[I\right]$ the initiator concentration, and $k_{t}$ the termination rate constant.

The model suggested by Nelson et al. [134] assumes that one active center is produced per photosensitizer and initiator molecule. Furthermore, all reactive centers are capable of propagating. Integrating the rate of change of the active center concentration, including the initial condition ${\left[M^{+}\right]}_{0}=0$ and assuming an exponentially decreasing photosensitizer concentration, Equation (69) is obtained:

(69) $$\left[M^{+}\right]={\left[A\right]}_{0}\frac{k_{i}\left[A\right]\left[I\right]}{k_{t}-k_{i}\left[A\right]\left[I\right]}\left(\exp\left(-k_{i}\left[A\right]\left[I\right]t\right)-\exp\left(-k_{t}t\right)\right).$$

Based on this mathematical model, Corcione et al. [28,137] proposed a temperature- and intensity-dependent mechanistic model for the photopolymerization kinetics of an epoxy-based resin for stereolithography, in which the rate of monomer consumption is expressed according to Equation (70),

(70) $$\frac{d\left[M\right]}{dt}=-k_{p}\left[M\right]\left[M^{+}\right],$$

in which $k_{p}$ denotes the propagation rate constant, $\left[M^{+}\right]$ the active center concentration, and $\left[M\right]$ the unreacted monomer concentration. The unreacted monomer concentration can be expressed as a function of the curing degree α and the initial monomer concentration ${\left[M\right]}_{0}$ according to Equation (71):

(71) $$\left[M\right]={\left[M\right]}_{0}\left(1-\alpha \right).$$

Consequently, the rate of conversion depends on the reaction time and the light intensity according to Equation (72):

(72) $$\frac{d\alpha }{dt}={\left[A\right]}_{0}\frac{k_{p}k_{i}\left[A\right]\left[I\right]}{k_{t}-k_{i}\left[A\right]\left[I\right]}\left(\exp\left(-k_{i}\left[A\right]\left[I\right]t\right)-\exp\left(-k_{t}t\right)\right)\left(1-\alpha \right).$$

Another mechanistic model was proposed by Pantiru et al. [138] for cyclic acetals using several cationic photoinitiators. In this model, the rate of monomer consumption is modeled according to Equation (73),

(73) $$-\frac{d\left[M\right]}{dt}=k_{p}\left[I^{+}\right]\cdot \left(\left[M\right]-{\left[M\right]}_{e}\right),$$

with the rate constant for propagation $k_{p}$, the concentration of active species $\left[I^{+}\right]$, the monomer concentration at time *t* $\left[M\right]$ and the equilibrium concentration ${\left[M\right]}_{e}$.

#### 3.2.2. Phenomenological Models for Cationic Photopolymerization

Similar to free-radical photopolymerizations, the simplest phenomenological model for cationic photopolymerizations is the *n*-th order reaction model (Equation (51)). Auto-catalyzed reaction models are generally described by the relation shown in Equation (51).

In 2002, van Assche et al. [139] proposed to quantify the effects of diffusion and consequently mobility restrictions on the cure kinetics with direct estimation of a so-called diffusion factor $DF\left(\alpha ,T\right)$. The diffusion factor is defined as the ratio between the experimentally measured conversion rate and the predicted conversion rate at the same reaction conversion, but without mobility restrictions according to Equation (74):

(74) $$DF\left(\alpha ,T\right)=\frac{{\left(\frac{d\alpha }{dt}\left(\alpha ,T\right)\right)}_{measured}}{{\left(\frac{d\alpha }{dt}\left(\alpha ,T\right)\right)}_{kin\_model}}.$$

The measured conversion rate is proportional to the heat flow measured in DSC, α denotes the conversion and T denotes the absolute temperature. Applying the autocatalytic model, proposed by Kamal [120,140], the predicted conversion rate is modeled according to Equation (75),

(75) $${\left(\frac{d\alpha }{dt}\left(\alpha ,T\right)\right)}_{kin\_model}=\left(k_{1}+k_{2}\alpha^{m}\right){\left(1-\alpha \right)}^{n}=k_{kinetic}\left(\alpha ,T\right){\left(1-\alpha \right)}^{n},$$

in which $k_{kinetic}\left(\alpha ,T\right)$ corresponds to the phenomenological rate constant. Combining the equations listed hereinabove yields an apparent rate constant $k_{apparent}\left(\alpha ,T\right)$ that includes the effect of diffusion on the phenomenological rate constant $k_{kinetic}\left(\alpha ,T\right)$ according to Equation (76),

(76) $${\left(\frac{d\alpha }{dt}\left(\alpha ,T\right)\right)}_{measured}=k_{apparent}\left(\alpha ,T\right){\left(1-\alpha \right)}^{n},$$

with $k_{apparent}\left(\alpha ,T\right)=DF\left(\alpha ,T\right)k_{kinetic}\left(\alpha ,T\right)$.

The apparent rate is quantified using the equation proposed by Rabinowitch [141] (Equation (77)):

(77) $$\frac{1}{k_{apparent}}=\frac{1}{k_{Diff}}+\frac{1}{k_{kinetic}}.\;$$

In order to calculate the temperature dependence of the diffusion-controlled rate constant, a Williams–Lendel–Ferry (WLF) equation [142], as proposed by Wisanrakkit et al. [143] and summarized in Equation (78) can be used,

(78) $$k_{Diff}=k_{Diff0}\left(T\right)\exp\left(\frac{C_{1}\left(T-T_{g}\left(\alpha \right)\right)}{C_{2}+\left(T-T_{g}\left(\alpha \right)\right)}\right),$$

in which $k_{Diff0}\left(T\right)$ is a constant related to local conditions for the creation of chemical bonds, T the absolute temperature, $T_{g}$ the glass-transition temperature, and $C_{1},C_{2}$ are constants depending on the epoxy system. $k_{Diff0}\left(T\right)$ is a constant employing an Arrhenius temperature dependency. Consequently, van Assche et al. [139] modeled the diffusion rate constant according to Equation (79),

(79) $$ln\left(k_{Diff}\left(\alpha ,T\right)\right)=ln\left(A_{D}\right)-\frac{E_{D}}{RT}+\frac{C_{1}\left(T-T_{g}\left(\alpha \right)\right)}{C_{2}+\left(T-T_{g}\left(\alpha \right)\right)}\;,$$

in which $A_{D}$ denotes the pre-exponential factor and $E_{D}$ the activation energy for the Arrhenius-dependent diffusion rate constant $k_{Diff0}\left(T\right)$.

**Figure 24:**
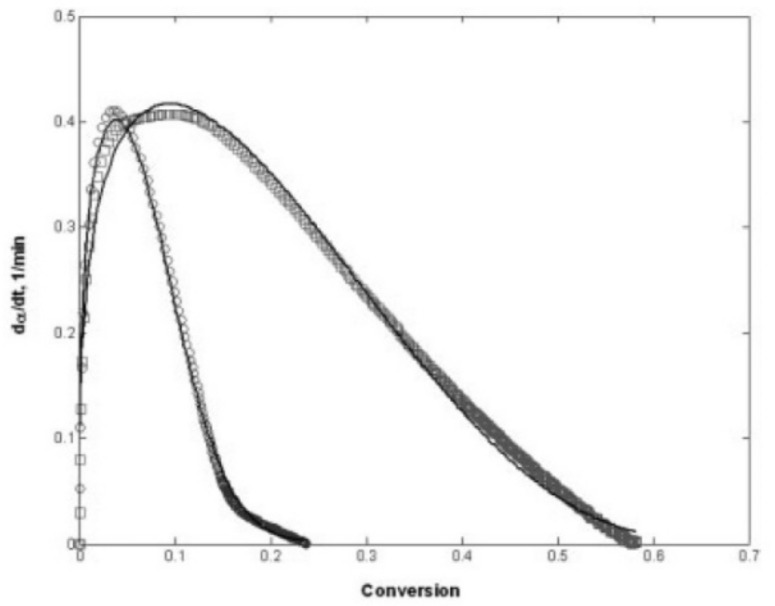
Comparison of experimental data and autocatalytic model of the cationic photopolymerization of ECH for different temperatures: Experimental data at 30 °C (◦), experimental data at 70 °C (▫), and prediction of the autocatalytic model (solid line). Reprinted from reference [29] with the permission of John Wiley and Sons.

Kim et al. [29] used the same approach for considering diffusion-controlled reactions in the phenomenological kinetic model. The experimental data and the prediction of the autocatalytic model for the photopolymerization of ECH (epichlorohydrin) resin with 1% of diaryliodonium hexafluoroantimonate (photoinitiator) showed a good agreement over the entire conversion range (Figure 24). The kinetic parameters of the autocatalytic model were obtained by non-linear regression analysis. The plot shows the polymerization rate versus conversion at different temperatures: the experimental data at 30 °C are shown as circles, the experimental data at 70 °C are shown as squares, and the model predictions are shown as solid lines, respectively.

The autocatalytic model shown in Equation (58) was also successfully used for the modeling of the cationic photopolymerization of cycloaliphatic diepoxide (CADE) systems with different photosensitizer concentrations [144]. Harikrishna et al. [122] also used the autocatalytic model for the cationic photopolymerization of 1,4-cyclohexane dimethanol diglycidyl ether. However, in this case, the kinetic parameters were studied by Levenberg-Marquardt [145,146] by a non-linear regression method instead of a conventional linear method in order to obtain more accurate values of the apparent rate constant. Further examples of modeling cationic photopolymerizations based on the autocatalytic model include publications from Boey et al. [147], Abadie et al. [148], and Macan et al. [149]. However, the dependency of the rate constant on the light intensity is neglected in all of these models.

In 2012, Golaz et al. [150] investigated the polymerization kinetics for the cationic photopolymerization of common difunctional cycloaliphatic epoxy monomers initiated by the decomposition of diaryliodonium salt photoinitiators and an isopropyl thioxanthone photosensitizer. The authors used an autocatalytic expression proposed by Sesták et al. [151] according to Equation (80),

(80) $${\left(\frac{d\alpha }{dt}\right)}_{t,T,I}=k\left(T,I\right){\left(\frac{\alpha }{\alpha_{max}}\right)}^{m}{\left(1-\frac{\alpha }{\alpha_{max}}\right)}^{n},$$

in which $k\left(T,I\right)$ is the rate constant, m the autocatalytic exponent, n the reaction order, α the degree of conversion, and $\alpha_{max}$ the maximum conversion.

The rate constant is dependent on the temperature T and light intensity I, represented by Equation (81),

(81) $$k\left(T,I\right)=k_{0}\left(T\right)I^{\beta },$$

with the temperature-dependent kinetics constant $k_{0}\left(T\right)$ following the Arrhenius equation and the exponent β as a fitting parameter.

The transmitted light intensity I is assumed to vary with the path length z following the Beer–Lambert law according to Equation (82),

(82) $$I\left(\lambda ,z\right)=I_{0}\left(\lambda \right)\exp\left(-\mu z\right),$$

in which $I_{0}$ denotes the incident light intensity depending on the wavelength λ and μ the attenuation coefficient.

**Figure 25:**
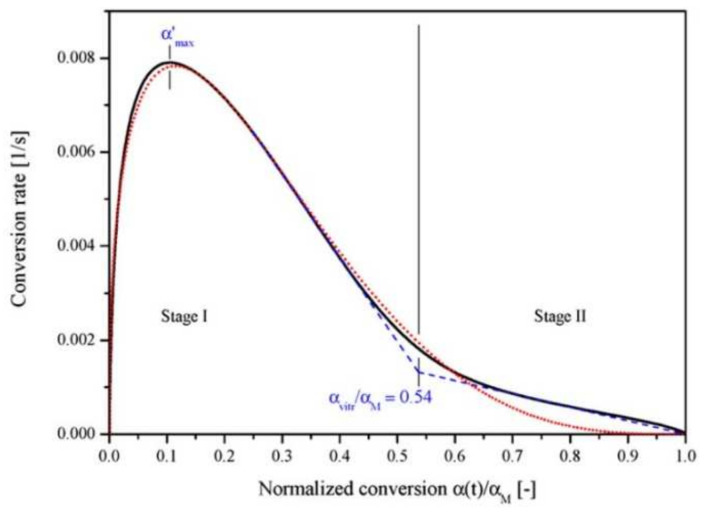
Comparison of experimental photo-DSC (black line) and modeled (red line) conversion rates versus normalized conversion of a cycloaliphatic epoxy compound at 30 °C and a light intensity of 50 ${\mathrm{mW}\;\mathrm{cm}}^{-2}$. Reprinted from reference [150] with permission of Elsevier.

Figure 25 shows the experimental photo-DSC and modeled conversion rates versus the normalized conversion of a cycloaliphatic epoxy compound at a temperature of 30 °C and a light intensity of $50\;{\mathrm{mW}\;\mathrm{cm}}^{-2}$. The conversion is normalized with the final (maximum) conversion, depending on temperature and intensity. The figure reveals that it was possible to predict the conversion rate and the conversion with reasonable accuracy up to vitrification applying Equation (81). However, after vitrification, the polymerization was faster than predicted by the autocatalytic model, confirming observations by Corcione et al. [137] about the excess of “free volume”.

The autocatalytic expression proposed by Sesták et al. [151] was also successfully applied to the kinetic modeling of bifunctional epoxy monomers by Voytekunas et al. [152]. However, as opposed to the model suggested by Golaz et al. [150], in this case, the rate coefficient $k\left(T\right)$ is assumed to depend on temperature and photoinitiator concentration.

Another phenomenological approach, presented by Jiang et al. [153], studied the reaction kinetics of the photopolymer using the Avrami theory of phase change for isothermal phase transfer as already applied for describing the cure kinetics of epoxies for instance by Xu et al. [154] or Chen et al. [155]. The Avrami theory in its general form is represented by Equation (83),

(83) $$\alpha \left(t\right)=1-\exp\left(-K\cdot t^{n}\right),$$

in which $\alpha \left(t\right)$ represents the reaction time-dependent curing degree, K the reaction speed constant, and n the reaction order. The Avrami theory was originally used to describe the kinetic process of polymer crystallization [156]. However, Pollard et al. [157] argued that it was possible to predict the curing process of thermosets using the Avrami equation as crystallization can be considered as a physical form of crosslinking. In order to accurately present the photocuring kinetics, Jiang et al. [153] replaced the reaction speed constant K, as well as the reaction order n, with undetermined coefficients a and b that depend on the light intensity I according to Equation (84),

(84) $$\alpha \left(t,I\right)=1-\exp\left(-a\cdot t^{b}\right),$$

for which a and b can be calculated according to Equation (85):

(85) $$a\left(I\right)=c\cdot I+d\;,\;b\left(I\right)=e\cdot I+f\;.$$

The undetermined constants c,d,e, and *f*, therefore, the undetermined coefficient kinetic parameters a and b were determined by linearly fitting isothermal photo-DSC experiments at different light intensities.

## 4. Implementation of Photopolymerization in Numerical Simulations

According to Marschik et al. [158] numerical methods can be used to effectively derive approximate solutions to models for which analytical solutions are not available. Numerical procedures are capable of handling large equation systems with different degrees of nonlinearities. Therefore, numerical simulations provide a useful tool to study and experiment with the complex interactions within photopolymerization reactions. Numerical implementation of photopolymerization reactions can yield valuable insight into which variables are more important than others and how these variables interact, e.g., exposure time and intensity of radiation. Therefore, some approaches regarding the implementation of photopolymerization models by numerical simulations are shown in this section.

As already presented in Section 3.1.2., Bartolo [75] proposed a coupled photothermal phenomenological kinetic model for free-radical photopolymerizations. In this model, the law of conservation of energy, describing the heat transfer phenomena, is coupled with an advanced kinetic expression that describes the cure kinetics in detail. The authors assume that the temperature field in the exposed region (laser light source) can be described by the two-dimensional heat conduction equation in cylindrical coordinates according to Equation (86),

(86) $$\rho c_{p}\frac{\partial T}{\partial t}=\frac{1}{r}\frac{\partial }{\partial r}\left(rk_{r}\frac{\partial T}{\partial r}\right)+\frac{\partial }{\partial z}\left(k_{z}\frac{\partial T}{\partial z}\right)+\rho H\frac{d\alpha }{dt},$$

**Figure 26:**
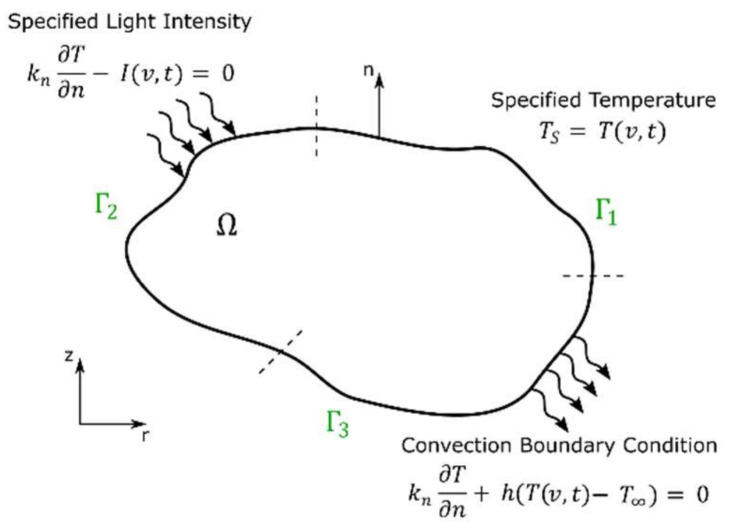
Schematic representation of the thermal-kinetic boundary conditions considered for the different regions $\Gamma_{1},\;\Gamma_{2},\;\Gamma_{3}$ of the domain Ω. Redrawn from reference [75].

The above equation assumes a simplified isotropic/anisotropic material with density ρ, specific heat $c_{p}$, thermal conductivities $k_{r}$ and $k_{z}$, and a total heat release H. Furthermore, T represents the temperature, t the time, and dαdt the kinetic model. The internal heat generation, expressed by $\rho H\frac{d\alpha }{dt}$ is only due to the heat of polymerization. The solution of the heat conduction equation requires the knowledge of initial conditions (i.e., initial temperature $T_{i}$ and initial value of the fractional conversion $\alpha_{i}$) and must satisfy specific boundary conditions (e.g., light intensity and convection). Bartolo [75] introduced the initial temperature $T_{i}$, according to Equation (87),

(87) $$T\left(v,0\right)=T_{i},\;$$

as well as the initial value of the fractional conversion $\alpha_{i}$, according to Equation (88),

(88) $$\alpha \left(v,0\right)=\alpha_{i}$$

in the domain being studied, where v represents a generic point in space. The thermal kinetic boundary conditions, namely a specified temperature, a specified light intensity, and a convection boundary condition are illustrated schematically in Figure 26 and given in Equations (89)–(91).

The boundary conditions proposed in the publication include a specified temperature $T_{s}$, according to Equation (89),

(89) $$T_{s}=T\left(v,t\right)\;\mathrm{at}\;\Gamma_{1},$$

a specified light intensity, according to Equation (91),

(90) $$k_{n}\frac{\partial T}{\partial n}-I\left(v,t\right)=0\;\;\;\mathrm{at}\;\;\;\Gamma_{2},$$

as well as a convection boundary condition, according to Equation (92),

(91) $$k_{n}\frac{\partial T}{\partial n}+h\left(T\left(v,t\right)-T_{\infty }\right)=0\;\;\;\mathrm{at}\;\;\;\Gamma_{3}.$$

$T\left(v,t\right)$ is the temperature at the generic point v in space at time t, ∂T/∂n is the derivative of the temperature in the direction normal to the surface, I is the light intensity, h is the coefficient of heat transfer, and $T_{\infty }$ is the temperature of the surrounding space.

The decrease in light intensity with depth is assumed to obey the Beer–Lambert law. Furthermore, any optical scattering effects and the flow of material due to convection or diffusion are both considered negligible. For the computer implementation of the thermal-kinetic model and the numerical solution of the heat conduction equation, subject to initial conditions and boundary conditions, two stages of approximation are involved: spatial approximation and temporal approximation. The generic domain Ω is discretized into an appropriate number of linear rectangular finite elements $\Omega_{ei}$. Bartolo [75] uses the Galerkin method, which is the most common weighted residual method to transfer the heat conduction equation to a form suitable for Finite Element Analysis (FEA) and to rewrite the heat conduction equation at the element level. The two-dimensional heat conduction equation can then be written in matrix form as shown in Equation (92):

(92) $$C\dot{T}+KT=F,$$

where C is the heat capacity matrix, K is the global “stiffness” matrix (also called the conductivity matrix) and F is the equivalent nodal heat flow vector. In practice, the above matrices are established for each element $\Omega_{ei}$ separately, and then assembled to give the global matrices. The global matrices can then be solved for the nodal temperatures by any numerical solution technique. To integrate Equation (93) with respect to time, the Crank–Nicholson method is used. According to the Crank–Nicholson algorithm, which is a finite difference method to numerically solve the heat conduction equation and similar partial differential equations, the unknown values of the temperature at the time $t_{n+1}$ can be determined through the known temperatures at the time $t_{n}$ considering a temporal approximation shown in Equation (93):

(93) $$T_{n+1}=T_{n}+\frac{1}{2}\;\Delta t\;\left({\dot{T}}_{n+1}+{\dot{T}}_{n}\right).$$

The fractional conversion, predicted by the kinetic model, is obtained with a fourth-order Runge–Kutta procedure. The computer implementation was organized in two levels: on the main level, developed in Visual Basic, all necessary input parameters were defined, whereas the routine level, developed in Fortran 77, represents the computational level. Flowcharts of the implemented code can be reviewed in the original publication [75]. From the simulations, Bartolo observed that the exothermic polymerization reaction and the irradiation process result in a temperature increase in the exposed region. As the reaction starts to slow down due to diffusion limitations, the temperature decreases due to conduction and convection dissipation effects until an equilibrium between the dissipated heat and the heat generated by irradiation is obtained after vitrification. Bartolo furthermore showed that the conversion typically decreases by increasing the distance from the light beam, due to the decrease in light intensity.

In 2020, Taki et al. [159] presented a simplified two-dimensional numerical simulation for the Continuous Liquid Interface Production (CLIP). The CLIP system is a 3D printing process, in which the liquid photopolymer resin is selectively exposed to UV light and is solidified into parts. The innovation of CLIP, which makes it unique from stereolithography applications (SLA) and digital light processing (DLP), is an oxygen-permeable membrane that creates a dead zone underneath the part which allows for continuous curing as the part is drawn out of the resin. 

**Figure 27:**
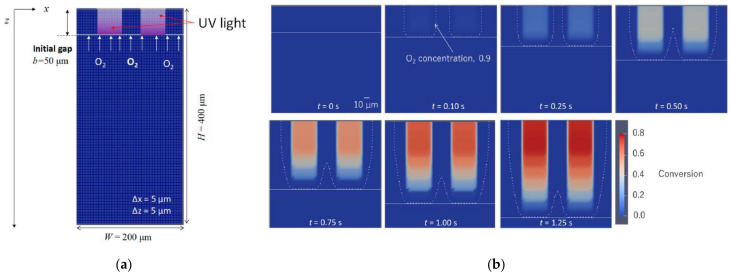
Simplified two-dimensional numerical simulation of photopolymerization reactions for the Continuous Liquid Interface Production (CLIP): (**a**) Geometry used for the numerical simulations; (**b**) Contour plot showing the results of the numerical simulations of C=C bond conversion as well as UV light position at a lift-up speed of 0.1 ${\mathrm{mm}\;s}^{-1}$ [159,160]. Reprinted from reference [159] with kind permission of the authors.

The aim of Takis’ approach was to simulate the shape of a printed object on a computer before printing the object in order to determine its final shape under influences such as volume shrinkage. The mechanistic model for free-radical photopolymerization, proposed in earlier publications by the same author [160,161], includes the photopolymerization kinetics of the initiation, propagation and termination (ordinal differential equations) as well as oxygen inhibition reactions (partial differential equations). The rate coefficient of propagation and termination was considered using the model of Anseth and Bowman [38]. The model neglects the effect of fluid flow induced by lifting the product, and a vertical movement of the light source was assumed instead of a realistic motion of the lifting of the photopolymerized parts. Furthermore, the temperature of the UV-curable resin was assumed to be constant, which implies another simplification. However, the author suggests that non-isothermal simulations of 3D printing in the CLIP system are subject to ongoing studies. Figure 27a shows the geometry of the numerical 2D simulation, as well as the areas subjected to UV exposure (colored in violet) as the UV light is moved downwards and is emitted upwards.

The boundary conditions for the numerical simulations include a periodic boundary condition on the concentration of oxygen in the horizontal direction, according to Equation (94), with the oxygen concentration $\left[O_{2}\right]$ depending on the spatial position z and the time t:

(94) $$\left[O_{2}\right]\left(0,z,t\right)=\left[O_{2}\right]\left(W,z,t\right).$$

The oxygen concentration at the UV light source was assumed to be constant and therefore equal to the initial oxygen concentration ${\left[O_{2}\right]}_{0}$ according to Equation (95):

(95) $$\left[O_{2}\right]\left(x,b,t\right)={\left[O_{2}\right]}_{0}.$$

Equation (96) furthermore considers that oxygen was not capable of diffusing away from the top, as expressed by

(96) $$\frac{\partial {\left[O\right]}_{2}}{\partial z}{|}_{z=0}=0.$$

**Figure 28:**
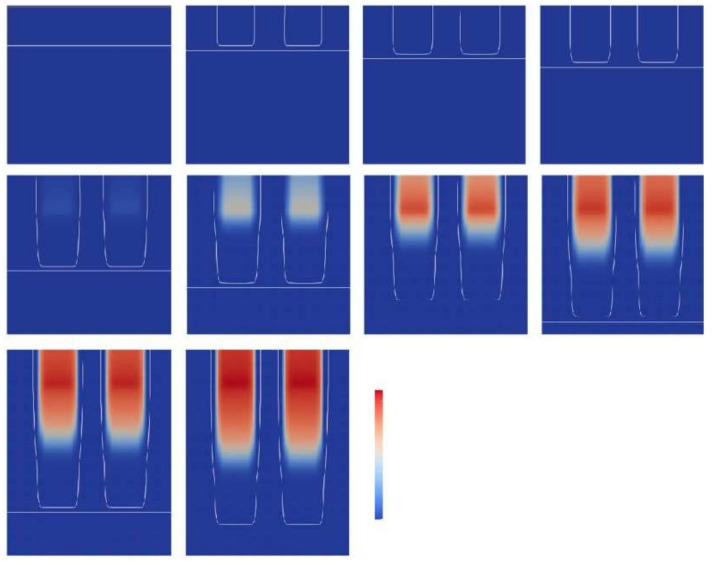
Contour plot showing the results of the numerical simulation of normalized oxygen concentration of 0.9 and C=C bond conversion distribution for a lift-up speed of 1 mm/s. Reprinted from reference [159] with kind permission of the authors.

Taki et al. [159] also imposed a boundary condition on the light intensity below the light source. Depending on the position x and z(z>b), the light intensity below the light source was assumed to be zero, according to Equation (97),

(97) $$E\left(x,z\right)=0,$$

with b as the vertical position of the light source. For z≤b the light was attenuated according to the Beer–Lambert law in z-direction expressed by Equation (98)

(98) $$E\left(x,z\right)=E_{0}\left(x\right)\cdot 10^{-\varepsilon \left[PI\right]\left(b-z\right)},$$

where $E_{0}\left(x\right)$ is the intensity of the UV light source depending on the position x, ε is the molar absorption coefficient, and $\left[PI\right]$ is the molar concentration of the photoinitiator. Despite the simplifications stated hereinabove, the numerical simulation showed the characteristic properties of a CLIP system, including the dead zone, in which the polymerization was inhibited due to radical quenching by oxygen. The numerical simulations were implemented using MATLAB. Figure 27b shows the contour plot of the numerical simulations of C=C double bond conversion. The horizontal white line represents the position of the UV light, which is moved downward at a speed of 0.1 ${\mathrm{mm}\;s}^{-1}$. At t=0.10 s, the oxygen concentration equaled 0.9 (shown as a dotted line) due to the fact that the initiator radical produced at t=0.0 s reacted with oxygen dissolved in the formulation. With increasing time, the C=C bond conversion starts to increase, indicated by the expansion of the interior of the dotted line and its coloring. However, a dead zone is clearly visible between the position of the light source and the C=C double bond conversion where polymerization does not occur since oxygen molecules quench the radicals. The size of the dead zone does not change with time due to the fact that the UV light intensity and oxygen permeation rate were assumed to be constant. As the lift-up speed of the light source is of major concern to the production speed, Taki et al. [159] also used a lift-up speed of 1 mm/s. The results shown in Figure 28 clearly reveal that the dead zone is expanded by an increase in the lift-up speed. As the maximum range of the color bar of Figure 28 is 50 times smaller than that in Figure 27b, the simulations also reveal that a faster lift-up speed leads to a decrease in the magnitude of the C=C bond conversion.

In 2018, Gao et al. [162] presented a multi-physics modeling approach based on a chemical kinetics model as well as a classical thermo-mechanical model to simulate the printing process of a Digital Light Projection (DLP) printer. The numerical model, proposed by the authors, aims at linking process conditions and material properties to understand how, for instance, exposure time and layer thickness, influence the mechanical properties of photopolymers and final products. The model, which is also suitable for modeling multi-layer products, can be used to determine the relation between the process conditions and the resulting properties of printed parts, for instance, warpage. The chemical (photopolymerization) kinetics model is based on the model of Goodner and Bowman [95]. Furthermore, Gao et al. [162] consider the influence of a dye on the light penetration depth $D_{p}$. Adding a dye slightly influences the formulation and allows for a higher controlled resolution in the layer thickness. The light absorption is modeled according to Beer–Lambert’s law, assuming a light intensity I, according to Equation (99),

(99) $$\frac{\partial I}{\partial z}=\frac{I}{D_{p}}\;if\;z\leq z_{0},$$

where the light direction is pointing to the z-direction and $z_{0}$ is the position of the surface where the light is projected.

As mentioned before, the light penetration depth depends on whether the resin is the only light absorber (see Equation (100)), or if a dye is added to the formulation (see Equation (101)):

(100) $$D_{p}=\frac{1}{\varepsilon_{PI}{\left[PI\right]}_{0}\ln10}\;$$

(101) $$D_{p}=\frac{1}{\varepsilon_{PI}{\left[PI\right]}_{0}\ln10+\varepsilon_{dye}{\left[dye\right]}_{0}\ln10}$$

where $\varepsilon_{PI}$ and $\varepsilon_{dye}$ denote the molar extinction coefficient of the photoinitiator and the dye, whereas ${\left[PI\right]}_{0}$ and ${\left[dye\right]}_{0}$ denote the initial molar concentration of the photoinitiator and the dye. The thermo-mechanical model considers the temperature increase due to the absorption of the UV light and due to the exothermic nature of the photopolymerization process (energy conversion). Two types of strains are considered: thermal expansion and chemical shrinkage. Chemical shrinkage due to conversion is assumed to be isotropic and linearly depending on the conversion. Consequently, chemical shrinkage can be expressed with Equation (102),

(102) $$\varepsilon^{chem}=-p\varepsilon_{max}^{chem},$$

where p is the double-bond conversion and $\varepsilon_{max}^{chem}$ is the maximum chemical shrinkage when the monomer is fully converted to the polymer. Furthermore, a mixture relation, shown in Equation (103), is introduced for the density ρ, the heat capacity $c_{p}$, the thermal conductivity κ, and the coefficient of thermal expansion α:

(103) $$\chi =p\cdot \chi_{poly}+\left(1-p\right)\cdot \chi_{mono}\;\;\mathrm{with}\;\chi =\rho ,\;c_{p},\;\kappa ,\;\alpha .$$

To capture the generated residual stress and to predict the warpage of the printed samples Gao et al. [162] introduced a plastic model by adding plasticity based on a purely elastic model. The plastic model assumes that the yielding stress of the material is linearly proportional to the conversion before a specified transition value $p_{tran}$ and becomes constant after that value. Empty material, representing a layer that is not printed yet, was realized with a Young’s modulus of $10^{-12}$ Pa and a thermal conductivity equal to zero. The printing process, involving the chemical reaction as well as the thermo-mechanical deformation, was simulated using COMSOL Multiphysics (Version 5.3a).

**Figure 29:**
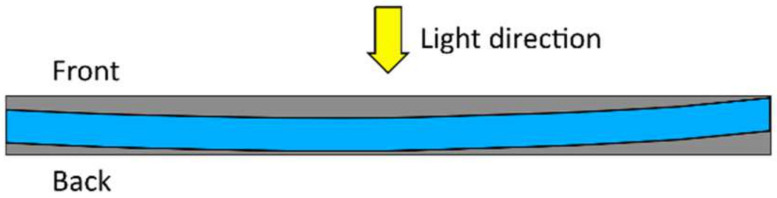
Schematic representation of the front surface and back surface of the beam, as well as the light direction. The beam shows a positive bending shape. Redrawn from reference [162].

The model developed by Gao et al. [162] was validated by comparing the conversion measured by FTIR with the predictions of the model. A schematic representation of the beam and the light direction is shown in Figure 29.

Gao et al. [162] found that the conversion measured for the back surface, independent of the exposure time, agreed very well with the predictions of the model. According to the authors, slight deviations might be attributed to the fact that the change in the light absorption capabilities of the photoinitiator and the polymer are not captured in the model.

The conversion on the front surface was found to increase with exposure time for both experimental measurements and model predictions. However, the conversion at the front surface showed significantly higher values compared to the predictions of the model. Gao et al. [162] provided some explanations for the deviations: for instance, free radicals trapped in the polymer chains cannot be consumed immediately, leading to a lower conversion measured in the experiments compared to the model predictions which do not consider trapped radicals. Furthermore, oxygen exposure of the front surface was not considered in the proposed model. Evaluating the warpage of the printed samples in terms of the deflection of the back surface revealed that a purely elastic model is not sufficient to describe the residual stress in the beam. The purely elastic model predicts a negative bending shape (i.e., a negative deflection) whereas a positive deflection is correctly predicted by the plastic model.

**Figure 30:**
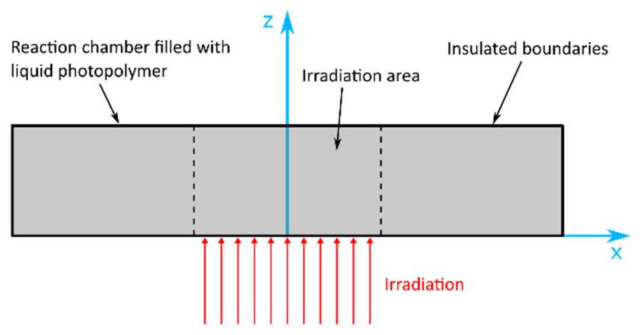
Reaction chamber modeled in COMSOL for the photopolymerization simulation of ECPL (Exposure Controlled Projection Lithography). Redrawn from reference [105].

As outlined in Section 3.1.1., Wang et al. [105] proposed a model for the photopolymerization reaction kinetics of Exposure Controlled Projection Lithography (ECPL). ECPL is an additive manufacturing process in which UV curing radiation, controlled by a dynamic mask is projected through a transparent substrate onto the photopolymer resin to fabricate three-dimensional structures. Compared to similar techniques to model the ECPL process, for instance, published by Mizukami et al. [163], Erdmann et al. [164], and Jariwala et al. [165], Wang et al. [105] present a more accurate, experimentally validated model with revised photopolymerization rates. The authors used the capabilities of COMSOL software to model the photopolymerization reaction kinetics for a two-dimensional finite element (FE) model, predicting the cured part geometry based on certain process parameters. Additionally, changes in the refractive index and degree of conversion were modeled throughout the reaction. A schematic sketch of the reaction chamber, modeled in COMSOL, aiming to predict the height and profile of the final cured part, based on the exposure time and intensity of the radiation, is shown in Figure 30. The rectangular reaction chamber is assumed to be filled with liquid resin. The red arrows indicate the regions into which irradiation is received by the monomer. All boundaries of the reaction chamber are assumed to be insulated, which, according to the authors, closely resembles the actual experiment conditions [105].

The polymerization kinetic model of free-radical photopolymerization of Wang et al. [105] incorporates the effect of oxygen inhibition and accounts for oxygen diffusion in two dimensions, as shown in Equation (104)

(104) $$\frac{\partial \left[O_{2}\right]}{\partial t}=-k_{t,O_{2}}\left[R\cdot \right]\left[O_{2}\right]+D_{O_{2}}\frac{\partial^{2}\left[O_{2}\right]}{\partial x^{2}}+D_{O_{2}}\frac{\partial^{2}\left[O_{2}\right]}{\partial z^{2}}.$$

To estimate the concentration of the individual species at a given time and location within the resin chamber (double bonds, live radicals, oxygen) the rate constants $k_{p}$ (for the propagation of a radical), $k_{t}$ (for termination between two radicals), $k_{t,O_{2}}$ (for termination of a radical with an oxygen molecule), and oxygen diffusion constant $D_{O_{2}}$ were modeled along with a diffusional model (chdi) in COMSOL. Compared to Jariwala et al. [165], who estimated the rate constants $k_{t}$, $k_{p}$, and $k_{t,O_{2}}$ by fitting the simulation results with the experimental data from Fourier-Transform Infrared (FTIR) experiments, Wang et al. [105] suggest, that the individual rate constants are not unique and may vary. Therefore, the authors provide revised values for $k_{t}$, $k_{p}$, and $k_{t,O_{2}}$.

In order to validate the ability of the reaction process model to accurately predict the geometric profile of the cured part, the COMSOL simulated profiles were compared to the experimental results for several samples at different exposure times (10 s, 20 s, and 30 s) with a UV light intensity of 8.86 ${W\;m}^{-2}$. The results, which can be reviewed in detail in the publication of Wang et al. [105], confirm that the process model is effective in predicting the geometry of the cured part. From the results, it is evident that the revised simulation prediction of the cured height agrees very well with the experimental data points, whereas the simulation before optimization of the rate constants predicts significantly higher cured heights. Furthermore, the results show that the cured height increases with increasing exposure time. Therefore, the simulation approach proposed by Wang et al. [105] is effective in predicting the geometry of the cured part. Similar simulations were also conducted by Jariwala et al. [166] who modeled the effects of oxygen inhibition and diffusion on the polymerization reaction in mask-based stereolithography for acrylate-based monomers.

## 5. Photopolymerization Composites

The curing of composite formulations in polymer photochemistry is particularly challenging due to the fact that the fillers in the formulations potentially lower the penetration depth of the UV light due to their opaqueness and/or their absorbance (see hereinabove). If inorganic fillers such as fiber reinforcing materials [167,168] and/or micro- and nanoparticles [169,170] have to be considered, the aspect of thermal conductivity also comes into play. While (unfilled) polymers commonly have low thermal conductivity in the range of 0.1–0.2 ${W\;m}^{-1}K^{-1}$ [171,172,173] (and seldomly in the range of up to 0.4 ${W\;m}^{-1}K^{-1}$ [174,175], inorganic fillers commonly have a significantly higher thermal conductivity [168,169,170]. Despite the fact that heat dissipation can potentially be considered to occur from composite formulations to a higher extent than from (unfilled) polymerization mixtures, the high relevance of composite materials shall be briefly highlighted in the example of three material classes:

### 5.1. Fiber-Reinforced Polymers 

In many cases, fiber-reinforced polymers show important properties such as wear resistance, high thermal stability, impact resistance, chemical resistance and high mechanical stiffness and strength [176,177]. A distinction can be made between two different types of fibers, (1) the synthetic fibers, which include glass fibers and carbon fibers, and (2) the natural fibers including cotton, hemp, jute, flax and others [178,179]. Due to the high mechanical load-bearing, glass fiber and carbon fibers are primarily used with an increase up to 113% in Young’s modulus when using 30 wt.% of carbon fibers [178]. 

### 5.2. Nanodielectrics

Nanodielectric composites combine the high dielectric permittivity of ceramic materials and the low loss factors, high dielectric strength (>300 kV/mm) and mechanical flexibility of polymeric materials [180]. As polymeric materials, polyethylene, polypropylene, (meth)acrylates and epoxides are used, into which mostly SiO_2_, ZnO, MgO, BaTiO_3_, Al_2_O_3_ or TiO_2_ nanoparticles are filled [181,182]. Qiao et al. [183] established a methacrylate composite with high permittivity (~20) and low tanδ (<0.02) over a wide range of frequencies (1 kHz to 1 MHz) using functionalized BaTiO_3_ nanoparticles. The permittivity was increased by 10 compared to the pure polymer, while the dissipation factor only increased by the minimum amount of 0.01 [183].

### 5.3. Electromagnetic Shielding Materials

Electromagnetic [EM] pollution caused through radio, cell phones, cellular networks and others, has become a worldwide problem due to its environmental and possible hazardous effects [184,185]. In order to prevent this, composite materials are used that either reflect or absorb the electromagnetic radiation. In principle, it can be distinguished between two absorption material classes. Materials with high dielectric constant, such as BaTiO_3_ and carbon particles, absorb the electric energy and convert it to thermal energy, whilst materials with high permeability, such as Fe_3_O_4_, absorb the magnetic energy and convert it to thermal energy [186]. Other materials, used for electromagnetic shielding, are metallic and magnetic materials such as steel, aluminum, copper, nickel, tin or carbon fibers [187]. Heat dissipation from polymerization mixtures is detrimental to frontal polymerizations (Section 6), as the heat generated in the course of the frontal polymerizations is the driving/initiating stimulus for the start of the polymerization reactions in areas in the vicinity of the polymerization front. If too much heat is dissipated, the polymerizations will stop, and the front will stop migrating.

## 6. Frontal Polymerization

Frontal polymerizations, in which the reaction process is mainly governed by the chemical and physical properties of the reacting system, is a promising curing strategy that substantially helps to reduce manufacturing burdens. According to Pojman [43], the general definition for frontal polymerizations is the following: A frontal polymerization is a way to convert liquid resin into a solid material with a self-propagating reaction. In general, the frontal polymerization (FP) technique is a process, in which a localized reaction zone (the so-called polymerization front), once initiated by an external stimulus, acts as a switch for the crosslinking of the reactants in adjacent areas. The process yields uniform network formation throughout the reaction mixture. The allocation of FP techniques into isothermal frontal polymerization (IFP), thermal frontal polymerization (TFP) and frontal photopolymerization (FPP) is based on the external stimuli that are employed to initiate and trigger the reacting front [188].

**Figure 31:**
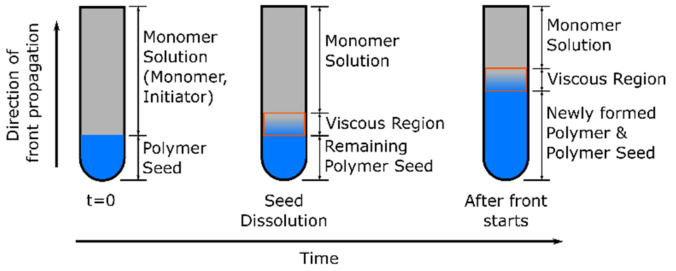
Schematic representation of isothermal frontal polymerizations (IFPs).

Isothermal-FP relies on the so-called “gel effect” or Norrish–Trommsdorff effect [189] that occurs when the monomer and a thermal initiator diffuse into a solid polymer fraction of the reaction mixture (polymer seed). By diffusing into the polymer seed, the solution of monomer and thermal initiator dissolve its topmost layer to create a viscous region that propagates through the reaction vessel (Figure 31). The polymerization occurs in both the monomer solution and the viscous region but occurs faster in the viscous region, which consequently propagates up the reaction vessel.

Reaction termination is inhibited by the high viscosity of the polymerized medium [190]. Isothermal-FP has been successfully used to produce gradient materials by incorporating a dopant material, for instance, a second monomer or dye, into the monomer solution or polymer seed [189]. However, it is limited to resin systems that exhibit the gel effect and whose polymers are soluble in their monomers. Furthermore, isothermal fronts propagate at the order of 1 cm/day and only for total distances of about 1 cm [188].

**Figure 32:**
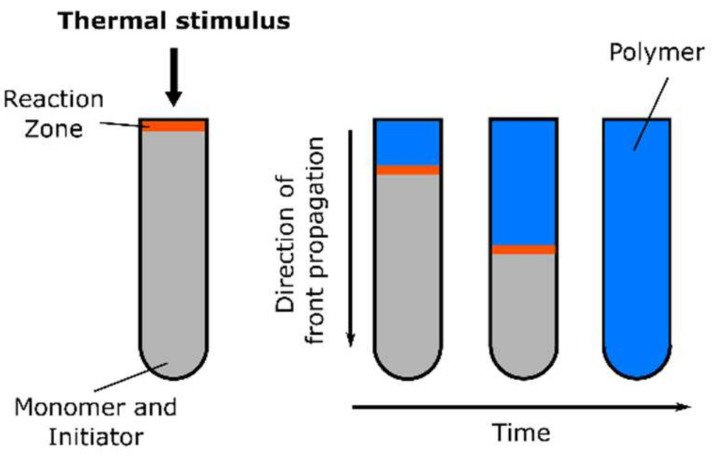
Schematic representation of thermal frontal polymerizations (TFPs) requiring an external stimulus to start an exothermic reaction wave.

Thermal-FP, which can be applied to the widest range of materials, is based on the coupling of thermal transport and the Arrhenius dependence of the reaction rate of an exothermic polymerization [43]. The exothermic reaction causes a self-propagating front that separates the cured polymer and the uncured liquid monomer (Figure 32).

In order to sustain a traveling front, the heat produced by the exothermic reaction must exceed the heat that is lost through the reaction vessel. Furthermore, to prevent the formation of bubbles, the monomer should have a higher boiling point than the front temperature. Thermal frontal polymerization is the most widely studied mechanism, having the widest range of materials to be used [188]. A comprehensive and accessible review of isothermal frontal polymerization and thermal frontal polymerization is given by Pojman et al. [43,188,191,192].

**Figure 33:**
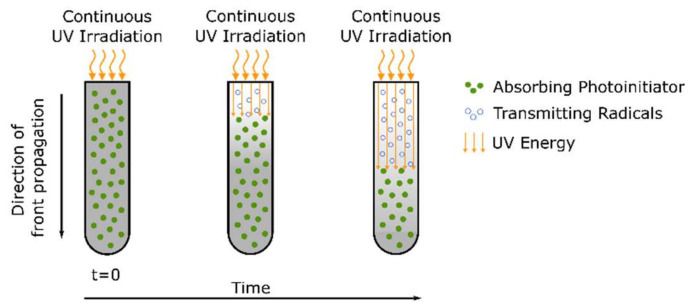
Schematic representation of photofrontal polymerizations showing the photobleaching effect during light exposure.

Photofrontal polymerization is a distinct mode of polymerization from IFP and TFP, which are autocatalytic frontal polymerization reaction processes [193]. It is based on the photobleaching effect and requires the continuous flux of radiation, usually UV light, to create a propagating wavefront of network formation [45] (Figure 33).

According to Rytov et al. [194], the following conditions must be fulfilled for a photochemical reaction to proceed as a typical frontal process: high light absorbance, photoinduced bleaching and restricted mass transfer. Photobleaching is based on the effect that the light absorption of the photoinitiator decay products (photolysis products) is lower than the light absorption of the original photoinitiator molecule, thereby allowing more light to pass through the system and allowing the polymerization front to move steadily towards deeper layers [18]. Examples of photobleaching initiators include benzoin ethyl ether (BEE), solutions of acyl and biacyl phosphine oxides and substituted titanocenes [195]. The major disadvantage of photo-FP is the need for continuous exposure of the reaction mixture to light radiation, where the rate and the degree of conversion depend on the given light intensity. However, as opposed to isothermal and thermal FP, the polymerization can be stopped at any time by simply “turning off the light” and can be reactivated when the light source is started [196].

Recently, mathematical modeling and numerical simulation of frontal polymerization have been gaining more and more attention as they are proven to be a major contribution to the investigation of the various parameters that influence the dynamic phenomenon of frontal polymerization and can help with process optimization [197]. Theoretical approaches to modeling the kinetics of photoinitiated frontal polymerization can be for instance reviewed in publications from Hayki et al. [18], Ivanov [118], Miller et al. [195], Cabral et al. [193], and Decker [1].

The challenges of frontal polymerization include the curing of composites and the fabrication of small components [188]. According to Robertson et al. [198], especially the fabrication of small components is challenging, as much of the heat of polymerization is lost to the environment through air or tooling surfaces. Frontal curing of composites is challenging since a high-volume fraction of fibers is required to produce composite materials with good mechanical properties. The corresponding reduction in resin content consequently reduces the exothermic energy density available for polymerization. Furthermore, the preparation of composites leads to challenges regarding the increased thermal conductivity, compared with the unfilled polymers, and the associated energy losses [174]. In 2021, Hirner et al. [199] investigated UV-induced frontal polymerizations for the preparation of gradient magnetic composites and experimentally quantified the heat losses during the cationic polymerization reactions of an unfilled and filled bisphenol A diglycidyl ether. As filler, magnetite (${\mathrm{Fe}}_{3}O_{4}$) nanoparticles were used. The experimental results were compared to the results gained by modeling and simulation according to the finite element method. The results revealed that the epoxy resins filled with ${\mathrm{Fe}}_{3}O_{4}$ nanoparticles show an increase in the thermal conductivity by a factor of 2.5 compared to the unfilled samples. The significant differences in thermal conductivity of the filled and unfilled resin were also clearly visible in the simulations. During frontal polymerization, the epoxy formulations with ${\mathrm{Fe}}_{3}O_{4}$ nanoparticles showed a faster and more homogeneous heat propagation zone compared to the unfilled samples.

## 7. Discussion

Photopolymerization, in which the initiation of a chemical-physical reaction occurs by the exposure of photosensitive monomers to a high-intensity light source, has become a well-accepted technology for manufacturing polymers and has found a large variety of industrial applications. Photoinitiated polymerizations provide significant advantages over thermal-initiated polymerizations, including fast and controllable reaction rates as well as spatial and temporal control over the formation of the material.

In principle, three different types of polymerization, namely cationic, anionic and free-radical polymerization can be performed. These polymerization reactions are often initiated by thermal input, often requiring high-temperature conditions and solvents. With the development of better photoinitiators, photoinduced polymerizations can be used as more efficient polymerization techniques in terms of temperature conditions and solvent-free polymerizations. This improvement has been achieved mainly due to the development of photoacid generators for cationic polymerizations and in free radical polymerizations due to the development of type I and type II photoinitiator.

The modeling of the curing process can be approached by so-called energetic models or kinetic models (either phenomenologically or mechanistically). Energetic models play a minor role in modeling curing kinetics because, unlike kinetic models, they do not provide information about the degree of cure, which is essential for predicting the mechanical properties of the polymer. Phenomenological models, which are based on empirical or semi-empirical rate laws, are widely employed because they are simple and require a limited number of parameters. However, results obtained from phenomenological models cannot be extrapolated to new initial compositions or curing conditions [73]. Another limitation of phenomenological models is their inability to predict post-curing operations due to diffusion-controlled effects after vitrification [75]. In contrast, mechanistic models are obtained from the balance of chemical species involved in the reaction. Therefore, mechanistic models offer more flexibility to changes in formulation or curing conditions, as they account for the individual species concentrations. Mechanistic models consider the complete scheme of consecutive and competitive reactions (initiation, propagation, termination) which take place during curing, hence providing better prediction and interpretation. The principal disadvantage of mechanistic models is the need for an accurate description of all species and reactions involved in the system which is not trivial due to the complex nature of curing reactions, for both free-radical and cationic photopolymerizations [75]. Corcione et al. [137] state that cationic photopolymerizations are even more complex compared to free-radical photopolymerizations as they are strongly affected by the resin formulation. Hence, in contrast to free-radical photopolymerizations, there are few mechanistic models for photoinitiated cationic photopolymerization [29]. To conclude, the advantages and disadvantages of using mechanistic or phenomenological models to obtain a simple and reliable kinetic model describing the reactions that take place during photopolymerization must be carefully weighed for the particular application.

Section 3 presented different approaches, both mechanistic and phenomenological, to model photoinduced free-radical and cationic polymerization. Free-radical polymerization refers to the process of forming a polymer material via the addition of free radicals whereas, in ionic polymerization, the polymer material is formed using ionic chemical species as initial reactants.

Many researchers have realized detailed studies on the mechanistic modeling of photoinduced free-radical polymerization, see Section 3.1.1. From these approaches, one can draw the conclusion that a complete mechanistic model should contain a very large number of differential equations and requires many kinetic parameters that reflect the curing behavior with sufficient accuracy. Approaches reported in the literature distinguish between pointwise mechanistic and spatially dependent mechanistic models. Unlike pointwise mechanistic models, spatial mechanistic models include the description of the spatial distribution of reactants inside the continuum body during the process. Consequently, for spatially dependent mechanistic models, the differential problem directly provides the evolution of the degree of cure in space and time. According to Christmann et al. [102], kinetic models to study the complex free-radical photopolymerization mechanism and its related effects must consider simultaneously all the termination pathways (bimolecular termination, primary radical termination, and radical trapping) and must not neglect the evolution of terminations along the polymerization reaction. Furthermore, the termination kinetic constant should incorporate all different possible mechanisms that control termination: translational diffusion, segmental diffusion, reaction-diffusion, and chain-length dependent termination [19]. The authors state that all main kinetic constants (propagation, termination) should be modeled considering their progressive diffusional control. Effects such as auto-acceleration and auto-deceleration can only be considered when the rate constants for propagation and termination are modeled as a function of conversion. However, Wu et al. [82] state that the most sensitive to viscosity increase is the constant of the termination rate. In their proposed model, Wu et al. [82] consider non-constant propagation and termination rates with increasing conversion to account for the decreasing molecular mobility in the reaction medium as polymerization proceeds. The model considers termination by radical combination but neglects chain-length dependency, effects of polymer heterogeneity, and radical trapping. A chain-length dependent termination kinetic constant, which assumes that radicals diffuse and terminate according to their chain length, was proposed by Bowman et al. [19]. However, the model does not consider a limiting radical chain length to determine if the radical is “trapped” in the gel and is no longer capable of diffusion-limited termination. Wen et al. [109] note that kinetic models that ignore radical trapping fail to predict important aspects of experimental investigation. Therefore, the authors model the rate constant for radical trapping as a simple function of free volume, following the model of Anseth et al. [38]. The free volume theory of Anseth et al. [38] is also the basis for models proposed for instance by Anastasio et al. [86] and Bowman et al. [19]. The kinetic model described by Anastasio and his co-authors considers the recombination (“trapping”) process by reducing the initiator efficiency.

Another important factor is the modeling of the light intensity which is the driving factor for the formation of free radicals. Some models proposed in the literature assume that the light intensity follows a conventional Beer–Lambert law which describes the light propagation through a homogeneous medium without internal sources of scattering. Therefore, these approaches only consider the light intensity variation with changes in the spatial position of the sample. The abovementioned approach implies the oversimplified assumption that the photolysis product becomes completely transparent after polymerization. In contrast, Lin et al. [112] consider a non-uniform distribution of the photoinitiator (depletion of the photoinitiator concentration) as the UV-light might still be absorbed by the photolysis product, besides the absorption of the monomer.

A critical aspect of modeling the curing kinetics of photoinitiated free-radical polymerization concerns oxygen diffusion effects. Cationic photopolymerizations have the distinct advantage that they lack sensitivity towards atmospheric oxygen, whereas the loss of radicals, known as oxygen inhibition, is pervasive in free-radical photopolymerizations [22]. Oxygen in the reaction volume acts as a “radical scavenger”, i.e., it reduces the efficiency of initiation and generally leads to significant retardation or even inhibition of the polymerization [33]. Therefore, considering the inhibitory effect of oxygen on free-radical photopolymerization plays an important role in correctly modeling curing kinetics and is considered in most kinetic models presented in the literature [22,24,34,82,88,105,115,119,160].

O’Brien et al. [97] pointed out that for more complex polymerization systems including additional factors such as heat generation, heat transfer and mass transfer is necessary. Heat effects play an important role, as the kinetic constants, as well as the diffusion coefficients, are a function of temperature. Therefore, the model proposed by O’Brien et al. [97] includes not only the temporal and spatial distribution of species concentration, temperature and light intensity but also heat and heat-transfer effects.

Due to the complexity of cationic polymerization reactions, in contrast to free-radical polymerization, there are view mechanistic models for photoinitiated cationic polymerization [29]. A mechanistic mathematical model to characterize cationic photopolymerization reactions as a function of temperature and exposure time, proposed by Nelson et al. [135], was used as the basis for more elaborate models which also consider the dependency of the rate of conversion on the light intensity, for instance, models proposed by Corcione et al. [137] and Pantiru et al. [138].

According to Wu et al. [82], in contrast to mechanistic models, in phenomenological models the conversion of the monomer (degree of cure) is the only variable to characterize the polymerization process. Thus, the variation of other species is ignored. The simplest and most common analytical form of the function of the degree of cure, namely the *n*-th order kinetic reaction model, cannot be applied to modeling photopolymerization reactions. The reason lies in the fact, that the *n*-th order kinetic model predicts a maximum of the reaction rate at the start of the reaction (*t* = 0), whereas photopolymerization reactions show a maximum value of the reaction rate at any point, rather than the reaction start point [140]. Therefore, the majority of phenomenological models used to describe photopolymerization reactions are based on the so-called autocatalytic model, proposed by Kamal et al. [120], assuming a reaction rate that is initially equal to zero, showing a maximum value at intermediate conversion. Simple models, proposed for instance by Maffezzoli et al. [127], assuming isothermal kinetic behavior, were improved by describing the maximum attainable curing degree as a function of absolute temperature as well as location- and time-dependent light intensity. The influence of diffusion-controlled effects on the curing reactions were considered in phenomenological models presented, for instance, by Kim et al. [29] and Bartolo [75] who presented a coupled photothermal phenomenological approach to correctly model the physical and chemical changes occurring in the bulk and in the surroundings of the material exposed to UV light, as well as the rates at which these changes occur by describing the temperature field in the region exposed to UV radiation by the heat conduction equation.

Polymer composites have many advantages over traditional polymeric materials in terms of mechanical properties and are therefore widely used in automotive, aerospace and biomedical applications. However, polymer composites are often obtained by heat-curing processes that require high energy input and often long curing times [200]. These disadvantages can be avoided by using the frontal polymerization technique, in which the reaction process is mainly governed by the chemical and physical properties of the reacting system. Frontal polymerization (FP) is a process in which a localized reaction zone (the so-called polymerization front), once initiated by an external stimulus, acts as a switch for the crosslinking of the reactants in adjacent areas. Depending on the external stimuli that are employed to initiate and trigger the reaction front, the following types of frontal polymerization are distinguished: isothermal frontal polymerization (IFP), thermal frontal polymerization (TFP), and frontal photopolymerization (FPP). According to Frulloni et al. [197], recently, mathematical modeling and numerical simulation of frontal polymerization have been gaining more and more attention as they are proven to be a major contribution to the investigation of the various parameters that influence the dynamic phenomenon of frontal polymerization and can help with process optimization.

## 8. Concluding Remarks

In this paper, we have presented an extensive review of processes and models related to photopolymerization reactions. Besides briefly discussing the materials and curing chemistry, the main goal of the review has been to provide a comprehensive overview of analytical models and numerical approaches to accurately describe the cure kinetics and mechanisms for curing behavior simulations of such ultrafast crosslinking polymerization reactions. The correct modeling of diffusion-controlled phenomena as well as the modeling of oxygen diffusion-reaction in free-radical systems, in order to study material property changes during the photopolymerization process, faces both theoretical and numerical challenges and different approaches are used to face these obstacles. Besides briefly discussing the main characteristics of different modeling approaches, the main goal of the review has been to provide a critical and comprehensive overview of the similarities and differences between approaches to describe the photothermal kinetic process of the chemical conversion from monomers in a liquid state to polymeric chains. The review attempts to offer an overall view of the limitations of current modeling, ranging from mechanistic to phenomenological models based on either pointwise or spatial approaches, and suggest possible improvements. Furthermore, approaches regarding the implementation of kinetic models through numerical simulations, proposed in the literature, are presented.

## Data Availability

May be found from the cited references.

## Abbreviations

AIBN	Azobisisbutyronitrile
CADE	Cycloaliphatic Diepoxide
CLDT	Chain Length Dependent Termination
CLIP	Continuous Liquid Interface Production
DCPD	Dicyclopentadiene
DEGDMA	Diethylene Glycol Dimethacrylate
DGEBA	Diglycidyl Ether of Bisphenol A
DGEBF	Diglycidyl Ether of Bisphenol F
DLP	Digital Light Processing
DMPA	Dimethoxy Phenylacetophenone
DSC	Differential Scanning Calorimetry
ECH	Epichlorohydrin
ECPL	Exposure Controlled Projection Lithography
EVE	Ethyl Vinyl Ether
FE	Finite Elements
FEA	Finite Element Analysis
FP	Frontal Polymerization
FPP	Frontal Photo Polymerization
FROMP	Frontal Ring-Opening Metathesis Polymerization
FTIR	Fourier-Transformed Infrared
IBVE	Iso-Butyl Vinyl Ether
IFP	Isothermal Frontal Polymerization
HBVE	Hydroxylbutyl Vinyl Ether
MMA	Methyl Methacrylate
PAG	Photoacid Generator
PEGDA	Poly(ethylene glycol) Diacrylate
PRT	Primary Radical Termination
RICFP	Radical-Induced Frontal Polymerization
RT-FTIR	Real-Time Fourier-Transformed Infrared
TFP	Thermal Frontal Polymerization
TPO	Trimethylbenzoyl diphenylphosphine oxide
UV	Ultraviolet
SLA	Stereolithography Applications
WLF	Williams-Lendel-Ferry

## References

[1] Decker C. (1998). The use of UV irradiation in polymerization. Polym. Int..

[2] Peiffer R.W. (1997). Applications of Photopolymer Technology.

[3] Bednarczyk P., Nowak M., Mozelewska K., Czech Z. (2021). Photocurable Coatings Based on Bio-Renewable Oligomers and Monomers. Materials.

[4] Tytgat L., Markovic M., Qazi T.H., Vagenende M., Bray F., Martins J.C., Rolando C., Thienpont H., Ottevaere H., Ovsianikov A. (2019). Photo-crosslinkable recombinant collagen mimics for tissue engineering applications. J. Mater. Chem. B.

[5] Pereira R.F., Bártolo P.J. (2015). 3D Photo-Fabrication for Tissue Engineering and Drug Delivery. Engineering.

[6] Hutchison J.B., Haraldsson K.T., Good B.T., Sebra R.P., Luo N., Anseth K.S., Bowman C.N. (2004). Robust polymer microfluidic device fabrication via contact liquid photolithographic polymerization (CLiPP). Lab Chip.

[7] Grant Willson C., Trinque B.C. (2003). The Evolution of Materials for the Photolithographic Process. J. Photopol. Sci. Technol..

[8] Rogers J.A., Nuzzo R.G. (2005). Recent progress in soft lithography. Mater. Today.

[9] Del Barrio J., Sánchez-Somolinos C. (2019). Light to Shape the Future: From Photolithography to 4D Printing. Adv. Opt. Mater..

[10] Haraldsson K.T., Hutchison J.B., Sebra R.P., Good B.T., Anseth K.S., Bowman C.N. (2006). 3D polymeric microfluidic device fabrication via contact liquid photolithographic polymerization (CLiPP). Sens. Actuators B Chem..

[11] Xu Y., Qi F., Mao H., Li S., Zhu Y., Gong J., Wang L., Malmstadt N., Chen Y. (2022). In-situ transfer vat photopolymerization for transparent microfluidic device fabrication. Nat. Commun..

[12] Liska R., Schuster M., Inführ R., Turecek C., Fritscher C., Seidl B., Schmidt V., Kuna L., Haase A., Varga F. (2007). Photopolymers for rapid prototyping. J. Coat. Technol. Res..

[13] Rocheva V.V., Koroleva A.V., Savelyev A.G., Khaydukov K.V., Generalova A.N., Nechaev A.V., Guller A.E., Semchishen V.A., Chichkov B.N., Khaydukov E.V. (2018). High-resolution 3D photopolymerization assisted by upconversion nanoparticles for rapid prototyping applications. Sci. Rep..

[14] Klikovits N., Sinawehl L., Knaack P., Koch T., Stampfl J., Gorsche C., Liska R. (2020). UV-Induced Cationic Ring-Opening Polymerization of 2-Oxazolines for Hot Lithography. ACS Macro Lett..

[15] Pagac M., Hajnys J., Ma Q.-P., Jancar L., Jansa J., Stefek P., Mesicek J. (2021). A Review of Vat Photopolymerization Technology: Materials, Applications, Challenges, and Future Trends of 3D Printing. Polymers.

[16] Ligon S.C., Liska R., Stampfl J., Gurr M., Mülhaupt R. (2017). Polymers for 3D Printing and Customized Additive Manufacturing. Chem. Rev..

[17] Shusteff M., Browar A.E.M., Kelly B.E., Henriksson J., Weisgraber T.H., Panas R.M., Fang N.X., Spadaccini C.M. (2017). One-step volumetric additive manufacturing of complex polymer structures. Sci. Adv..

[18] Hayki N., Lecamp L., Désilles N., Lebaudy P. (2010). Kinetic Study of Photoinitiated Frontal Polymerization. Influence of UV Light Intensity Variations on the Conversion Profiles. Macromolecules.

[19] Bowman C.N., Kloxin C.J. (2008). Toward an enhanced understanding and implementation of photopolymerization reactions. AIChE J..

[20] Rusu M.C., Block C., van Assche G., van Mele B. (2012). Influence of temperature and UV intensity on photo-polymerization reaction studied by photo-DSC. J. Therm. Anal. Calorim..

[21] Kaur M., Srivastava A.K. (2002). PHOTOPOLYMERIZATION: A REVIEW. J. Macromol. Sci. Part C Polym. Rev..

[22] Decker C., Jenkins A.D. (1985). Kinetic approach of oxygen inhibition in ultraviolet- and laser-induced polymerizations. Macromolecules.

[23] Lovestead T.M., O’Brien A.K., Bowman C.N. (2003). Models of multivinyl free radical photopolymerization kinetics. J. Photochem. Photobiol. A Chem..

[24] Studer K., Decker C., Beck E., Schwalm R. (2003). Overcoming oxygen inhibition in UV-curing of acrylate coatings by carbon dioxide inerting, Part I. Prog. Org. Coat..

[25] Decker C., Moussa K. (1990). Kinetic study of the cationic photopolymerization of epoxy monomers. J. Polym. Sci. Part A Polym. Chem..

[26] Ficek B.A. (2008). The Potential of Cationic Photopolymerization’s Long Lived Active Centers. Master’s Thesis.

[27] Malik M.S., Schlögl S., Wolfahrt M., Sangermano M. (2020). Review on UV-Induced Cationic Frontal Polymerization of Epoxy Monomers. Polymers.

[28] Esposito Corcione C., Greco A., Maffezzoli A. (2003). Photopolymerization Kinetics of an Epoxy Based Resin for Stereolithography—Calorimetric Analysis. J. Therm. Anal. Calorim..

[29] Kim Y.-M., Kostanski L.K., MacGregor J.F. (2005). Kinetic studies of cationic photopolymerizations of cycloaliphatic epoxide, triethyleneglycol methyl vinyl ether, and cyclohexene oxide. Polym. Eng. Sci..

[30] Lin J.T., Lalevee J., Cheng D.C. (2021). A Critical Review for Synergic Kinetics and Strategies for Enhanced Photopolymerizations for 3D-Printing and Additive Manufacturing. Polymers.

[31] De Beer M.P., Van Der Laan H.L., Cole M.A., Whelan R.J., Burns M.A., Scott T.F. (2019). Rapid, continuous additive manufacturing by volumetric polymerization inhibition patterning. Sci. Adv..

[32] Van Der Laan H.L., Burns M.A., Scott T.F. (2019). Volumetric Photopolymerization Confinement through Dual-Wavelength Photoinitiation and Photoinhibition. ACS Macro Lett..

[33] Andrzejewska E., Lindén L.-Å., Rabek J.F. (1997). Modelling the Kinetics of Photoinitiated Polymerization of Di(meth)acrylates. Polym. Int..

[34] Decker C., Moussa K. (1990). Kinetic investigation of photopolymerizations induced by laser beams. Die Makromol. Chem..

[35] Decker C. (1983). Ultra-fast polymerization of epoxy-acrylate resins by pulsed laser irradiation. J. Polym. Sci. Polym. Chem. Ed..

[36] Lin Y., Stansbury J.W. (2003). Kinetics studies of hybrid structure formation by controlled photopolymerization. Polymer.

[37] O’Shaughnessy B., Yu J. (1994). Autoacceleration in free radical polymerization. Phys. Rev. Lett..

[38] Anseth K.S., Bowman C.N. (1993). Reaction Diffusion Enhanced Termination in Polymerizations of Multifunctional Monomers. Polym. React. Eng..

[39] Brighenti R., Cosma M.P., Marsavina L., Spagnoli A., Terzano M. (2021). Laser-based additively manufactured polymers: A review on processes and mechanical models. J. Mater. Sci..

[40] Davtyan S.P., Berlin A.A., Tonoyan A.O. (2011). Advances and problems of frontal polymerization processes. Ref. J. Chem..

[41] Davtyan S.P., Avetisyan A.S., Berlin A.A., Tonoyan A.O. (2013). Synthesis and properties of particle-filled and intercalated polymer nanocomposites. Ref. J. Chem..

[42] Cui Y., Yang J., Zhan Y., Zeng Z., Chen Y. (2008). In situ fabrication of polyacrylate/nanozirconia hybrid material via frontal photopolymerization. Colloid Polym. Sci..

[43] Pojman J.A. (2019). Mathematical modeling of frontal polymerization. Math. Model. Nat. Phenom..

[44] Garra P., Dietlin C., Morlet-Savary F., Dumur F., Gigmes D., Fouassier J.-P., Lalevée J. (2017). Photopolymerization processes of thick films and in shadow areas: A review for the access to composites. Polym. Chem..

[45] Ebner C., Mitterer J., Eigruber P., Stieger S., Riess G., Kern W. (2020). Ultra-High Through-Cure of (Meth)Acrylate Copolymers via Photofrontal Polymerization. Polymers.

[46] Carion P., Ibrahim A., Allonas X., Croutxé-Barghorn C., L’Hostis G. (2019). Frontal free-radical photopolymerization of thick samples: Applications to LED-induced fiber-reinforced polymers. J. Polym. Sci. Part A Polym. Chem..

[47] Moad G., Solomon D.H. (2006). The Chemistry of Radical Polymerization.

[48] Matyjaszewski K., Davis T.P. (2003). Handbook of Radical Polymerization.

[49] Guerrero-Santos R., Saldívar-Guerra E., Bonilla-Cruz J., Saldívar-Guerra E., Vivaldo-Lima E. (2013). Free Radical Polymerization. Handbook of Polymer Synthesis, Characterization, and Processing.

[50] Elias H.-G. (2009). Makromoleküle: Chemische Struktur und Synthesen-Sechste.

[51] Odian G.G. (2010). Principles of Polymerization.

[52] Fouassier J., Allonas X., Burget D. (2003). Photopolymerization reactions under visible lights: Principle, mechanisms and examples of applications. Prog. Org. Coat..

[53] Elian C., Brezová V., Sautrot-Ba P., Breza M., Versace D.-L. (2021). Lawsone Derivatives as Efficient Photopolymerizable Initiators for Free-Radical, Cationic Photopolymerizations, and Thiol-Ene Reactions. Polymers.

[54] Daglen B.C., Tyler D.R. (2010). Photodegradable plastics: End-of-life design principles. Green Chem. Lett. Rev..

[55] Yagci Y., Jockusch S., Turro N.J. (2010). Photoinitiated Polymerization: Advances, Challenges, and Opportunities. Macromolecules.

[56] Ibrahim A., Stefano L., Tarzi O., Tar H., Ley C., Allonas X. (2013). High-performance photoinitiating systems for free radical photopolymerization. Application to holographic recording. Photochem. Photobiol..

[57] Schlüter A.-D. (1998). Synthesis of Polymers.

[58] Sangermano M. (2012). Advances in cationic photopolymerization. Pure Appl. Chem..

[59] Crivello J.V. (2009). Design of Photoacid Generating Systems. J. Photopol. Sci. Technol..

[60] Crivello J.V., Lee J.L. (1989). Alkoxy-substituted diaryliodonium salt cationic photoinitiators. J. Polym. Sci. Part A Polym. Chem..

[61] Akhtar S.R., Crivello J.V., Lee J.L. (1990). Synthesis of aryl-substituted sulfonium salts by the phosphorus pentoxide-methanesulfonic acid promoted condensation of sulfoxides with aromatic compounds. J. Org. Chem..

[62] Vitale A., Sangermano M., Bongiovanni R., Burtscher P., Moszner N. (2014). Visible Light Curable Restorative Composites for Dental Applications Based on Epoxy Monomer. Materials.

[63] Barner-Kowollik C. (2009). Acrylate free radical polymerization: From mechanism to polymer design. Macromol. Rapid Commun..

[64] Tripathy R., Crivello J.V., Faust R. (2013). Photoinitiated polymerization of acrylate, methacrylate, and vinyl ether end-functional polyisobutylene macromonomers. J. Polym. Sci. Part A Polym. Chem..

[65] Chen J., Chu N., Zhao M., Jin F.-L., Park S.-J. (2020). Synthesis and application of thermal latent initiators of epoxy resins: A review. J. Appl. Polym. Sci..

[66] Brydson J.A. (1999). Epoxide Resins. Plastics Materials.

[67] McGrath J.E., McGrath J.E. (1985). Ring-Opening Polymerization: Introduction. Ring-Opening Polymerization.

[68] Sanda F., Endo T. (2001). Radical ring-opening polymerization. J. Polym. Sci. Part A Polym. Chem..

[69] Higashimura T., Sawamoto M. (1989). Carbocationic Polymerization: Vinyl Ethers. Comprehensive Polymer Science and Supplements.

[70] Kammiyada H., Ouchi M., Sawamoto M. (2017). A Study on Physical Properties of Cyclic Poly(vinyl ether)s Synthesized via Ring-Expansion Cationic Polymerization. Macromolecules.

[71] Yamaguchi K., Nakamoto T. (1998). Micro Fabrication by UV Laser Photopolymerization.

[72] Tomeckova V., Halloran J.W. (2010). Predictive models for the photopolymerization of ceramic suspensions. J. Eur. Ceram. Soc..

[73] Blanco M., Corcuera M.A., Riccardi C.C., Mondragon I. (2005). Mechanistic kinetic model of an epoxy resin cured with a mixture of amines of different functionalities. Polymer.

[74] Matias J.M., Bartolo P.J., Pontes A.V. (2009). Modeling and simulation of photofabrication processes using unsaturated polyester resins. J. Appl. Polym. Sci..

[75] Da Silva Bartolo P.J. (2007). Photo-curing modelling: Direct irradiation. Int. J. Adv. Manuf. Technol..

[76] Han C.D., Lee D.-S. (1987). Analysis of the curing behavior of unsaturated polyester resins using the approach of free radical polymerization. J. Appl. Polym. Sci..

[77] Nogueira T.R., Goncalves M.C., Ferrareso Lona L.M., Vivaldo-Lima E., McManus N., Penlidis A. (2010). Effect of initiator type and concentration on polymerization rate and molecular weight in the bimolecular nitroxide-mediated radical polymerization of styrene. Adv. Polym. Technol. J. Polym. Processing Inst..

[78] Gillespie D.T. (1977). Exact stochastic simulation of coupled chemical reactions. J. Phys. Chem..

[79] Altun-Çiftçioğlu G.A., Ersoy-Meriçboyu A., Henderson C.L. (2011). Stochastic modeling and simulation of photopolymerization process. Polym. Eng. Sci..

[80] Boddapati A. (2010). Modeling Cure Depth during Photopolymerization of Multifunctional Acrylates. Ph.D Thesis.

[81] Goodner M.D., Bowman C.N. (1999). Modeling Primary Radical Termination and Its Effects on Autoacceleration in Photopolymerization Kinetics. Macromolecules.

[82] Wu J., Zhao Z., Hamel C.M., Mu X., Kuang X., Guo Z., Qi H.J. (2018). Evolution of material properties during free radical photopolymerization. J. Mech. Phys. Solids.

[83] Zhang Y., Kranbuehl D.E., Sautereau H., Seytre G., Dupuy J. (2009). Modeling and Measuring UV Cure Kinetics of Thick Dimethacrylate Samples. Macromolecules.

[84] Achilias D.S. (2007). A Review of Modeling of Diffusion Controlled Polymerization Reactions. Macromol. Theory Simul..

[85] Batch G.L., Macosko C.W. (1992). Kinetic model for crosslinking free radical polymerization including diffusion limitations. J. Appl. Polym. Sci..

[86] Anastasio R., Peerbooms W., Cardinaels R., van Breemen L.C.A. (2019). Characterization of Ultraviolet-Cured Methacrylate Networks: From Photopolymerization to Ultimate Mechanical Properties. Macromolecules.

[87] Long K.N., Scott T.F., Jerry Qi H., Bowman C.N., Dunn M.L. (2009). Photomechanics of light-activated polymers. J. Mech. Phys. Solids.

[88] O’Brien A.K., Bowman C.N. (2006). Modeling the Effect of Oxygen on Photopolymerization Kinetics. Macromol. Theory Simul..

[89] Brighenti R., Cosma M.P., Marsavina L., Spagnoli A., Terzano M. (2021). Multiphysics modelling of the mechanical properties in polymers obtained via photo-induced polymerization. Int. J. Adv. Manuf. Technol..

[90] Buback M., Huckestein B., Russell G.T. (1994). Modeling of termination in intermediate and high conversion free radical polymerizations. Macromol. Chem. Phys..

[91] Buback M., Hesse P., Hutchinson R.A., Kasák P., Lacík I., Stach M., Utz I. (2008). Kinetics and Modeling of Free-Radical Batch Polymerization of Nonionized Methacrylic Acid in Aqueous Solution. Ind. Eng. Chem. Res..

[92] Buback M. (1990). Free-radical polymerization up to high conversion. A general kinetic treatment. Die Makromol. Chem..

[93] Dickey M.D., Willson C.G. (2006). Kinetic parameters for step and flash imprint lithography photopolymerization. AIChE J..

[94] Gan S., Seferis J.C., Prime R.B. (1991). A viscoelastic description of the glass transition-conversion relationship for reactive polymers. J. Therm. Anal. Calorim..

[95] Goodner M.D., Bowman C.N. (2002). Development of a comprehensive free radical photopolymerization model incorporating heat and mass transfer effects in thick films. Chem. Eng. Sci..

[96] Goodner M.D., Lee H.R., Bowman C.N. (1997). Method for Determining the Kinetic Parameters in Diffusion-Controlled Free-Radical Homopolymerizations. Ind. Eng. Chem. Res..

[97] O’Brien A.K., Bowman C.N. (2003). Modeling Thermal and Optical Effects on Photopolymerization Systems. Macromolecules.

[98] Lovestead T.M., Berchtold K.A., Bowman C.N. (2002). Modeling the Effects of Chain Length on the Termination Kinetics in Multivinyl Photopolymerizations. Macromol. Theory Simul..

[99] Bowman C.N., Peppas N.A. (1991). Coupling of kinetics and volume relaxation during polymerizations of multiacrylates and multimethacrylates. Macromolecules.

[100] Zhu S., Tian Y., Hamielec A.E., Eaton D.R. (1990). Radical trapping and termination in free-radical polymerization of methyl methacrylate. Macromolecules.

[101] Anseth K.S., Wang C.M., Bowman C.N. (1994). Kinetic evidence of reaction diffusion during the polymerization of multi(meth)acrylate monomers. Macromolecules.

[102] Christmann J., Ley C., Allonas X., Ibrahim A., Croutxé-Barghorn C. (2019). Experimental and theoretical investigations of free radical photopolymerization: Inhibition and termination reactions. Polymer.

[103] Ibrahim A., Maurin V., Ley C., Allonas X., Croutxe-Barghorn C., Jasinski F. (2012). Investigation of termination reactions in free radical photopolymerization of UV powder formulations. Eur. Polym. J..

[104] Gao Y., Xu L., Zhao Y., You Z., Guan Q. (2020). 3D printing preview for stereo-lithography based on photopolymerization kinetic models. Bioact. Mater..

[105] Wang J., Zhao C., Zhang Y., Jariwala A., Rosen D. Process Modeling and In-Situ Monitoring of Polymerization for Exposure Controlled Projection Lithography (ECPL). Proceedings of the 28th Annual International Solid Free Form Fabrication Symposium—An Additive Manufacturing Conference.

[106] Lee J.H., Prud’homme R.K., Aksay I.A. (2001). Cure depth in photopolymerization: Experiments and theory. J. Mater. Res..

[107] Dickey M.D., Burns R.L., Kim E.K., Johnson S.C., Stacey N.A., Willson C.G. (2005). Study of the kinetics of step and flash imprint lithography photopolymerization. AIChE J..

[108] Perry M.F., Young G.W. (2005). A Mathematical Model for Photopolymerization From a Stationary Laser Light Source. Macromol. Theory Simul..

[109] Wenand M., McCormick A.V. (2000). A Kinetic Model for Radical Trapping in Photopolymerization of Multifunctional Monomers. Macromolecules.

[110] Anseth K.S., Anderson K.J., Bowman C.N. (1996). Radical concentrations, environments, and reactivities during crosslinking polymerizations. Macromol. Chem. Phys..

[111] Bueche F. (1962). Physical Properties of Polymers.

[112] Lin J.-T., Wang K.-C. (2016). Analytic formulas and numerical simulations for the dynamics of thick and non-uniform polymerization by a UV light. J. Polym. Res..

[113] Lin J.-T., Cheng D.-C. (2017). Modeling the efficacy profiles of UV-light activated corneal collagen crosslinking. PLoS ONE.

[114] Lin J.-T., Liu H.-W., Chen K.-T., Cheng D.-C. (2019). Modeling the Optimal Conditions for Improved Efficacy and Crosslink Depth of Photo-Initiated Polymerization. Polymers.

[115] Lin J.-T., Liu H.-W., Chen K.-T., Cheng D.-C. (2019). Modeling the Kinetics, Curing Depth, and Efficacy of Radical-Mediated Photopolymerization: The Role of Oxygen Inhibition, Viscosity, and Dynamic Light Intensity. Front. Chem..

[116] Lin J.-T., Cheng D.-C., Chen K.-T., Chiu Y.-C., Liu H.-W. (2020). Enhancing UV Photopolymerization by a Red-light Preirradiation: Kinetics and Modeling Strategies for Reduced Oxygen Inhibition. J. Polym. Sci..

[117] Lin J.-T., Lee Y.-Z., Lalevee J., Kao C.-H., Lin K.-H., Cheng D.-C. (2022). Modeling the Enhanced Efficacy and Curing Depth of Photo-Thermal Dual Polymerization in Metal (Fe) Polymer Composites for 3D Printing. Polymers.

[118] Ivanov V.V., Decker C. (2001). Kinetic study of photoinitiated frontal polymerization. Polym. Int..

[119] Childress K.K., Kim K., Glugla D.J., Musgrave C.B., Bowman C.N., Stansbury J.W. (2019). Independent Control of Singlet Oxygen and Radical Generation via Irradiation of a Two-Color Photosensitive Molecule. Macromolecules.

[120] Kamal M.R., Sourour S. (1973). Kinetics and thermal characterization of thermoset cure. Polym. Eng. Sci..

[121] Sourour S., Kamal M.R. (1976). Differential scanning calorimetry of epoxy cure: Isothermal cure kinetics. Thermochim. Acta.

[122] Harikrishna R., Ponrathnam S., Tambe S.S. (2014). Reaction kinetics and modeling of photoinitiated cationic polymerization of an alicyclic based diglycidyl ether. Nucl. Instrum. Methods Phys. Res. Sect. B Beam Interact. Mater. At..

[123] Atai M., Watts D.C. (2006). A new kinetic model for the photopolymerization shrinkage-strain of dental composites and resin-monomers. Dent. Mater..

[124] Khudyakov I.V., Legg J.C., Purvis M.B., Overton B.J. (1999). Kinetics of Photopolymerization of Acrylates with Functionality of 1−6. Ind. Eng. Chem. Res..

[125] Cook W.D. (1992). Photopolymerization kinetics of dimethacrylates using the camphorquinone/amine initiator system. Polymer.

[126] Ganglani M., Carr S.H., Torkelson J.M. (2002). Influence of cure via network structure on mechanical properties of a free-radical polymerizing thermoset. Polymer.

[127] Maffezzoli A., Terzi R. (1998). Effect of irradiation intensity on the isothermal photopolymerization kinetics of acrylic resins for stereolithography. Thermochim. Acta.

[128] Rehbein T., Lion A., Johlitz M., Constantinescu A. (2020). Experimental investigation and modelling of the curing behaviour of photopolymers. Polym. Test..

[129] Bartolo P.J.d.S. (2001). Optical Approaches to Macroscopic and Microscopic Engineering. Ph.D. Thesis.

[130] Yang Y., Li L., Zhao J. (2019). Mechanical property modeling of photosensitive liquid resin in stereolithography additive manufacturing: Bridging degree of cure with tensile strength and hardness. Mater. Des..

[131] Kenny J.M., Trivisano A. (1991). Isothermal and dynamic reaction kinetics of high performance epoxy matrices. Polym. Eng. Sci..

[132] Park I.-K., Lee D.-S., Nam J.-D. (2002). Equivalent processing time analysis of glass transition development in epoxy/carbon fiber composite systems. J. Appl. Polym. Sci..

[133] Kim Y.C., Hong S., Sun H., Kim M.G., Choi K., Cho J., Choi H.R., Koo J.C., Moon H., Byun D. (2017). UV-curing kinetics and performance development of in situ curable 3D printing materials. Eur. Polym. J..

[134] Nelson E.W., Jacobs J.L., Scranton A.B., Anseth K.S., Bowman C.N. (1995). Photo-differential scanning calorimetry studies of cationic polymerizations of divinyl ethers. Polymer.

[135] Nelson E.W., Scranton A.B. (1996). Kinetics of cationic photopolymerizations of divinyl ethers characterized usingin situ Raman spectroscopy. J. Polym. Sci. Part A Polym. Chem..

[136] Nelson E.W., Carter T.P., Scranton A.B. (1995). The role of the triplet state in the photosensitization of cationic polymerizations by anthracene. J. Polym. Sci. Part A Polym. Chem..

[137] Corcione C.E., Greco A., Maffezzoli A. (2005). Time–temperature and time-irradiation intensity superposition for photopolymerization of an epoxy based resin. Polymer.

[138] Pantiru M., Vuluga D.M., Vasilescu D.S., Abadie M.J.M. (2002). Study of the cationic photopolymerization kinetics of cyclic acetals. Polym. Bull..

[139] Van Assche G., Swier S., van Mele B. (2002). Modeling and experimental verification of the kinetics of reacting polymer systems. Thermochim. Acta.

[140] Kamal M.R. (1974). Thermoset characterization for moldability analysis. Polym. Eng. Sci..

[141] Rabinowitch E. (1937). Collision, co-ordination, diffusion and reaction velocity in condensed systems. Trans. Faraday Soc..

[142] Williams M.L., Landel R.F., Ferry J.D. (1955). The Temperature Dependence of Relaxation Mechanisms in Amorphous Polymers and Other Glass-forming Liquids. J. Am. Chem. Soc..

[143] Wisanrakkit G., Gillham J.K. (1990). The glass transition temperature (Tg) as an index of chemical conversion for a high-Tg amine/epoxy system: Chemical and diffusion-controlled reaction kinetics. J. Appl. Polym. Sci..

[144] Cho J.-D., Hong J.-W. (2005). Photo-curing kinetics for the UV-initiated cationic polymerization of a cycloaliphatic diepoxide system photosensitized by thioxanthone. Eur. Polym. J..

[145] Levenberg K. (1944). A method for the solution of certain non-linear problems in least squares. Quart. Appl. Math..

[146] Marquardt D.W. (1963). An Algorithm for Least-Squares Estimation of Nonlinear Parameters. J. Soc. Ind. Appl. Math..

[147] Boey F., Qiang W. (2000). Experimental modeling of the cure kinetics of an epoxy-hexaanhydro-4-methylphthalicanhydride (MHHPA) system. Polymer.

[148] Abadie M.J.M., Chia N.K., Boey F. (2002). Cure kinetics for the ultraviolet cationic polymerization of cycloliphatic and diglycidyl ether of bisphenol-A (DGEBA) epoxy systems with sulfonium salt using an auto catalytic model. J. Appl. Polym. Sci..

[149] Macan J., Ivanković H., Ivanković M., Mencer H.J. (2004). Study of cure kinetics of epoxy-silica organic–inorganic hybrid materials. Thermochim. Acta.

[150] Golaz B., Michaud V., Leterrier Y., Månson J.-A. (2012). UV intensity, temperature and dark-curing effects in cationic photo-polymerization of a cycloaliphatic epoxy resin. Polymer.

[151] Šesták J., Berggren G. (1971). Study of the kinetics of the mechanism of solid-state reactions at increasing temperatures. Thermochim. Acta.

[152] Voytekunas V.Y., Ng F.L., Abadie M.J. (2008). Kinetics study of the UV-initiated cationic polymerization of cycloaliphatic diepoxide resins. Eur. Polym. J..

[153] Jiang F., Drummer D. (2020). Curing Kinetic Analysis of Acrylate Photopolymer for Additive Manufacturing by Photo-DSC. Polymers.

[154] Xu W., Bao S., Shen S., Wang W., Hang G., He P. (2003). Differential scanning calorimetric study on the curing behavior of epoxy resin/diethylenetriamine/organic montmorillonite nanocomposite. J. Polym. Sci. B Polym. Phys..

[155] Chen D.Z., He P.S., Pan L.J. (2003). Cure kinetics of epoxy-based nanocomposites analyzed by Avrami theory of phase change. Polym. Test..

[156] Murias P., Byczyński Ł., Maciejewski H., Galina H. (2015). A quantitative approach to dynamic and isothermal curing of an epoxy resin modified with oligomeric siloxanes. J. Therm. Anal. Calorim..

[157] Pollard M., Kardos J.L. (1987). Analysis of epoxy resin curing kinetics using the Avrami theory of phase change. Polym. Eng. Sci..

[158] Marschik C., Roland W., Löw-Baselli B., Steinbichler G. Application of Hybrid Modeling in Polymer Processing. Proceedings of the Annual Technical Conference for Plastic Professionals (SPE ANTEC).

[159] Taki K. (2020). A Simplified 2D Numerical Simulation of Photopolymerization Kinetics and Oxygen Diffusion-Reaction for the Continuous Liquid Interface Production (CLIP) System. Polymers.

[160] Taki K., Watanabe Y., Ito H., Ohshima M. (2014). Effect of Oxygen Inhibition on the Kinetic Constants of the UV-Radical Photopolymerization of Diurethane Dimethacrylate/Photoinitiator Systems. Macromolecules.

[161] Taki K., Watanabe Y., Tanabe T., Ito H., Ohshima M. (2017). Oxygen concentration and conversion distributions in a layer-by-layer UV-cured film used as a simplified model of a 3D UV inkjet printing system. Chem. Eng. Sci..

[162] Gao K., Ingenhut B.L.J., van de Ven A.P.A., Valega Mackenzie F.O., ten Cate A.T. Multiphysics Modeling of Photo-Polymerization in Stereolithography Printing Process and Validation. Proceedings of the 2018 COMSOL Conference in Lausanne.

[163] Mizukami Y., Rajniak D., Rajniak A., Nishimura M. (2002). A novel microchip for capillary electrophoresis with acrylic microchannel fabricated on photosensor array. Sens. Actuators B Chem..

[164] Erdmann L. (2005). MOEMS-based lithography for the fabrication of micro-optical components. J. Micro/Nanolithography MEMS MOEMS.

[165] Jariwala A.S., Ding F., Zhao X., Rosen D.W. (2008). A Film Fabrication Process on Transparent Substrate Using Mask Projection Micro-Stereolithography.

[166] Jariwala A.S., Ding F., Boddapati A., Breedveld V., Grover M.A., Henderson C.L., Rosen D.W. (2011). Modeling effects of oxygen inhibition in mask-based stereolithography. Rapid Prototyp. J..

[167] Huang Y., Kormakov S., He X., Gao X., Zheng X., Liu Y., Sun J., Wu D. (2019). Conductive polymer composites from renewable resources: An overview of preparation, properties, and applications. Polymers.

[168] Zhang X., Fujiwara S., Fujii M. (2000). Measurements of Thermal Conductivity and Electrical Conductivity of a Single Carbon Fiber. Int. J. Thermophys..

[169] Morak M., Marx P., Gschwandl M., Fuchs P.F., Pfost M., Wiesbrock F. (2018). Heat Dissipation in Epoxy/Amine-Based Gradient Composites with Alumina Particles: A Critical Evaluation of Thermal Conductivity Measurements. Polymers.

[170] Moradi S., Calventus Y., Román F., Hutchinson J.M. (2019). Achieving High Thermal Conductivity in Epoxy Composites: Effect of Boron Nitride Particle Size and Matrix-Filler Interface. Polymers.

[171] Plesa I., Notingher P.V., Schlögl S., Sumereder C., Muhr M. (2016). Properties of Polymer Composites Used in High-Voltage Applications. Polymers.

[172] Kochetov R., Andritsch T., Lafont U., Morshuis P., Smit J.J. (2009). Thermal Conductivity of Nano-Filled Epoxy Systems. 2009 Annual Report, Proceedings of the Conference on Electrical Insulation and Dielectric Phenomena, CEIDP 2009: Virginia Beach, VA, USA, 18–21 October 2009.

[173] Huang X., Jiang P., Tanaka T. (2011). A review of dielectric polymer composites with high thermal conductivity. IEEE Elects. Insul. Mag..

[174] Windberger M.S., Dimitriou E., Rendl S., Wewerka K., Wiesbrock F. (2020). Temperature-Triggered/Switchable Thermal Conductivity of Epoxy Resins. Polymers.

[175] Ma H., Gao B., Wang M., Yuan Z., Shen J., Zhao J., Feng Y. (2021). Strategies for enhancing thermal conductivity of polymer-based thermal interface materials: A review. J. Mater. Sci..

[176] Friedrich K., Breuer U. (2015). Multifunctionality of Polymer Composites: Challenges and New Solutions.

[177] Unterweger C., Brüggemann O., Fürst C. (2014). Synthetic fibers and thermoplastic short-fiber-reinforced polymers: Properties and characterization. Polym. Compos..

[178] Rajak D.K., Pagar D.D., Menezes P.L., Linul E. (2019). Fiber-Reinforced Polymer Composites: Manufacturing, Properties, and Applications. Polymers.

[179] Holbery J., Houston D. (2006). Natural-fiber-reinforced polymer composites in automotive applications. JOM.

[180] Tan D., Irwin P., Sikalidis C. (2011). Polymer Based Nanodielectric Composites. Advances in Ceramics: Electric and Magnetic Ceramics, Bioceramics, Ceramics and Environment.

[181] Song W., Han B., Zhang D., Sun Z., Wang X., Lei Q. (2015). Preparation and properties of BiFeO3/LDPE nanocomposite. Proceedings of the 2015 IEEE 11th International Conference on the Properties and Applications of Dielectric Materials (ICPADM 2015).

[182] Keith Nelson J. (2007). Overview of Nanodielectrics: Insulating Materials of the Future. Proceedings of the 2007 Electrical Insulation Conference and Electrical Manufacturing Expo (EIC/EME).

[183] Qiao Y., Islam M.S., Wang L., Yan Y., Zhang J., Benicewicz B.C., Ploehn H.J., Tang C. (2014). Thiophene Polymer-Grafted Barium Titanate Nanoparticles toward Nanodielectric Composites. Chem. Mater..

[184] King R.W.P. (2000). Electric currents and fields induced in cells in the human brain by radiation from hand-held cellular telephones. J. Appl. Phys..

[185] Gandhi O.P. (2002). Electromagnetic fields: Human safety issues. Annu. Rev. Biomed. Eng..

[186] Roh J.-S., Chi Y.-S., Kang T.J., Nam S. (2008). Electromagnetic Shielding Effectiveness of Multifunctional Metal Composite Fabrics. Text. Res. J..

[187] Sathish Kumar K., Rengaraj R., Venkatakrishnan G.R., Chandramohan A. (2021). Polymeric materials for electromagnetic shielding—A review. Mater. Today Proc..

[188] Pojman J.A., Moeller M., Matyjaszewski K. (2012). Frontal Polymerization. Polymer Science.

[189] Lewis L.L., DeBisschop C.S., Pojman J.A., Volpert V.A. (2005). Isothermal frontal polymerization: Confirmation of the mechanism and determination of factors affecting the front velocity, front shape, and propagation distance with comparison to mathematical modeling. J. Polym. Sci. Part A Polym. Chem..

[190] Warren J.A., Cabral J.T., Douglas J.F. (2005). Solution of a field theory model of frontal photopolymerization. Phys. Rev. E Stat. Nonlin. Soft Matter Phys..

[191] Pojman J.A., Ilyashenko V.M., Khan A.M. (1996). Free-radical frontal polymerization: Self-propagating thermal reaction waves. J. Chem. Soc. Faraday Trans..

[192] Khan A.M., Pojman J.A. (1996). The Use of Frontal Polymerization in Polymer Synthesis. Trends Polym. Sci..

[193] Cabral J.T., Douglas J.F. (2005). Propagating waves of network formation induced by light. Polymer.

[194] Rytov B.L., Ivanov V.B., Ivanov V.V., Anisimov V.M. (1996). Mechanisms of front propagation of photochemical reactions in polymer containing media: 1. Frontal regimes of photochemical reactions in polymer matrices with bleaching of specimen behind the front. Polymer.

[195] Miller G.A., Gou L., Narayanan V., Scranton A.B. (2002). Modeling of photobleaching for the photoinitiation of thick polymerization systems. J. Polym. Sci. Part A Polym. Chem..

[196] Petko F., Świeży A., Ortyl J. (2021). Photoinitiating systems and kinetics of frontal photopolymerization processes—The prospects for efficient preparation of composites and thick 3D structures. Polym. Chem..

[197] Frulloni E., Salinas M.M., Torre L., Mariani A., Kenny J.M. (2005). Numerical modeling and experimental study of the frontal polymerization of the diglycidyl ether of bisphenol A/diethylenetriamine epoxy system. J. Appl. Polym. Sci..

[198] Robertson I.D., Yourdkhani M., Centellas P.J., Aw J.E., Ivanoff D.G., Goli E., Lloyd E.M., Dean L.M., Sottos N.R., Geubelle P.H. (2018). Rapid energy-efficient manufacturing of polymers and composites via frontal polymerization. Nature.

[199] Slugovc C., Trimmel G. (2021). Polymer Meeting 14—Book of Abstracts.

[200] Ghosh A.K., Dwivedi M. (2020). Advantages and Applications of Polymeric Composites. Processability of Polymeric Composites.

